# Noncoding RNA Ginir functions as an oncogene by associating with centrosomal proteins

**DOI:** 10.1371/journal.pbio.2004204

**Published:** 2018-10-08

**Authors:** Suchismita Panda, Meenakshi Setia, Navjot Kaur, Varsha Shepal, Vivek Arora, Divya Kumari Singh, Abir Mondal, Abhishek Teli, Madhura Tathode, Rajendra Gajula, L. C. Padhy, Anjali Shiras

**Affiliations:** 1 National Centre for Cell Science (NCCS), Savitribai Phule Pune University Campus, Pune, India; 2 Bionivid Technology Pvt Ltd, Bangalore, India; 3 Kalinga Institute of Industrial Technology, (KIIT), Bhubaneswar, India; University of Cambridge, United Kingdom of Great Britain and Northern Ireland

## Abstract

Long noncoding RNAs constitute a major fraction of the eukaryotic transcriptome, and together with proteins, they intricately fine-tune various growth regulatory signals to control cellular homeostasis. Here, we describe the functional characterisation of a novel pair of long intergenic noncoding RNAs (lincRNAs) comprised of complementary, fully overlapping sense and antisense transcripts Genomic Instability Inducing RNA (Ginir) and antisense RNA of Ginir (Giniras), respectively, from mouse cells. This transcript pair is expressed in a spatiotemporal manner during embryonic development. The individual levels of the sense and antisense transcripts are finely balanced during embryonic growth and in adult tissues. Functional studies of the individual transcripts performed using overexpression and knock-down strategies in mouse cells has led to the discovery that Ginir RNA is a regulator of cellular proliferation and can act as an oncogene having a preeminent role in malignant transformation. Mechanistically, we demonstrate that the oncogenic function of Ginir is mediated by its interaction with centrosomal protein 112 (Cep112). Additionally, we establish here a specific interaction between Cep112 with breast cancer type 1 susceptibility protein (Brca1), another centrosome-associated protein. Next, we prove that the mutual interaction between Cep112 with Brca1 is significant for mitotic regulation and maintenance of genomic stability. Furthermore, we demonstrate that the Cep112 protein interaction with Brca1 protein is impaired when an elevated level of Ginir RNA is present in the cells, resulting in severe deregulation and abnormality in mitosis, leading to malignant transformation. Inhibiting the Ginir RNA function in transformed cells attenuates transformation and restores genomic stability. Together, these findings unravel, to our knowledge, a hitherto-unknown mechanism of oncogenesis mediated by a long noncoding RNA and establishes a unique role of Cep112–Brca1 interaction being modulated by Ginir RNA in maintaining mitotic fidelity.

## Author summary

The growth of multicellular organisms is tightly regulated by cellular homeostasis mediated by cell division. This is achieved with the help of various proteins acting in a highly coordinated manner via intricately woven intercellular signalling pathways, which regulate cell division. Here, we identify a long noncoding RNA pair, which we named Genomic Instability Inducing RNA (Ginir)/antisense RNA of Ginir (Giniras), and explore its function in cellular homeostasis. We show that this RNA pair is expressed in a spatiotemporally regulated manner during development and is enriched in the brain. We find that Ginir acts as a dominant oncogene when Ginir transcript levels are overexpressed in mouse fibroblasts and that centrosomal protein 112 (Cep112) is its interacting protein partner. We also report that Cep112 interacts with breast cancer type 1 susceptibility protein (Brca1), a protein well known for its role in genome surveillance. Our data reveal that interactions between these two proteins are perturbed in the presence of excessive levels of Ginir RNA, which results in aberrant mitosis and drives the cells towards neoplastic transformation.

## Introduction

The recent surge of information regarding evolutionary conservation, functionality, and annotation of sequences from the mammalian genome has revealed that a bulk of the transcriptome is noncoding and includes small and long noncoding RNAs (lncRNAs). FANTOM data sets of the human and mouse transcriptomes have emphasised that about 63% of the mammalian genome is pervasively transcribed, even from retrotransposon elements, and amongst them, more than 73% of genes show some form of antisense transcription [1,2]. The advances made on the analyses of various transcriptomes followed by identification of several lncRNAs have led to the exposition of their regulatory roles in transcription [3], posttranscriptional gene silencing (PTGS) [4], organogenesis [5], pluripotency, reprogramming, differentiation [6], and also epigenetic regulation [7]. A multitude of lncRNAs have pathological roles in a variety of disease processes [8]. In particular, deregulations of lncRNAs have been correlated to several diseases that include α-thalassaemia [9], myotonic dystrophy [10], Alzheimer disease [11], spinocerebellar ataxia type 8 [12], and also various cancers that include tumours of the central nervous system (CNS) [13], mammary gland [14], colon [15], skin [16], lung [17], and many more. Furthermore, the expression profiles of lncRNAs are considered to serve as biomarkers for cancer diagnosis [18]. Close to more than 500 temporally expressed S-phase-enriched lncRNAs have been recently identified from HeLa cells with potential prognostic value and oncogenic potential [19]. The present knowledge about diverse functions mediated by many lncRNAs, especially those implicated in gene regulation, range from them functioning as a decoy like Tsix [20], to their role in alternative splicing like metastasis-associated lung adenocarcinoma transcript 1 (MALAT1) [21], to a few acting as a scaffold like P21-associated noncoding RNA DNA damage-activated (PANDA) [22] or as a sponge like taurine up-regulated gene 1 (TUG1) [23]. A multitude of lncRNAs regulate transcriptional networks by competing for a limited pool of microRNAs and thereby function as competing endogenous RNAs (ceRNAs) like hepatocellular carcinoma up-regulated lncRNA (HULC), cAMP response element binding protein (CREB) [24], differentiation antagonizing non-protein-coding RNA (DANCR) [25], H19 [26], and many more [27]. Recently, noncoding RNAs have been identified that act as enhancer RNAs, like Bloodlinc [28], or function as allosteric modulators, like *CCND1* [29]. The varied mechanisms through which they mediate gene regulation range from chromatin remodelling by either binding to polycomb repressive complex 1/2 (PRC1/2) [30] or by forming RNA–DNA triplexes [31], to acting as competitive inhibitors of target protein/RNA interactions [31]. The lncRNAs can either directly interact with proteins or they can sequester proteins that are master regulators of cellular growth.

We here report the identification of an acutely transforming cDNA derived from the transcriptome of the mouse melanoma cells—Clone M3. This cDNA, upon transfection into mouse NIH/3T3 fibroblasts, formed foci of neoplastically transformed cells. The characterisation of the sequence of this acutely transforming gene was found to encode an lncRNA. We named this RNA as Genomic Instability-Inducing RNA (Ginir) because it exerted a regulatory role on cell proliferation and maintained genomic stability under conditions of normal cellular homeostasis. An increased expression level of Ginir in mouse fibroblast cells induced genomic instability and oncogenic transformation. Here, we provide a mechanistic insight about the oncogenic role of noncoding RNA Ginir in mouse cells. Our data exemplify that (i) Ginir RNA function is modulated by its full-length natural antisense transcript (NAT; Giniras), (ii) Ginir RNA targets centrosomal protein 112 (Cep112) and alters its subcellular localisation by binding to it, (iii) breast cancer type 1 susceptibility protein (Brca1) and Cep112 proteins interact with each other in the absence of Ginir noncoding RNA, (iv) interaction of Cep112 protein with Brca1 protein is impaired in the presence of high levels of Ginir RNA, and (v) interference of Cep112–Brca1 interaction due to increased presence of Ginir RNA causes replicative stress and induces mitotic dysregulation, causing genomic instability, and thereby propels cells towards malignant transformation.

## Results

DNA transfection assays have been successfully employed to identify the presence of putative proto-oncogenes and dominantly acting cellular oncogenes in a variety of mammalian cells, including human tumours [32,33]. We used an automatic directional cloning (ADC) approach to identify activated oncogenes [34] using Clone M3 mouse melanoma cells as a paradigm. When the expression cDNA library—prepared from the poly A+ RNA of Clone M3 mouse melanoma cells—was introduced into mouse NIH/3T3 cells by transfection, several clones of cells with morphological features of oncogenic transformation, like the one shown in S1A Fig, were generated. Sequence determination of one of the cDNA clones rescued from the transformed cells led to the identification of a 557-nucleotides-long sequence (S1B Fig).

### The 557-nucleotides-long sequence is derived from an intergenic lncRNA (lincRNA)

**Fig 1:**
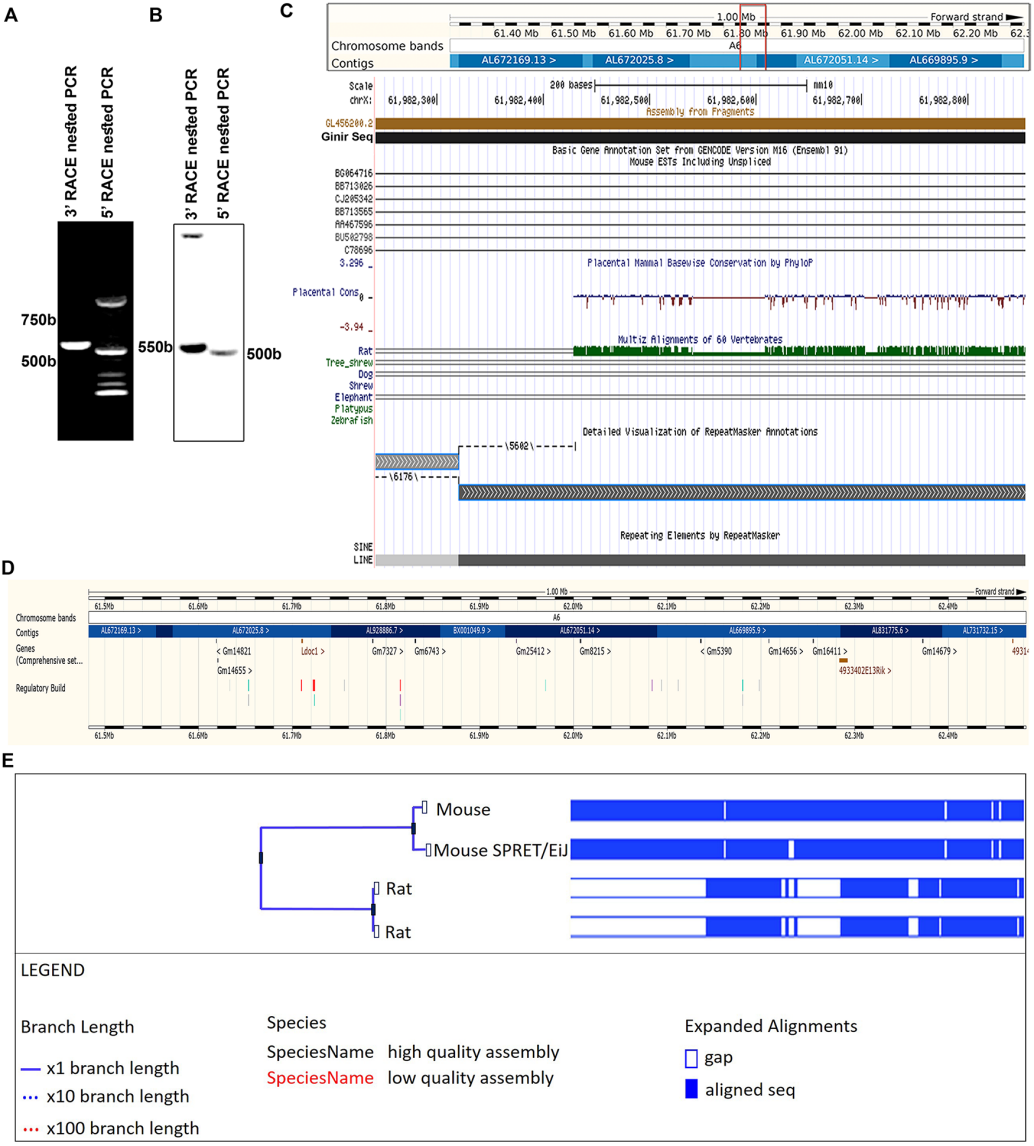
Identification and characterisation of lincRNA Ginir from mouse melanoma cells. (A and B) The 5′ and 3′ ends of the 557-base Ginir transcript were extended using RACE. PCRs were performed with AP1 and AP2 and Ginir-specific primers G1F and G4R for 3′ RACE and primers G1R and G4F for 5′ RACE (A). For nested PCR, primary PCR products were used as templates. Primer sequences are enlisted in S4 Table included in the Materials and methods section. The 5′–3′ RACE was followed by Southern hybridisation with Ginir sequence–specific probes (B). (C) Schematic representation of Ginir (NCBI Acc. No: EF649772.1) on mouse chromosome X (ChrX: 61982243–61982854) was acquired using the UCSC genome browser (http://genome.ucsc.edu/cgi-bin/hgBlat) and GRCm38/mm10 Assembly. The bottom region shows ESTs and repeat elements spanning the Ginir locus. (D) The genomic region showing location of LDOC1 and MAGE genes spanning the contig bearing Ginir (www.ensembl.org). (E) Schematic representation of Ginir genomic sequence homology between mouse and rat species acquired using ENSEMBL (www.ensembl.org). AP1, adapter primer 1; AP2, adapter primer 2; EST, expressed sequence tag; Ginir, Genomic Instability Inducing RNA; LDOC1, LDOC1, Leucine Zipper down-regulated in cancer-1; lincRNA, long intergenic noncoding RNA; MAGE, the melanoma antigen gene; NCBI, National Center for Biotechnology Information; PCR, polymerase chain reaction; RACE, rapid amplification of cDNA ends; UCSC, University of California, Santa Cruz.

This transforming cDNA sequence was unconventional because it encoded three putative ORFs, each with a short sequence of amino acids (aa) (ORF1–46 aa, ORF2–51 aa, and ORF3–104 aa) (S1C Fig). Of these three ORFs, only ORF1 and ORF2 were expressed as an N-terminal green fluorescent protein (GFP) fusion protein of sizes 38.1 and 39.1 kDa, respectively (S1D Fig). However, the ectopic expression of each these two putative proteins neither yielded any discernible phenotype nor showed any oncogenic transformation *in vitro* or *in vivo*, as seen by the tumourigenicity assay in immune-compromised mice (S1E Fig). Moreover, the expression of the sequence of 557 bases as a fusion RNA appended either to the 5′ or 3′ UTR of GFP was highly tumourigenic (S1E Fig). These results together ruled out the possibility that the two distinct ORFs—ORF1 and ORF2—could specify oncoproteins. Instead, it suggested that the sequence of 557 nucleotides itself was functioning as a noncoding oncogenic RNA. The noncoding nature of this sequence was confirmed using software tools like Coding Potential Assessment Tool (CPAT) (S1F Fig) and Coding Potential Calculator (CPC2) (S1G Fig). The sequence of this transcript was extended using 5′ and 3′ rapid amplification of cDNA ends (RACE) in both directions to obtain a 612-nucleotides-long extended sequence (Fig 1A and 1B). The 612-base-sequence data deposited at the National Center for Biotechnology Information (NCBI) bear the accession number EF649772.1. The ectopic expression of 612 nucleotides’ RNA in NIH/3T3 cells also caused oncogenic transformation like its 557-nucleotides-long counterpart. BLAST analyses indicated that the 612-nucleotides-long transcript sequence originated from an intronless genomic segment located at position A6q of the X chromosome (Fig 1C), and it was flanked by the genes coding for the proteins Leucine Zipper down-regulated in cancer-1 (LDOC1) and the melanoma-associated antigen-11 on its 5′ and 3′ ends respectively (Fig 1D). Its coordinates on mouse X chromosome were chrX:61,982,243–61,982,854 (https://genome.ucsc.edu; GRCm38/mm10 Assembly). The sequence was found significantly conserved in the rat genome (Fig 1C and 1E).

Because the 612-nucleotides-long RNA originated from an independent transcription unit having no overlap with these two adjacent protein-coding genes, its identity became obvious as lincRNA. Bioinformatic analyses of Ginir sequence demonstrated its part homology to several unannotated noncoding RNAs (S2A Fig). More significantly, a deregulated overexpression of this lincRNA resulted in genomic instability (see the section Ginir RNA expression in cells induces dsDNA breaks and activates DNA damage response (DDR) pathway proteins) accompanied by oncogenic transformation, demonstrating that it was functioning as a Ginir.

### Ginir/Giniras pair is transcribed in normal cells and during embryonic development

**Fig 2:**
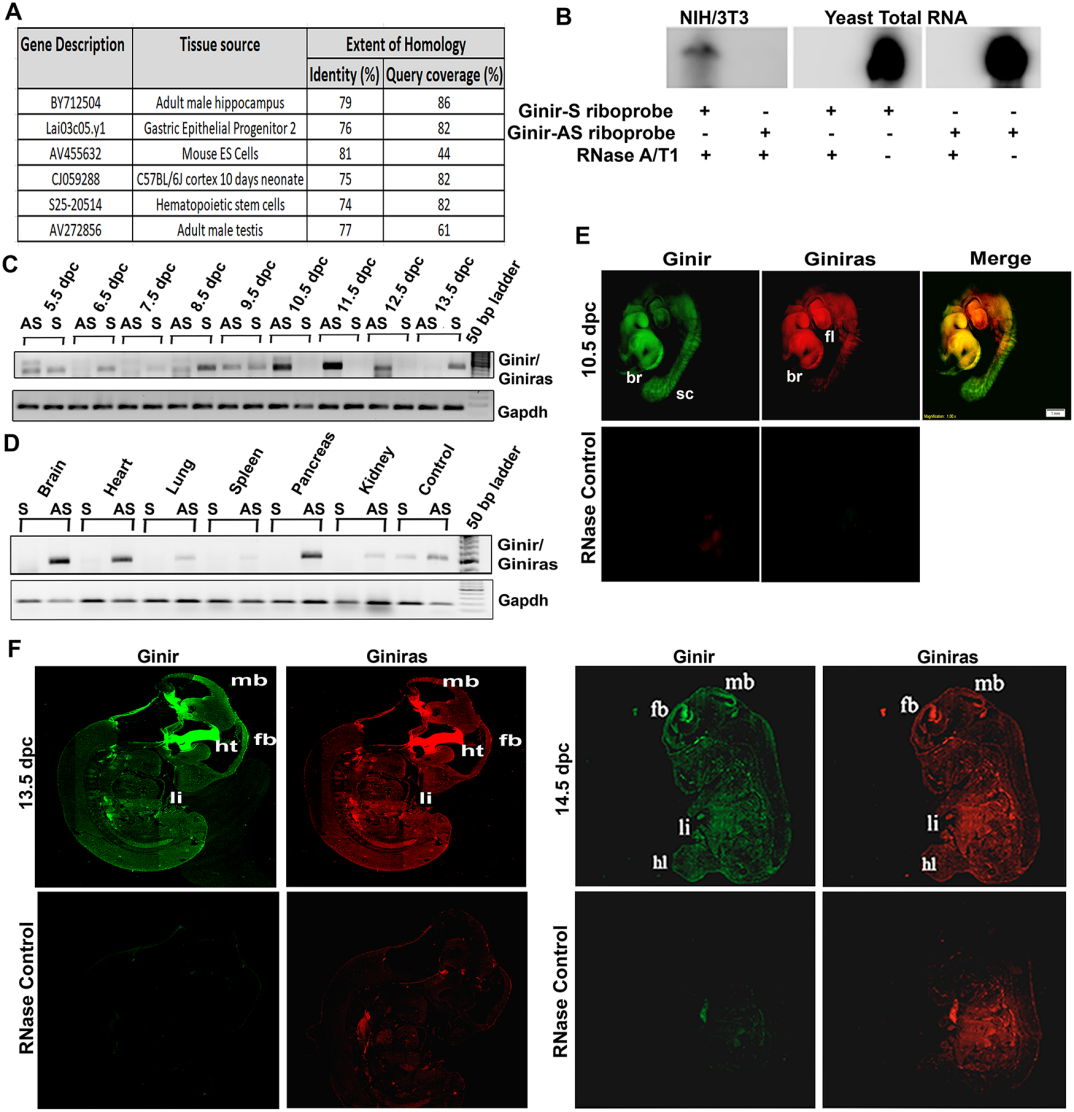
Expression of Ginir/Giniras transcripts during mouse embryonic development and in adult tissues. (A) List of several ESTs spanning Ginir sequence and showing significant similarity to Ginir (http://blast.ncbi.nlm.nih.gov/Blast.cgi). (B) RPA of RNA isolated from NIH/3T3 cells using PCR-generated sense or antisense riboprobes specific to Ginir sequence. Yeast total RNA served as control for RNase A/T1 activity. (C and D) Expression of Ginir/Giniras RNA in different stages of development (dpc) of mouse embryos (C) and in tissues from different organs of adult mice (D) using strand-specific cDNA synthesis and RT-PCR with G1F-G1R primers. Gapdh served as internal loading control. (E) Whole-mount ISH using LNA probes for Ginir (FAM labelled, green) or Giniras (TexRed labelled, red) on 10.5 dpc mouse embryos showing differential expression in brain (‘br’), forelimbs (‘fl’), and spinal cord (‘sc’). Whole-mount embryos treated with RNase A served as negative control for fluorescence. (F) FISH using LNA probes for Ginir (Green) or Giniras (Red) on embryo sections of 13.5 and 14.5 dpc embryos showing differential expression in forebrain (‘fb’), midbrain (‘mb’), hypothalamus (‘ht’), and limbs (‘li’). Embryo sections treated with RNase A served as a negative control. dpc, days post coitum; EST, expressed sequence tag; FAM, fluorescein amidite; FISH, fluorescence in situ hybridisation; Gapdh, glyceride 3-phosphate dehydrogenase; Ginir, Genomic Instability Inducing RNA; Giniras, antisense RNA of Ginir; ISH, in situ hybridisation; LNA, locked nucleic acid; PCR, polymerase chain reaction; RPA, ribonuclease protection assay; RT-PCR, reverse transcription PCR.

Homology search data to Ginir sequence (http://www.ensembl.org/; http://blast.ncbi.nlm.nih.gov) demonstrated that the Ginir sequence was partially homologous to several expressed sequence tags (ESTs) apparently relevant to neurogenesis and especially to those that were specifically found during embryogenesis (Figs 1C and 2A). This prompted us to examine if Ginir RNA was being expressed in cultured cells of embryonic origin like in NIH/3T3 cells and was also expressed during mouse embryonic development. When RNase protection assay (RPA) using RNA isolated from NIH/3T3 cells was performed using Ginir-specific sense and antisense hybridisation riboprobes, we found a completely overlapping antisense RNA to Ginir (Giniras) being transcribed (Fig 2B). Further, employing an orientation-specific polymerase chain reaction (PCR) amplification approach in which strand-specific primers for cDNA synthesis were used, we yet again obtained a predominant expression of the Giniras transcript in NIH/3T3 cells (S2C Fig). In contrast, a weaker expression of Ginir RNA was evident under similar culture conditions in NIH/3T3 cells by reverse transcription polymerase chain reaction (RT-PCR) (S2C Fig) as well as by RPA (Fig 2B). Though not well represented, the RNA sequencing (RNA-seq) analysis of total RNA from NIH/3T3 cells generated sequence reads that matched the genomic sequence of Ginir region on the X chromosome (S1 Table, S2D Fig).

The poor representation of lncRNA sequences in the RNA-seq data is reported for several lincRNAs, and this is often ascribed to their low expression levels, spatiotemporal expression pattern, and near-universal alternative splicing of noncoding exons [35]. With the finding that the Ginir RNA sequence predominately displays homology to several ESTs that are expressed during mouse embryonic development, it is possible that Ginir RNA expression might also have a characteristic spatiotemporal expression profile as seen with many other lncRNAs [36,37]. We therefore isolated RNA from developmentally timed whole mouse embryos and determined the expression of both Ginir and Giniras transcripts in them. The Ginir transcript was detectable during the embryonic developmental stages of 5.5 to 9.5 days and again at 13.5 days post coitum (dpc). By contrast, the Giniras transcript was clearly present in the intervals of 10.5 to 12.5 dpc (Fig 2C). Instead, a more prominent Giniras RNA expression relative to Ginir RNA expression was evident in some of the adult mouse tissues examined, which included brain, heart, lungs, spleen, pancreas, and kidney (Fig 2D). These findings are consistent with an argument that Ginir RNA expression is more tightly coupled to proliferative stages of cells during embryonic development, whereas predominant Giniras RNA expression is associated with nonproliferative cells of the organs. When fluorescein amidite (FAM)-labelled Ginir-specific locked nucleic acid (LNA) probe (FAM-LNA-Ginir) and Texas Red–labelled Giniras-specific LNA probes (TxR-LNA-Giniras) were used on the whole embryos individually or together, it became clear that expression of Ginir, as well as Giniras RNA, was differentially regulated in various differentiating tissues during embryonic development. In 10.5-, 13.5-, and 14.5-dpc mouse embryos (Fig 2E and 2F), the presence of both Ginir and Giniras transcripts was detectable in appreciable amounts, particularly in the developing ventricles of the brain and forelimb, signifying that both these transcripts may have functions in the development of these tissues. Whereas Giniras transcript was found exclusively localised to emerging forelimb buds by stage 10.5 dpc, Ginir RNA was differentially localised to the spinal cord (Fig 2E). By stage 13.5 dpc, a higher abundance of both Ginir and Giniras transcripts was marked in both brain and developing forelimb buds (Fig 2F); this colocalised expression pattern of Ginir and Giniras RNA was restricted to specific regions of the forebrain and midbrain and persisted until 14.5 dpc (Fig 2F). Taken together, these findings demonstrated that Ginir was specifically expressed during embryonic development of brain, spinal cord, and forelimb buds at early to midgestational stages, whereas the Giniras expression followed temporally to Ginir function in developing tissues and significantly was more prominent in several adult tissues. These observations are in accord with reports that the expression of lncRNAs are spatially precise and are often restricted to particular structures or cell types of the brain [38]. It is well established that the brain is a complex organ and harbours the most transcriptional diversity amongst other somatic tissues [35].

### Overexpression of Ginir RNA in cultured cells induces oncogenic transformation

**Fig 3:**
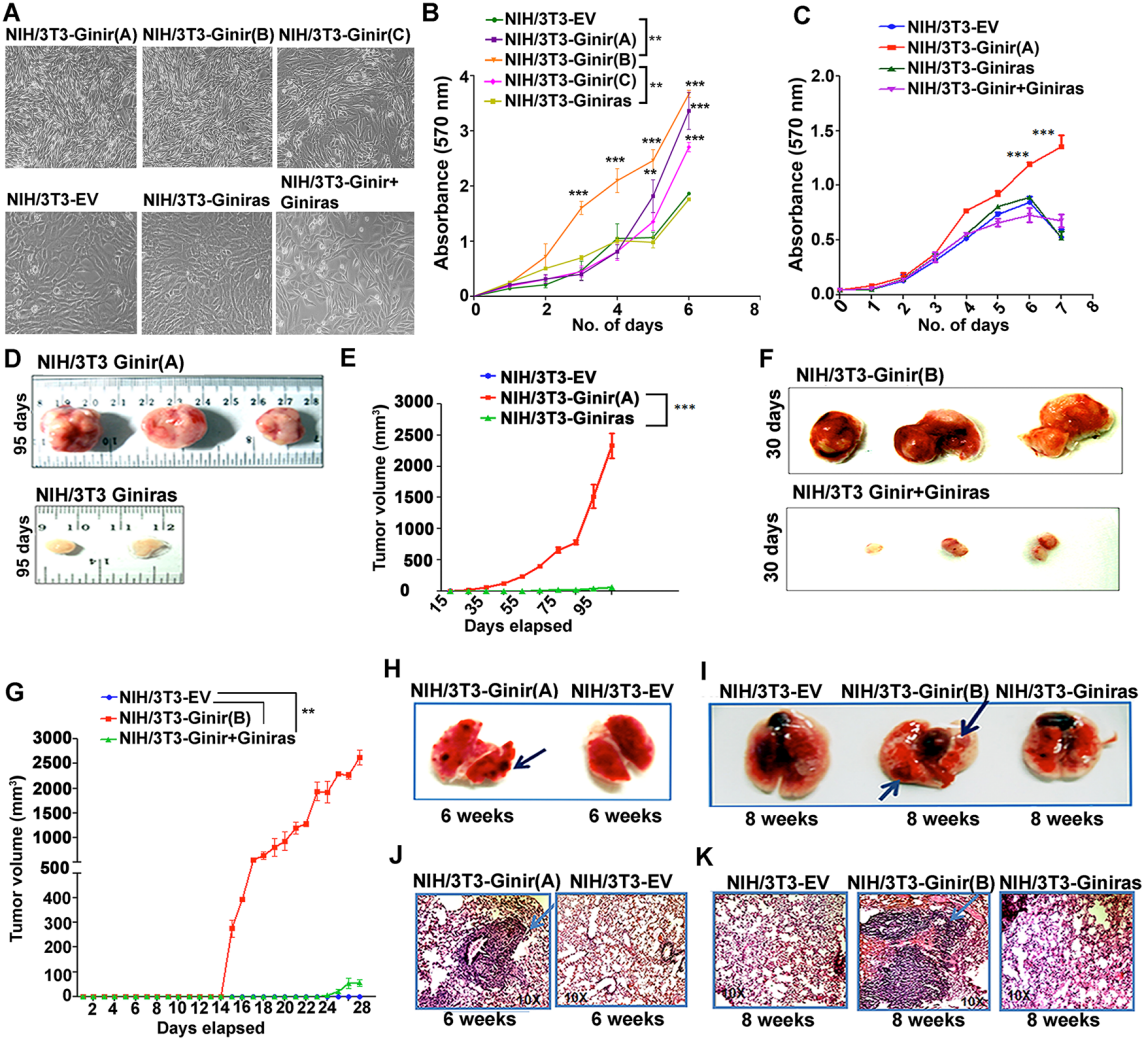
Ectopic expression of Ginir RNA in cells leads to malignant transformation. (A) Phase contrast micrographs of NIH/3T3-Ginir (three independent transfectants A, B, and C), NIH/3T3-EV, NIH/3T3-Giniras, and NIH/3T3-Ginir+Giniras cells. (Magnification 4×). (B) Cell proliferation analyses of NIH/3T3-Ginir (A, B, and C) individual transfectants, NIH/3T3-Giniras, and NIH/3T3-EV transfectant cells performed by MTT assay over a period of 6 days. Values are mean ± SEM (*n* = 3); **P ≤ 0.001 by repeated-measures one-way ANOVA test. (C) Cell proliferation analyses of NIH/3T3-EV, NIH/3T3-Ginir(A), NIH/3T3-Giniras, and NIH/3T3-Ginir+Giniras cells performed by MTT assay over a period of 7 days. Values are mean ± SEM (*n* = 3); ***P ≤ 0.0001; by repeated-measures one-way ANOVA test. (D) Representative xenograft tumours of NIH/3T3-Ginir(A) and NIH/3T3-Giniras cells generated in NOD/SCID mice. Tumour growth was monitored for 95 days. Tumourigenicity assays were performed at least 5 times with 3 mice in each group. (E) Tumour growth kinetics determined by measuring tumour volumes at an interval of 10 days over a period of 95 days. Values represent means ± SEM. ***P ≤ 0.0001, one tailed, by two-way ANOVA test. (F) Representative xenograft tumours of NIH/3T3-Ginir(B) and NIH/3T3-Ginir+Giniras cells generated in NOD/SCID mice. Tumour growth was monitored for 30 days (*n* = 3). Tumourigenicity assays were performed at least 3 times with 3 mice in each group. (G) Tumour growth kinetics monitored by measuring tumour volumes periodically up to duration of 30 days. Values represent mean ± SEM. ***P ≤ 0.0001, one tailed, by two-way ANOVA test. (H and I). In vivo lung colonisation assay in NOD/SCID mice performed via tail vein injection of NIH/3T3-Ginir(A) (H) and NIH/3T3-Ginir(B) (I) cells along with NIH/3T3-EV and NIH/3T3-Giniras cells as controls. Images represent lung tissues dissected from mice injected with mentioned cell lines after 6 (H) and 8 weeks (I) of introduction of cells. The assays were performed at least 3–5 times with *n* = 3 mice. (J and K) HE staining of lung tissue from mice injected with NIH/3T3-Ginir(A) (J) and NIH/3T3-Ginir(B) (K) cells. Supporting data for B, C, E and G can be found in S1 Data. Ginir, Genomic Instability Inducing RNA; Giniras, antisense RNA of Ginir; HE, haematoxylin–eosin; MTT, 3-(4, 5-dimethylthiazol-2-yl)-2, 5-diphenyltetrazolium bromide.

To address the function of the individual transcripts of the noncoding RNA pair Ginir and Giniras, we took advantage of our prior observations (S1A Fig) that had demonstrated the foci-forming potential of Ginir RNA upon its ectopic expression in NIH/3T3 cells. To gain a mechanistic insight into their role in cell transformation, we generated stable cell lines of NIH/3T3 cells that overexpressed either the individual transcripts of Ginir or Giniras or both in combination (Ginir+Giniras) from the strong cytomegalovirus (CMV) promoter. The clone of cells stably expressing Ginir RNA (NIH/3T3-Ginir) had 16-fold-higher levels (*P* ≤ 0.0001) of Ginir transcript over endogenous level of expression in NIH/3T3 cells (S3A Fig). Similarly, the stable clone of cells expressing Giniras RNA (NIH/3T3-Giniras) had 12-fold-higher amounts of Giniras transcript than its endogenous level of expression in NIH/3T3 cells (*P* ≤ 0.001). The RPA performed using NIH/3T3-Ginir cells confirmed overexpression of Ginir transcript as compared to Giniras (S3B Fig). We performed multiple transfections (*n* = 5) to generate several independent stable cell lines of NIH/3T3-Ginir, NIH/3T3-Giniras, and NIH/3T3-Ginir+Giniras overexpressing either transcript or both. For comparison, clones were generated with only the empty vector (NIH/3T3-EV), which served as the control. A few of the multiple independent clones of NIH/3T3-Ginir (termed here as clones A, B, and C) were randomly picked up for further studies. The expanded population of cells from these individual clones shared the following properties: they (i) appeared highly refractile, (ii) had lost contact inhibition (Fig 3A), (iii) had a higher proliferative potential as measured by 3-(4, 5-dimethylthiazol-2-yl)-2, 5-diphenyltetrazolium bromide (MTT) assay (Fig 3B), and (iv) showed a higher percent of positivity for the proliferation marker Ki67 as compared to the cells derived from NIH/3T3-EV, NIH/3T3-Giniras, or NIH/3T3-Ginir+Giniras clones (S3C Fig). The NIH/3T3-Ginir+Giniras transfectant clones showed proliferative potential comparable to NIH/3T3-EV cells (Fig 3C). We picked the expanded cells of NIH/3T3-Ginir (Clone A) for further analysis *in vitro*. Fluorescence-activated cell sorting (FACS) analysis of propidium iodide (PI)-stained cells of NIH/3T3-Ginir (Clone A) demonstrated an increased fraction of cycling cells (S3D and S3E Fig) as compared to NIH/3T3-EV cells. Clone A transfectant cells expressed higher levels of phosphorylated retinoblastoma protein (pRb), had increased levels of cyclins D1 and E (S3F Fig), showed increased clonogenicity in soft agar (S3G Fig), and displayed higher invasion potential in a Matrigel assay (S3H Fig). The NIH/3T3-Ginir cells also demonstrated an increased migration rate in wound healing assay (S3I and S3J Fig) and exhibited a pronounced angiogenic potential in a chicken chorioallantoic membrane (CAM) experimental system (S3K Fig). Subcutaneous introduction of NIH/3T3-Ginir(A) cells rapidly formed malignant tumours in NOD/SCID mice at the injected sites within 2 weeks of injections (Fig 3D–3G), whereas mice injected with NIH/3T3-EV or NIH/3T3-Giniras cells did not develop tumours up to 95 days post injection (Fig 3D and 3E). When the tumour forming potential of NIH/3T3-Ginir+Giniras cells was examined, they formed tumours of substantially reduced sizes (*P* < 0.0001) and with a delayed onset (Fig 3F and 3G). Kaplan-Meir survival analysis of NIH/3T3-Ginir-induced tumour-bearing mice demonstrated a median survival of 75 days (S3L Fig), whereas mice injected with NIH/3T3-EV and NIH/3T3-Giniras cells survived healthily for prolonged periods (>95 days; *P* ≤ 0.0001). Collectively, these results indicate that Ginir functions as a dominant oncogene in mouse cells.

### NIH/3T3-Ginir cells efficiently colonise lung tissue in a tail vein assay

A number of noncoding RNAs such as urothelial cancer associated 1 (UCA1), MALAT1, H19, and plasmacytoma variant translocation (PVT1) have been implicated in the promotion of metastasis of tumour cells, but evidence for a more direct role of these RNAs in this process is still lacking [39]. Our findings that the NIH/3T3-Ginir cells had an enhanced migration potential in both 2D and 3D matrix assays in vitro led us to investigate whether Ginir RNA–induced tumour cells would also have detectable metastatic potential in a lung colonisation assay. To assay metastasis, we performed a standard tail vein assay [40]. For this, we introduced cells grown out from NIH/3T3-Ginir (Clones A and B) and NIH/3T3-Giniras cells through the tail veins of NOD/SCID mice. In addition, we also examined if the same cells could metastasise or colonise to any distant tissue when introduced through subcutaneous injections. These experiments demonstrated that NIH/3T3-Ginir cells generated numerous macroscopic foci of tumour cells in the lungs of the injected mice (Fig 3H and 3I) within 6–8 weeks post injection of cells. This was also confirmed by the haematoxylin–eosin (HE) staining of the lung tissues of NIH/3T3-Ginir cells (Fig 3J and 3K). These data contrasted with the results obtained from mice injected with similar numbers of NIH/3T3-EV or NIH/3T3-Giniras cells, which did not colonise tumour foci to lungs or any other tissues (Fig 3H and 3I) up to 12 weeks. When NIH/3T3-Ginir cells (from Clones A and B) were introduced subcutaneously, not only did tumours develop at the injected site but also the colonisation of lungs in the injected mice could be seen by 11 weeks (S3M Fig), which was also confirmed by HE staining of the lung tissues (S3N Fig). These data demonstrated the potential of Ginir RNA to act as a metastasis-promoting noncoding RNA. In summary, our data establish that lncRNA Ginir functions as a metastasis-inducing oncogenic RNA.

To uncover differential gene expression, if any, we performed transcriptome sequencing and compared the RNA profiles of NIH/3T3, NIH/3T3-Ginir(A), NIH/3T3-Giniras, and Clone M3 cell lines. High-quality sequence reads were obtained with all the samples as shown in S4A and S4B Fig, and a heatmap generated from the expression data is shown in S4C Fig. The differential expression of RNA from NIH/3T3-EV cells in comparison with NIH/3T3-Ginir(A) cells indicated that 555 genes were significantly (*P* ≤ 0.05) altered (S4D and S4E Fig). Gene Ontology (GO) analysis of the gene data sets and heatmap demonstrated that the enrichment terms were mainly concentrated to pathways important for cell cycle progression, cell division, RNA splicing, RNA processing, and cell–cell adhesion (S4F and S4G Fig). An analysis of the cellular components involved in these processes indicated that most of these genes were localised to the centromeric regions of the chromosomes, the kinetochores, and the cell–cell adherens junctions (S4H and S4I Fig). These RNA-seq differential expression data suggested the role of Ginir in cell division and mitosis.

### Continuous expression of Ginir RNA is required for its oncogenic function

**Fig 4:**
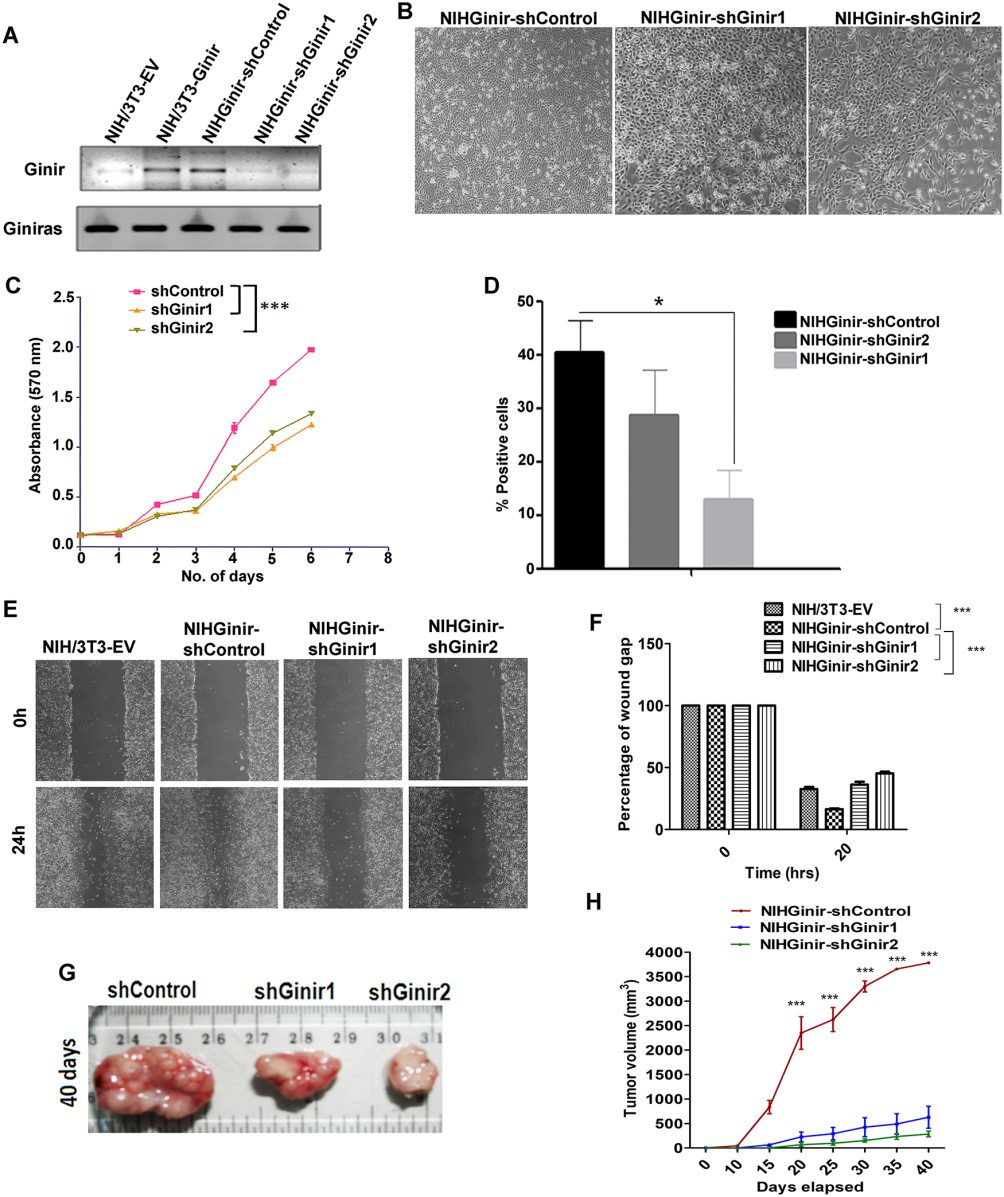
Down-regulation of Ginir RNA levels attenuates tumourigenic potential of NIH/3T3-Ginir cells. (A) Representative RT-PCR analysis demonstrating Ginir levels in NIH/3T3-EV and NIH/3T3-Ginir(A) cells expressing Ginir-shRNA 1 and 2 and shControl (Scrambled) using G2F-G2R primers. Gapdh served as an internal RNA control. (B) Representative-phase contrast images of NIHGinir cells stably transfected with shRNAs to Ginir (shGinir1 or shGinir2) or with a scrambled shRNA (shControl). (Magnification 10×). (C) Cell proliferation analysis using MTT assay for the indicated cell lines. Data are mean ± SEM. ***P ≤ 0.0001, one tailed, paired Student’s *t* test. (D) Quantification of Ki67 antigen expression shown as percentage of positively stained cells compared to total number of cells per field (number of fields counted were at least 10) in indicated cell lines. Values are mean ± SEM. **P ≤ 0.001. Two tailed, by one-way ANOVA test. (E and F). Wound healing assays performed with NIH/3T3, NIHGinir-shControl, and NIHGinir-shGinir (1 and 2) cells. The wound gaps were observed at two different time points: 0 hours and 24 hours post wound formation (*n* = 3) (E). Statistical analysis of wound closure in mentioned cell types wherein the percentage of wound gap measured at indicated time points is plotted and the values are represented as mean ± SEM; ***P < 0.0001 by regular two-way ANOVA test (F). (G) Representative photographs of tumours demonstrating the inhibitory effects of Ginir-shRNA on tumour growth potential of NIH/3T3-Ginir(A) cells. Xenograft growth was monitored in NOD/SCID mice at regular intervals of 5 days for up to 40 days. Later, mice were killed, and tumours were dissected out. The assay was performed at least 3 times (*n* = 3). (H) Tumour growth kinetics of the mentioned cell lines monitored over a period of 40 days. Data are mean ± SEM; ***P value ≤ 0.0001 by regular two-way ANOVA test. Supporting data for C, D, F, and H can be found in S2 Data. Gapdh, glyceride 3-phosphate dehydrogenase; MTT, 3-(4, 5-dimethylthiazol-2-yl)-2, 5-diphenyltetrazolium bromide; RT-PCR, reverse transcription polymerase chain reaction; shRNA, short hairpin RNA.

Next, we asked if a high level of Ginir expression was continuously required for the induction and/or maintenance of the transformed state or whether it involved a hit-and-run mechanism. To find this, we down-regulated Ginir expression in NIH-Ginir(A) and NIH-Ginir(B) cells by superimposing the expression of Ginir-specific short hairpin RNAs (shRNAs). The shRNAs were designed to target specifically two different regions of Ginir RNA sequence (sequences as specified in Materials and methods) and were termed as shGinir1 and shGinir2. Stable cell line clones expressing these shRNAs were generated, and expanded cells from them were used for further studies. We found that the Ginir RNA expression level had significantly decreased with shGinir1 and shGinir2 knock-down constructs in NIH/3T3-Ginir cells (Fig 4A). The Ginir RNA–deficient NIH/3T3-Ginir(A) cells displayed a reversal of their refractive cell morphology and appeared more like NIH/3T3 cells (Fig 4B and S5A Fig). In addition, the same cells had a reduced proliferative potential in an MTT assay (Fig 4C), a lower fraction of cycling cells in the S+G2/M phase (S5C Fig), a decreased percent of positivity for Ki67 (Fig 4D and S5B Fig), and an impaired 2D cell migration potential in culture (Fig 4E). Importantly with shRNA-mediated Ginir RNA depletion, the cells formed smaller-sized tumours as compared to the parent NIH/3T3-Ginir(A) cells in the tumourigenicity assays done in NOD/SCID mice (*P ≤* 0.0001) (Fig 4G and 4H). The cells derived from the NIH/3T3-Ginir(B) clone also showed similar phenotypes and reduced tumourigenicity after the Ginir RNA levels were independently knocked down by introducing Ginir shRNAs 1 and 2 into them (S5D Fig). Thus, when Ginir RNA was down-regulated, it caused significant attenuation of tumour phenotype, indicating that a high level of Ginir RNA expression was continuously required for the maintenance of malignant transformation. Taken together, these data provide unambiguous evidence about the oncogenic nature of Ginir.

### Knock-down of endogenous Ginir inhibits proliferation

**Fig 5:**
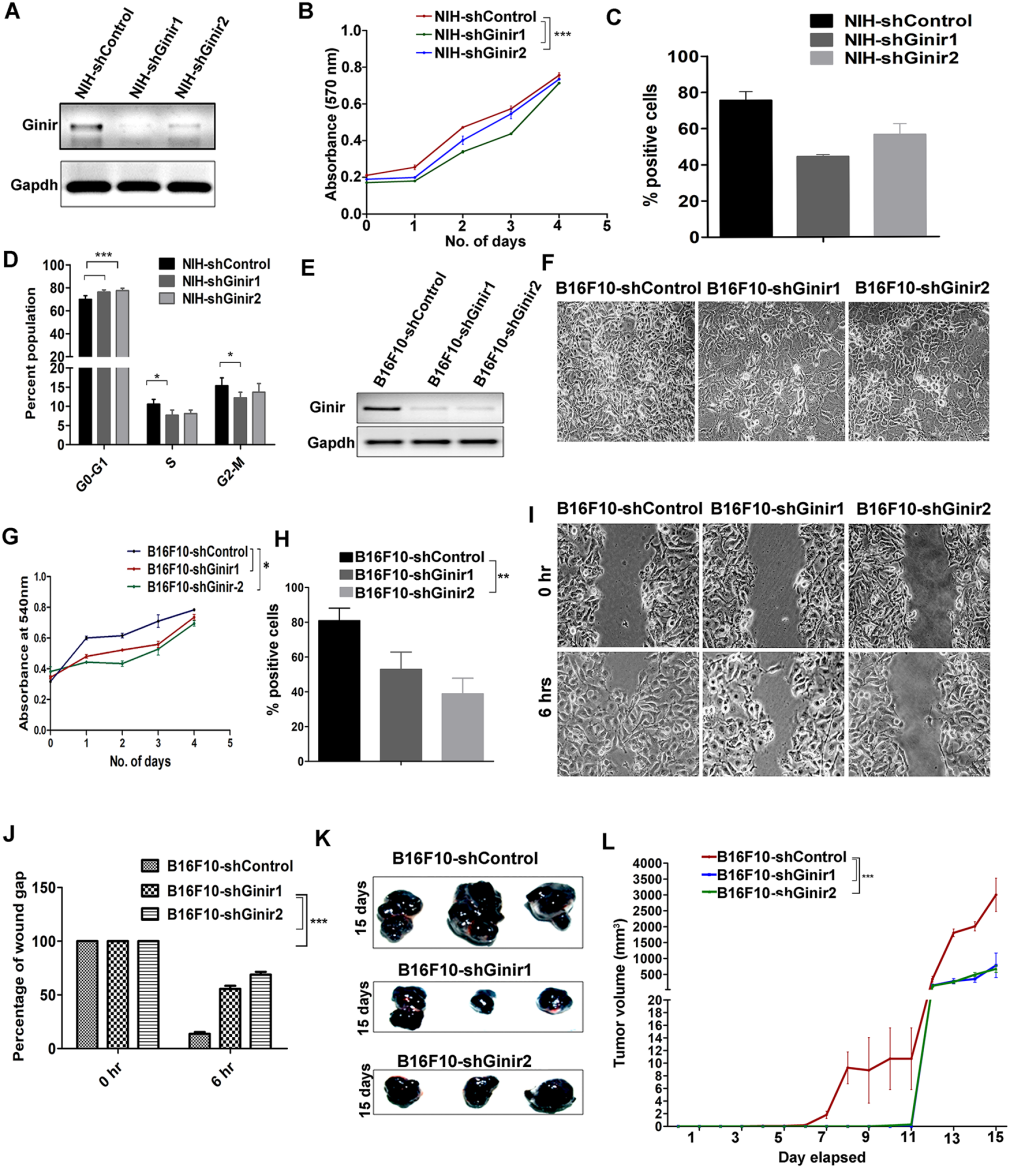
Knock-down of endogenous Ginir affects proliferation and tumourigenic potential of fibroblasts and melanoma cells. (A) Representative RT-PCR for analyses of Ginir knock-down in NIH/3T3 cells expressing Ginir-shRNA (1, 2, and scrambled control) using G2F-G2R primers. Gapdh served as an internal control. (B) Cell proliferation analysis by MTT assay for the indicated cell lines performed over a period of 4 days. Data presented are mean ± SEM. ***P ≤ 0.0001, one tailed, by two-way ANOVA test. (C) Quantification of Ki67 antigen–expressing cells shown as percentage of positively stained cells from the total number of cells present per field. Cells from at least 10 representative fields were counted in NIH/3T3 control and Ginir shRNA1 and 2 cells. Data presented are mean ± SEM.**P ≤ 0.001 by one-way ANOVA test (*n* = 3). (D) Quantitative analyses of cell cycle parameters of PI-stained cells in flow cytometry. Stable Ginir knock-down (NIHshGinir1, NIHshGinir2) cells were compared to NIH/3T3 control cells having endogenous Ginir RNA expression for percentage of cells in various phases of the cell cycle. Bars: means ± SEM; *P ≤ 0.05, **P ≤ 0.001; two tailed; by two-way ANOVA test. (E) Representative RT-PCR data demonstrating Ginir RNA expression levels in B16F10 melanoma cells shControl and the knock-down cells B16F10-Ginir-shRNA1 and B16F10-GinirshRNA2. PCR was done using G2F-G2R primers. Gapdh RNA expression was used for normalisation. (F) Phase contrast images of B16F10 control cells and cells stably transfected with shGinir1 or shGinir2. Arrowheads point towards cells showing altered phenotypes. Magnification 10×. (G) Cell proliferation analysis of indicated cells performed by MTT assay over a period of 4 days. Data shown are mean ± SEM. *P ≤ 0.05, one tailed, Student’s paired *t* test (*n* = 3). (H) Quantification of Ki67 antigen expression shown as percentage of positively stained cells as compared to the total number of cells per field. At least 10 fields were counted. Data shown are mean ± SEM. **P ≤ 0.001 by one-way ANOVA test. (*n* = 3). (I) Cell migration assay in the indicated cell lines. The gaps were measured after 6 hours using ImageJ software; version 1.41. Experiment was repeated at least thrice. (J) Quantitative analysis of relative wound recovery of each B16F10 Ginir knock-down cell induced by shRNA1 and shRNA2 as compared to B16F10-shGinir scrambled expressing control cells. Data represent mean ± SEM (*n* = 3). ***P ≤ 0.0001 by two-way ANOVA test. (K) Representative xenograft tumours of B16F10-shControl and B16F10-shGinir1 and B16F10shGinir2 (1 and 2) cells introduced into NOD/SCID mice; tumour growth was monitored for 15 days. These experiments were performed thrice, with 3 mice in each group. (L) Tumour growth kinetics were determined by measuring tumour volume each day for a period of 15 days for the indicated cell lines. Data shown represent mean ± SEM. ***P ≤ 0.0001, one tailed, one-way ANOVA test. Supporting data for B, C, D, G, H, and L can be found in S3 Data. Gapdh, glyceride 3-phosphate dehydrogenase; Ginir, Genomic Instability Inducing RNA; MTT, 3-(4, 5-dimethylthiazol-2-yl)-2, 5-diphenyltetrazolium bromide; PCR, polymerase chain reaction; PI, propidium iodide; RT-PCR, reverse transcription PCR; shRNA, short hairpin RNA.

To address the role of endogenous Ginir RNA, we used two mouse cell lines; one was NIH/3T3, and the other was B16F10 melanoma cell line. As stated previously, NIH/3T3 cells have basal levels of Ginir RNA expression, whereas B16F10 melanoma cells express high levels of Ginir RNA. We generated Ginir-deficient cell lines of both of them by expressing shRNA1 and shRNA2 in them. The down-regulation of Ginir RNA in NIH-shRNA1 and NIH-shRNA2 cells was confirmed by RT-PCR (Fig 5A). The NIH-shRNA Ginir1 and NIH-shRNA Ginir2 cells demonstrated a lower proliferative rate in MTT assay (*P* ≤ 0.0001) (Fig 5B) and had decreased levels of Ki67 antigen expression (*P* ≤ 0.05) as compared to control cells generated by introducing a scrambled shRNA (NIH-shRNA Control) (Fig 5C and S6A Fig). Down-regulation of Ginir RNA altered the cell cycle kinetics by decreasing the population of cycling cells in the S+G2/M phase and increasing the proportion of cells in the G0/G1 phase (Fig 5D). These data in combination with the Ginir RNA overexpression data cited earlier provide evidence for a role of Ginir RNA in maintaining homeostasis in the cell proliferation dynamics. Particularly in NIH/3T3 cells, moderate Ginir RNA expression positively regulated cell proliferation, and its reduced level was favourable for slow cycling, whereas its overexpression perpetuated unregulated growth and cell transformation.

We also generated Ginir-deficient B16F10 melanoma cells by the same procedure followed for NIH/3T3 cells and confirmed the down-regulation of Ginir RNA in these cells by RT-PCR (Fig 5E). We used the Ginir RNA–deficient B16F10 cells to compare the growth and transforming potential of these cells with their parental B16F10 cells. The Ginir-deficient B16F10 cells exhibited a more differentiated morphology (Fig 5F), showed reduced proliferative potential in MTT assay (Fig 5G), possessed a lower fraction of cells expressing KI67 antigen (Fig 5H and S6B Fig), and demonstrated decreased cell migration in wound healing assay (Fig 5I and 5J). B16F10 cells are highly tumourigenic and metastatic in nature. Consistent with our tumour data obtained with NIH/3T3-Ginir cells and their NIH-GinirshRNA derivative cells displaying contrasting growth kinetics, we found that these experiments with B16F10 melanoma cells showed similar retardation of tumour growth (Fig 5K and S6C Fig). The reduced tumour size could result from a delayed initiation of tumour growth (S6C Fig) or could be an outcome of a decreased tumour volume due to an overall reduction in the tumour growth kinetics (Fig 5L). Ginir knock-down in B16F10 cells affected melanin production, resulting in reduced pigmentation (S6D Fig) as compared to B16F10 cells. These data make us speculate that besides proliferation, Ginir RNA may also be involved in pigmentation.

### More Ginir RNA is partitioned into the nucleus in the NIH-Ginir-transformed cells

**Fig 6:**
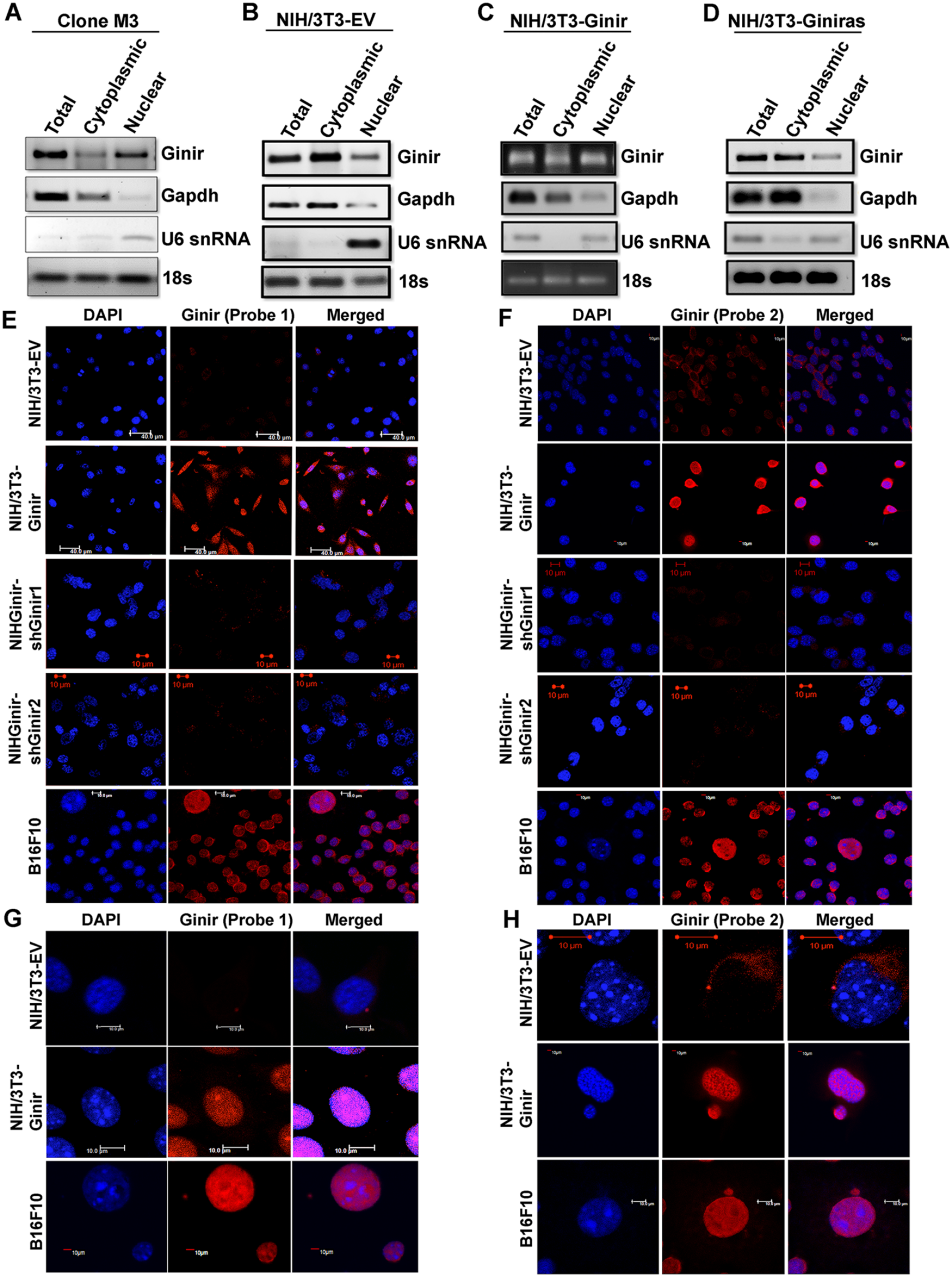
Ginir RNA is localised to cytoplasm in normal cells, but partitioning switches to the nuclear compartments in transformed cells. (A-D) Subcellular fractionation followed by RT-PCR using G1F-G1R primers to determine compartmentalisation of Ginir and Giniras RNAs in (A) Clone M3, (B) NIH/3T3-EV, (C) NIH/3T3-Ginir, and (D) NIH/3T3-Giniras cells. Gapdh RNA served as an internal control for cytoplasmic RNA fractions and U6 snRNA as control for the nuclear fractions. The 18S rRNA served as a loading control for RT-PCR. A total of 35 cycles of amplifications were used for Ginir and Giniras RNAs, and 30 cycles were used for Gapdh RNA, U6 snRNA, and 18S rRNA. (E-H) RNA-FISH for Ginir localisation in the indicated cell lines using two independent LNA Ginir probes (Probe1-FAM labelled [E, G] and Probe2-Texas Red labelled [F, H]). Magnified images of a few cells are represented in G and H. Blue colour indicates nuclear staining with DAPI. Scale bar 10 μm. Gapdh, glyceride 3-phosphate dehydrogenase; Ginir, Genomic Instability Inducing RNA; Giniras, antisense RNA to Ginir; LNA, locked nucleic acid; RNA-FISH, RNA fluorescence in situ hybridisation; RT-PCR, reverse transcription polymerase chain reaction; snRNA, small nuclear RNA.

To understand the function of Ginir RNA in cell transformation, it is important to know its subcellular localisation within the cells. To gain insight into this, we obtained RNA from the nuclear and cytoplasmic compartments of Clone M3, NIH/3T3-EV, NIH/3T3-Ginir, and NIH/3T3-Giniras cells. By using strand-specific primers for cDNA synthesis followed by RT-PCR, we measured Ginir and Giniras RNA levels in these two compartments. The Ginir RNA was found to be more abundant in the nuclear compartment of Clone M3 (Fig 6A) and NIH/3T3-Ginir cells (Fig 6C), whereas it was enriched in the cytoplasm of NIH/3T3-EV (Fig 6B) and NIH/3T3-Giniras cells (Fig 6D) Furthermore, by in situ hybridisation using two independent Ginir sequence–specific LNA probes, we observed a more prominent partitioning of Ginir RNA to the nucleus (Fig 6E–6H) in NIH/3T3-Ginir cells, providing a direct support to the data obtained by RT-PCR experiments. Secondly, the localisation of Ginir RNA seen with the same two fluorescence in situ hybridisation (FISH) probes was specific, since a much reduced nuclear staining was observed in the Ginir knock-down cells (Fig 6E and 6F). Cells treated with RNase A lacked any hybridisation fluorescence signal, confirming the specificity of binding of LNA probes to Ginir RNA but not to DNA (S7A and S7B Fig). Like the data obtained with RT-PCR, the Giniras RNA localisation by LNA probe was seen mainly in the cytoplasmic compartment (S7C Fig). In B16F10 melanoma cells, the Ginir RNA showed a prominent localisation to the nuclear compartment (Fig 6E–6H). In summary, we demonstrate that the Ginir and Giniras RNAs are primarily compartmentalised to the cytoplasm in normal cells; however, Ginir RNA is mainly partitioned to the nuclear compartment in transformed cells.

### Ginir RNA expression in cells induces dsDNA breaks and activates DNA damage response (DDR) pathway proteins

**Fig 7:**
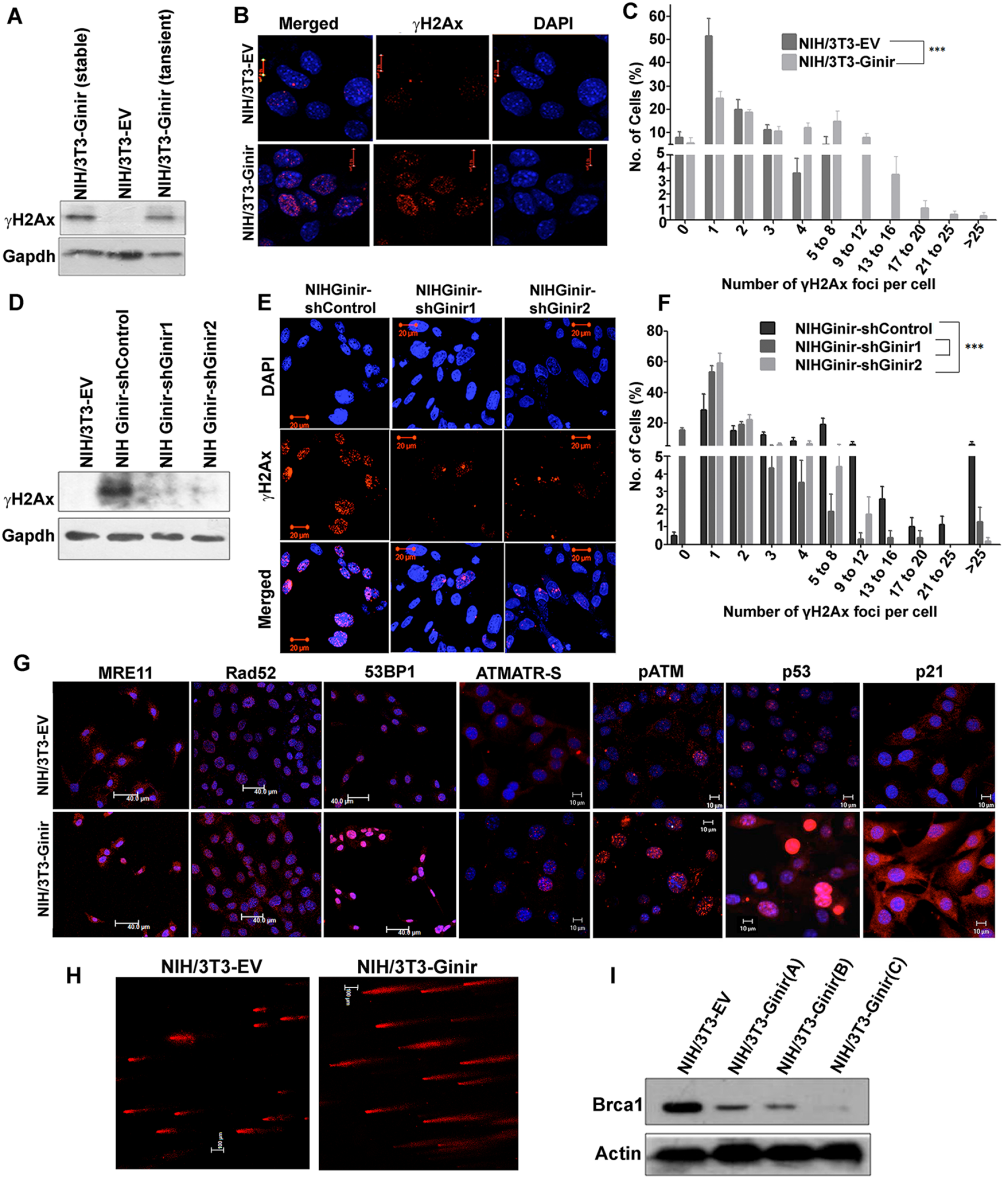
Ginir RNA induces defects in DDR and increases DNA double-stranded breaks. (A) Representative blot for expression of γH2AX, a DNA damage marker protein in NIH/3T3, NIH/3T3-Ginir (stable cell-line), and NIH/3T3-Ginir cells transiently transfected with Ginir cDNA (48 hours). Thirty μg of whole-cell protein lysates prepared from the mentioned cell lines was loaded on 16% SDS-PAGE. Gapdh protein served as an internal loading control. Data shown are representative of 3 independent experiments done with 3 independent transient and stable Ginir transfectants. (B) Representative images showing γH2AX expression in NIH/3T3 and NIH/3T3-Ginir (48 hours post transfection) cells. Blue colour indicates nuclear staining with DAPI. Scale bars 10 μm. (C) Quantification of γH2AX foci in mentioned cell lines wherein the percentage of cells demonstrating a given range of foci is plotted. The number of foci per cell were counted using the software tool Image J; version 1.41. At least 75 cells from 10 random fields were counted from both NIH/3T3 and NIH/3T3-Ginir cells (*n* = 5). (D) Representative western blotting for γH2AX protein in indicated cells. Thirty μg of whole-cell protein lysates was run on a 16% SDS PAGE and further processed for detection by chemiluminescence. Gapdh protein served as a loading control. (E) Images showing γH2AX expression in NIHGinir-shGinir scrambled and NIHGinir-shGinir 1 and NIH-Ginir shGinir2 cells. Blue colour indicates nuclear staining with DAPI. Scale bars 20 μm. (F) Quantification of γH2AX foci in mentioned cell lines using Image J tool; the number of foci per cell was counted for each cell type, and the percentage of cells showing specific number of foci is plotted. At least 100 cells from 10 random fields were counted for the mentioned cells. Estimation of foci number in each cell line was done at least 5 times. (G) Confocal images showing the activation of DDR proteins in NIH/3T3-EV and NIH/3T3-Ginir cells. Nuclei were stained with DAPI (blue). Scale bars 10 μm. (H) NIH/3T3 and NIH/3T3-Ginir cells analysed for DNA damage using Comet assay. (I) Brca1 protein expression in cells of NIH/3T3, NIH/3T3-Ginir in three independent transfectants A, B, and C. Fifty μg of whole-cell protein of mentioned cell lines was analysed on 7% SDS-PAGE. Gapdh protein served as an internal loading control. Supporting data for C and F can be found in S4 Data. ATM/ATR-S, ataxia–telangiectasia-mutated kinase and ataxia–telangiectasia and Rad3-related kinase substrate; Brca1, breast cancer type 1 susceptibility protein; DDR, DNA damage repair; Gapdh, glyceride 3-phosphate dehydrogenase; Ginir, Genomic Instability Inducing RNA; Mre11,meiotic recombination 11; pATM, ataxia–telangiectasia-mutated kinase.

As a part of dsDNA damage response, ataxia–telangiectasia-mutated (ATM) kinase phosphorylates several downstream target proteins, which include the histone protein H2Ax, converting it to γH2AX [41]. We found that Ginir RNA overexpression either transiently or stably in NIH/3T3 cells increased the levels of γH2AX (Fig 7A); also evident were high numbers of repair foci in their nuclei (Fig 7B and 7C). In comparison, such repair foci were considerably less in NIH/3T3-Giniras and NIH/3T3-EV cells. The observation that even transient expression of Ginir RNA in NIH/3T3 cells caused an increased expression of γH2AX indicated that the increase was due to a primary effect caused by Ginir expression and was not due to secondary effects of cell transformation. This notion was further supported by the observations that the number of γH2AX repair foci significantly decreased after Ginir expression was knocked down, as evident in NIHGinir-shGinir1 and shGinir2 cells (Fig 7D–7F). Consistent with the increased level of γH2AX foci, NIH/3T3-Ginir(A) or NIH/3T3-Ginir(B) cells showed activation of member proteins involved in DDR, which included meiotic recombination 11 (Mre11), Rad52, p53 binding protein 1 (53BP1), ATM/ataxia–telangiectasia and Rad3-related kinase (ATR)-Substrate, pATM, p53, and p21. (Fig 7G). The DDR elicited by Ginir was further corroborated using a more direct DNA damage assay such as the comet assay, which demonstrated the presence of DNA damage signatures in NIH/3T3-Ginir cells (Fig 7H). A significant finding was that NIH/3T3-GinirA, B, and C independent transfectant lines show lower amounts of Brca1 protein in them compared to NIH/3T3-EV cells (Fig 7I). Also, these cells accumulated a higher level of nuclear p53 and exhibited cytoplasmic expression of p21 protein, which are known to work together to impair DNA repair, compromise the tumour suppressor function of p53, and promote genomic instability. Thus, a higher Ginir RNA expression led to activation of DDR, resulting in increased cell survival, as no apoptosis was detected, and cells continued cycling with error-prone DNA synthesis. Taken together, our data indicate that Ginir overexpression leads to genomic instability.

### Overabundance of Ginir RNA induces desynchronisation of karyokinesis from cytokinesis and generates multinucleated cells

**Fig 8:**
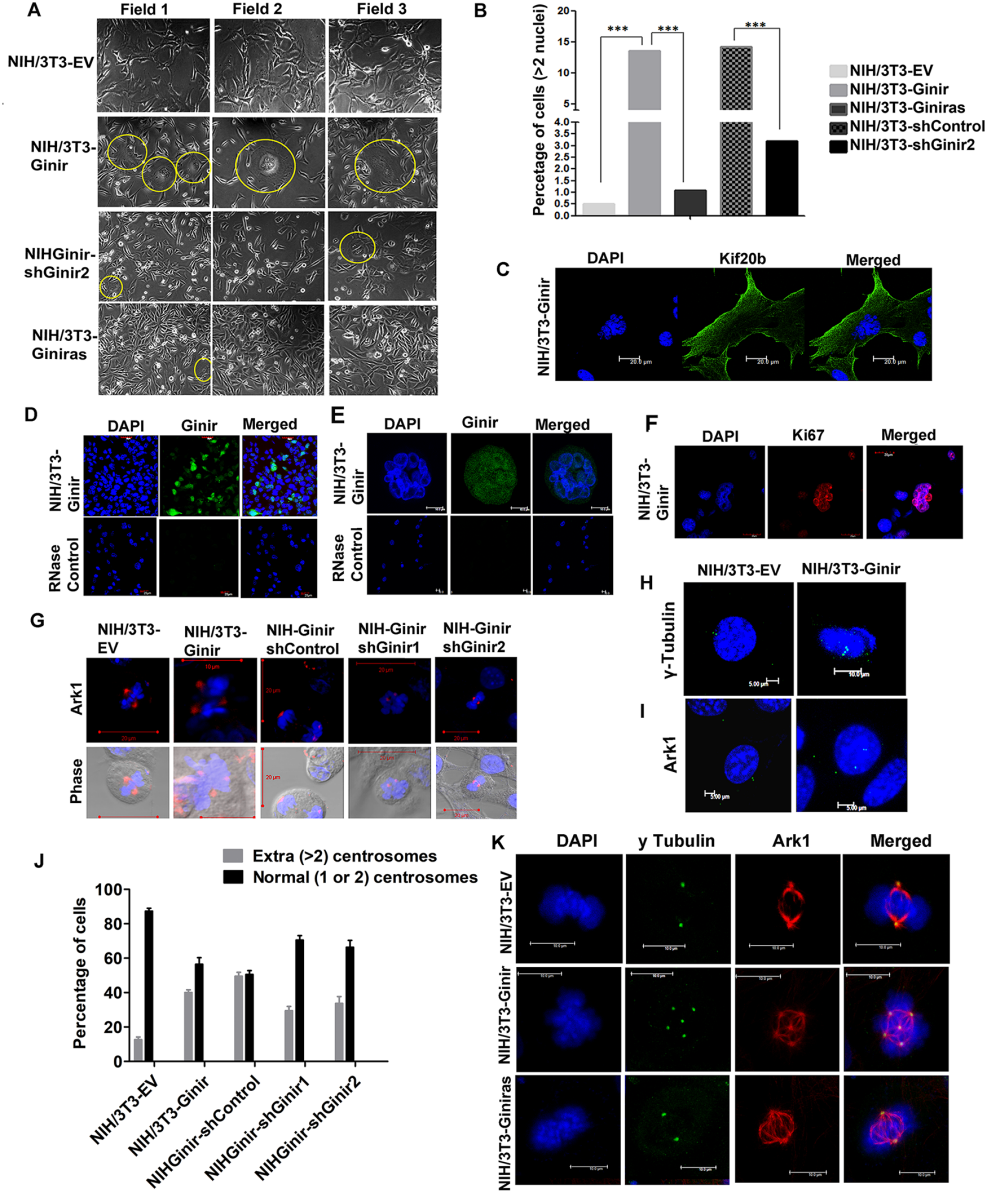
Ginir induces mitotic defects and impairs cytokinesis, forming multinucleated giant cells. (A) Phase contrast micrographs demonstrating multinucleated giant cells present in mentioned cell lines. Encircled areas highlight the multinucleated giant cells amongst mononucleated proliferating cells (10× magnification). (B) Quantification of multinucleated cells in the indicated cell lines, expressed as percentage of cells with >2 nuclei. Nuclei were stained with DAPI. Images were acquired using confocal microscope. About 400 cells were counted manually for all cell lines except for NIH/3T3-Giniras, for which only about 200 cells were counted. Bars represent mean values ± SEM; **P values > .001 using Student’s *t* test. (C) Representative Kif20b staining in NIH/3T3-Ginir cells demonstrating the presence of multinucleated giant cells. Nuclei were stained with DAPI (blue) and viewed under confocal microscope. Scale bar 20 μm. (D and E) Representative RNA-FISH images stained with Ginir-specific LNA probe-1 (green) in NIH/3T3-Ginir cells (D). Magnified image of one of the multinucleated cells in NIH/3T3-Ginir cells. Nuclei were stained using DAPI (E). Scale bars 10 μm. (F) Representative Ki67 antigen staining in multinucleated giant cells of NIH/3T3-Ginir cells, demonstrating its strong expression. Nuclei were stained with DAPI. Scale bar 20 μm. (G) Immunofluorescence staining of the centrosome marker protein Ark1 in multinucleated giant cells. Nuclei were stained with DAPI. Scale bars 20 μm. (H) Immunofluorescence detection of the centrosome marker protein ɣ-tubulin in indicated interphase cells. Nuclei were stained with DAPI. Scale bars 5 and 10 μm. (I) Immunofluorescence detection of the centrosome marker protein Ark1 in mentioned interphase cells. Nuclei were stained with DAPI. Scale bars 5 μm. (J) Histogram plot demonstrating centrosome numbers in indicated cell lines. The percentage of cells with amplified (i.e., more than 2) and cells with a normal number of centrosomes (1 to 2) were visually scored from both metaphase and interphase cells. A minimum of 150 cells were counted for centrosome number by staining with γ-tubulin. The scoring was based on data from at least 3 independent experiments. Data shown represent mean ± SEM. ***P ≤ 0.0001, one tailed, one-way ANOVA test. (K) Images representing coexpression of a centrosome marker protein γ-tubulin with Ark1 in mitotically active cells. Nuclei were stained with DAPI. Scale bars 10 μm. Supporting data for B and J can be found in S5 Data. Ark1, aurora-related kinase 1; Ginir, Genomic Instability Inducing RNA; Kif20b, kinesin family member 20; RNA-FISH, RNA fluorescence in situ hybridisation.

The NIH/3T3-Ginir cells always showed a preponderance of large numbers of multinucleated giant cells (Fig 8A), which were rare in number in NIH/3T3-EV and NIH/3T3-Giniras cells and were significantly reduced upon Ginir knock-down in NIH/3T3-Ginir cells (Fig 8A and 8B, and S8A Fig). The down-regulation of Ginir RNA in NIH/3T3-Ginir cells led to restoration of phenotype to that exhibited by NIH/3T3-EV cells, highlighting the specificity of giant cell induction by Ginir RNA (Fig 8A and 8B and S8A Fig). The giant cell formation was prominently visible using the antibody directed against kinesin family member 20b (Kif20b), a microtubule-associated protein marker (Fig 8C).

In addition, the in situ localisation of Ginir RNA using LNA-FISH probes demonstrated a distinct Ginir RNA compartmentalisation to the nuclei of the transformed cells (Fig 8D) as well as to the multinucleated giant cells (Fig 8E and S8C Fig). Strong oncogenic signals are known to induce senescence, and it is possible that the giant cells arose because of Ginir RNA. The giant cell formation in NIH/3T3-Ginir cells was found unrelated to the normal senescence response by the criteria that these cells were not growth arrested and showed the expression of the proliferation marker Ki67 (Fig 8F). Furthermore, defects in spindle formation along with abnormal amplification of centrosome numbers were evident in multinucleated giant cells of NIH/3T3-Ginir cells (Fig 8G). These features appeared specific to high Ginir RNA expression in these cells, since the NIHGinir-shGinir1 and NIHGinir-shGinir2 cells did not exhibit these defects (S8A Fig). A large number (approximately 40%) of NIH/3T3-Ginir cells showed greater than 2 centrosomes per cell, and this abnormality in centrosome numbers induced because of Ginir were evident by staining of these cells with centrosomal markers like ɣ-tubulin (Fig 8H) and aurora-related kinase 1 (Ark1) (Fig 8I). The numerical effects on centrosome numbers (>2 centrosomes/cell) induced by Ginir overexpression were quantified and occurred in a NIH/3T3-Ginir cell population to a larger extent (approximately 45%–50%) (Fig 8J) as compared to control cells (*P* ≤ 0.0001). These defects in centrosome numbers were evident in both interphase (Fig 8H and 8I) and metaphase cells (Fig 8K). More notably, knock-down of Ginir caused reversal to regulated mitosis manifested in terms of formation of regular bipolar spindles as evident by Ark1 staining (Fig 8G). Thus, high levels of Ginir RNA expression apparently accelerated karyokinesis but retarded cytokinesis, thereby generating multinucleated giant cells. The accelerated karyokinesis that was desynchronised from cytokinesis represented another manifestation of the failure of cell cycle arrest at spindle checkpoint, triggering a premature and abnormal spindle dynamics. A low Ginir RNA level may be necessary to maintain a normal and synchronised spindle dynamics, karyokinesis, and cytokinesis within these cells. Defects in cytokinesis and karyokinesis are known to induce chromosomal and genomic instability [42]. In summary, these data show that excess Ginir RNA expression causes centrosomal defects and genomic instability, leading to mitotic dysregulation.

### Ginir RNA modulates centrosome function by interacting with Cep112

**Fig 9:**
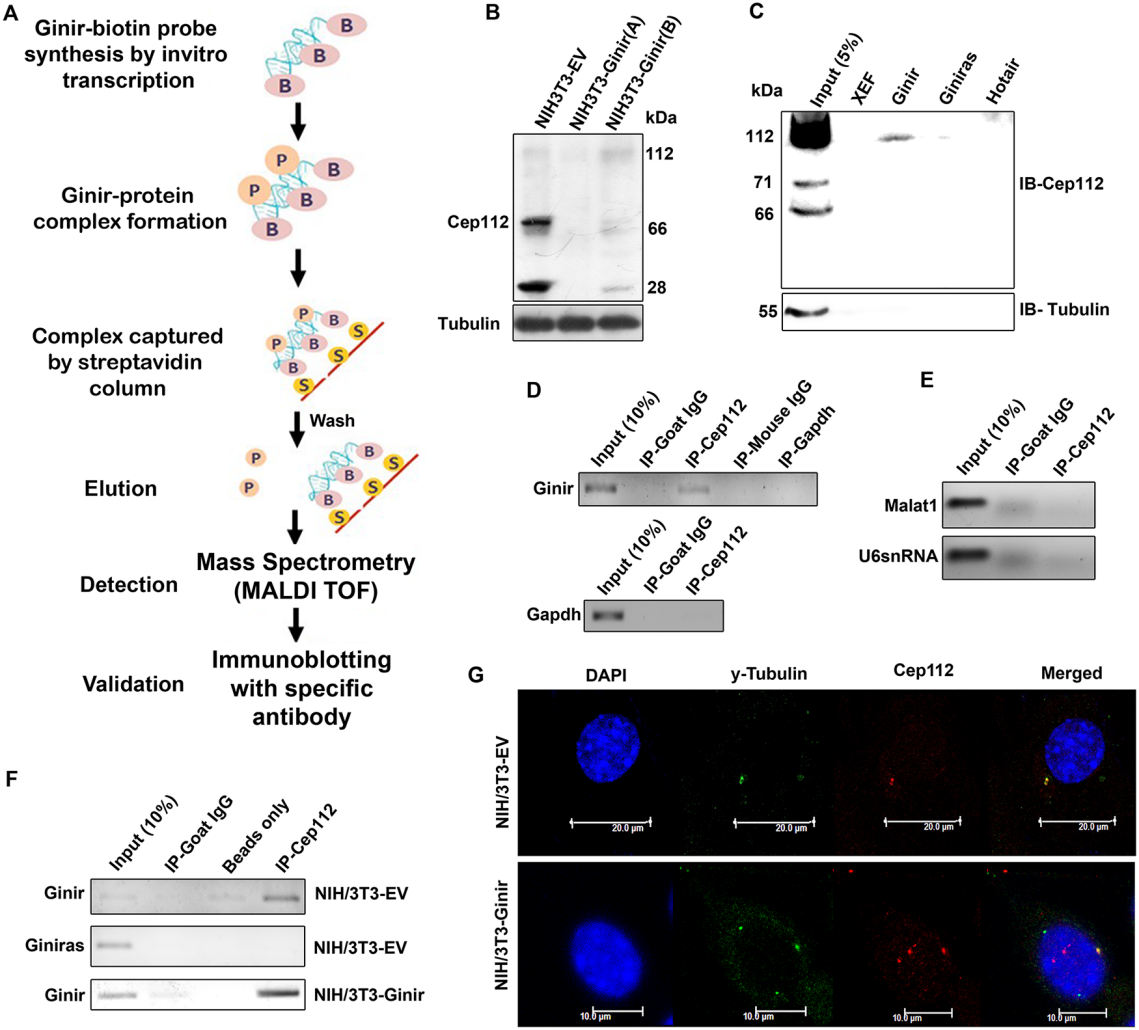
Ginir physically interacts with centrosomal protein Cep112 in cells. (A) Schematic representation of RNA pull-down assay strategy using biotinylated Ginir RNA. (B) Western blot analysis for Cep112 protein expression. Fifty μg of whole-cell protein lysates of mentioned cell lines were loaded on 10% SDS-PAGE and probed using Cep112 antibody. (sc-246162, Santa Cruz). Tubulin protein served as internal loading control. (C) Western blot of Ginir RNA–associated proteins identified by RNA pull-down assays. NIH/3T3 cell proteins and 5′-biotinylated Ginir and Giniras RNAs were used in the assay. Elutes from RNA–protein complexes were loaded on 7% SDS-PAGE for protein separation. Blot was probed with Cep112 antibody (24928-1-AP, Proteintech). Biotinylated Giniras probe served as negative control. Unrelated biotin-labelled RNA probes like XEF RNA and Hotair RNA were used as nonspecific controls. Blot was stripped and reprobed for tubulin as control to rule out possibility of any nonspecific binding. (D) RIP with Cep112 antibody by using cell lysate of NIH/3T3-Ginir cells. RIP was followed by RT-PCR with Ginir RNA–specific primers (G2F-G2R). RT-PCR with Gapdh-specific primers served as control for nonspecific amplification in RIP. Also, RIP using anti-IgG and anti-Gapdh antibodies served as negative control for nonspecific binding. (E) RIP detection with Cep112 antibody for proteins isolated from NIH/3T3-Ginir cells followed by RT-PCR using primers that amplify Malat1 and U6 snRNA. These served as controls for nonspecific binding. (F) RIP assay with Cep112 antibody for analyses of interacting proteins sourced from NIH/3T3 and NIH/3T3-Ginir cells. Strand-specific RT-PCR was done using Ginir- and Giniras-specific primers (G2F-G2R). RIP with anti-IgG served as negative control for nonspecific binding. (G) Colocalisation of Cep112 with centrosomal marker γ-tubulin in mentioned interphase cells. Nuclear staining was done using DAPI. Scale bars, 10 μm. Cep112, centrosomal protein 112; Gapdh, glyceride 3-phosphate dehydrogenase; Ginir, Genomic Instability Inducing RNA; Giniras, antisense RNA to Ginir; Hotair, HOX transcript antisense RNA; Malat1, metastasis-associated lung adenocarcinoma transcript 1; RIP, RNA-immunoprecipitation; MALDI-TOF, matrix-assisted laser desorption ionisation time-of-flight mass spectrometry; RT-PCR, reverse transcription polymerase chain reaction; snRNA, small nuclear RNA; XEF, Xenopus elongation factor.

A multitude of lncRNAs are known to mediate their functions by interacting directly with specific protein targets within cells. To determine whether such target proteins could be identified for Ginir RNA, we followed the approach of using biotin-labelled RNA affinity pull-down and RNA-immunoprecipitation (RIP) assays. In the pull-down assay, 5′-biotin-labelled full-length Ginir RNA was incubated with protein lysates prepared from various cell types for possible interactions to occur, and then, the proteins bound to Ginir RNA were pulled down by streptavidin capture beads. This strategy is detailed in Fig 9A. The bound proteins recovered from the pull-down complexes were identified by mass spectrometry (S9A Fig and S2 Table). Frequently detected interacting proteins of Ginir RNA obtained from NIH/3T3, NIH/3T3-Ginir cells, and mouse embryonic brain tissues were shortlisted on the basis of their high confidence index for matrix-assisted laser desorption ionisation time-of-flight mass spectrometry (MALDI-TOF) and sorted on the basis of their computed interaction scores using bioinformatics tools like RPISeq [43] and catRAPID [44,45] (S9B Fig and S1 Text). The data from multiple independent experiments using cells from different tissue sources and cultured cell lines consistently demonstrated Cep112, a centrosome-associated protein, as a strong interacting partner for Ginir RNA, as it had the highest interaction score (S1 Text). Cep112 (the centrosomal protein of 112 kDa), also known as *Ccdc46* or *Macoco*, is a centrosomal protein with ATPase domain and has 18 possible splice variants that can give rise to 12 protein isoforms (S9C Fig). We found that NIH/3T3 cells expressed multiple isoforms of Cep112, and most prominent amongst them were the 112-, 66-, and 28-kDa isoforms (Fig 9B and S9C Fig). This raised the question if some or all the Cep112 isoforms were interacting with Ginir RNA. The biotin RNA pull-down assays identified one prominent interacting isoform of 112-kDa size for Ginir RNA (Fig 9C and S9D Fig). Another isoform of Cep112 was of 66-kDa size, and it also showed interaction with Ginir, though detection of this isoform expression varied with the antibody source used (S9D Fig). The interaction of Cep112 appeared specific to Ginir RNA, as other noncoding RNAs like HOX transcript antisense RNA (Hotair) and Giniras failed to interact with the same protein (Fig 9C and S9D Fig). Similarly, another unrelated RNA from *Xenopus* species, termed as the Xenopus elongation factor (XEF) RNA, failed to interact with Cep112 protein (Fig 9C). To eliminate the possibility of Ginir RNA binding nonspecifically to any protein, we performed blotting with β-tubulin antibody and found that there was no interaction of Ginir RNA with β-tubulin (Fig 9C and S9D Fig). This series of pull-down experiments clearly demonstrated the specificity of interaction of Ginir RNA with centrosomal protein Cep112.

### Ginir RNA interacts with Brca1 and Cep112 proteins to affect their expression levels and cellular redistribution

**Fig 10:**
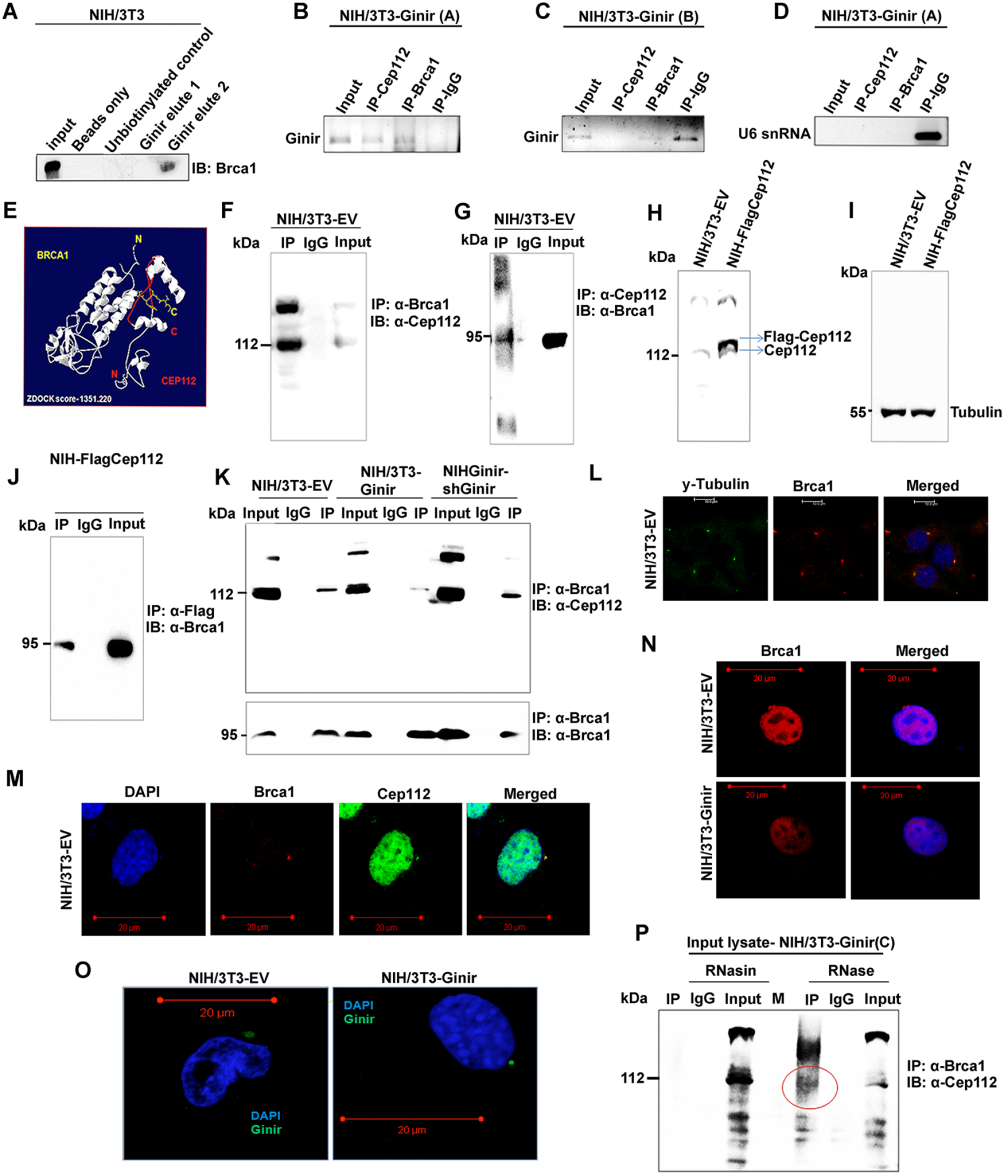
Ginir RNA impairs interaction between Cep112 and Brca1 proteins leading to genomic instability. (A) RNA pull-down with biotinylated Ginir RNA in NIH/3T3 cells followed by western blotting with Brca1 antibody (sc-646, Santa Cruz). Pull-down with unbiotinylated RNA probe served as control for nonspecific binding. (B-D) RIP performed using both Cep112 and Brca1 antibodies followed by RNA isolation and RT-PCR with Ginir specific primers (G2F-G2R) in NIH/3T3-GinirA (B) and NIH/3T3-GinirB (C) cells. RIP assay was also followed by RT-PCR using nonspecific primers for U6 snRNA (D). Anti-IgG IP served as control for nonspecific interaction. (E) In silico model of Cep112 and Brca1 interaction generated through computational docking using ZDOCK tool. (F and G) Co-IP of Cep112 and Brca1 proteins in NIH/3T3-EV cells. IP was performed with Brca1 antibody (sc-646, Santa Cruz) followed by immunoblotting with Cep112 antibody (sc-246162, Santa Cruz) (F) and vice versa (G). Anti-IgG IP served as a control for nonspecific binding to the antibody. (H and I) Western blotting with Cep112 antibody (24928-1-AP, Proteintech) for validation of Flag-Cep112 overexpression in NIH/3T3 cells (H). Fifty μg of whole-cell protein lysates from each of the mentioned cell lines was loaded on 7% SDS-PAGE. Tubulin served as internal loading control (I). (J) Co-IP of Brca1 and Flag-Cep112 in NIH-Flag-Cep112 cells. IP was performed with Flag1 antibody followed by immunoblotting with Brca1 antibody (20649-1-AP, Proteintech). Anti-IgG IP served as a control for nonspecific binding to the antibody. (K) Co-IP of Brca1 and Cep112 proteins in NIH/3T3-EV, NIH/3T3-Ginir, and NIH-Ginir-shGinir2 cells. IP was performed with Brca1 antibody (sc-646, Santa Cruz) followed by immunoblotting with Cep112 antibody (24928-1-AP, Proteintech). Anti-IgG IP served as a control for nonspecific binding to the antibody. (L) Confocal images showing colocalisation of Brca1 with γ-tubulin in NIH/3T3-EV cell line. Nuclei were stained with DAPI. Scale bars, 10 μm. (M) Confocal images showing colocalisation of Brca1 protein with Cep112 protein in NIH/3T3-EV cell line. Nuclei were stained with DAPI. Scale bars, 20 μm. (N) Confocal imaging for Brca1 expression in NIH/3T3-EV and NIH/3T3-Ginir cells. Scale bars, 20 μm. (O) RNA-FISH using Ginir-specific probe (probe 1, FAM labelled) in NIH/3T3-EV cells visualised by confocal imaging. Scale bars, 20 μm. (P) Co-IP of Brca1 and Cep112 in NIH/3T3-Ginir(C) cells wherein lysates were treated independently with RNase (A, H, and III mix) or RNasin. Both RNase- and RNasin-treated lysates were immunoprecipitated with Brca1 antibody (sc-646, Santa Cruz) and blotted using Cep112 antibody (sc-246163, Santa Cruz). IP with anti-IgG served as control. Brca1, breast cancer type 1 susceptibility protein; Cep112, centrosomal protein 112; FAM, fluorescein amidite; Ginir, Genomic Instability Inducing RNA; IgG, immunoglobulin G; IP, immunoprecipitation; RIP, RNA-immunoprecipitation; RNasin, RNase inhibitor; RT-PCR, reverse transcription polymerase chain reaction; snRNA, small nuclear RNA.

The important components of the DNA damage response pathway are the twin repair proteins Brca1 and Brca2. Loss-of-function mutations in Brca1 and Brca2 result in increased mutation rates and induction of genomic instability [46,47]. Low Brca1 protein expression levels are frequently found in various transformed cells or tumour cells. Consistent with this observation, we found a decreased expression level of Brca1 protein in NIH/3T3-Ginir cells as compared to NIH/3T3-EV cells (Fig 7I). We therefore sought to determine whether a low level of Brca1 expression in these cells was induced because of high levels of Ginir RNA expression. For this, we performed biotin RNA pull-down experiments using biotinylated Ginir RNA followed by immunoblotting with Brca1 antibody. In these experiments, a physical interaction of Ginir with Brca1 in vitro could be detected (Fig 10A and S10A Fig) in elute II but not in elute I (Fig 10A). The interaction was specific, as it was seen only with Ginir RNA but not with other noncoding RNAs like Hotair and Giniras and mRNA like XEF (S10A Fig). Similarly, with RIP performed using two independent NIH/3T3-Ginir RNA expressing clones A and B, we obtained evidence for a weaker interaction of Ginir RNA with Brca1 as compared to Cep112 (Fig 10B and 10C). The specificity of interaction was further confirmed using U6 small nuclear RNA (snRNA) as a control (Fig 10D).

Besides its involvement in DNA repair, Brca1 protein has roles in centrosome function, and in cooperation with other centrosomal proteins, it plays a vital role in the nucleation and assembly of microtubules during chromosomal segregation [48]. For instance, Brca1 protein regulates centrosome duplication and cytokinesis by interacting with other centrosomal proteins like Obg-like ATPase 1 (OLA1) [49], ninein-like protein(Nlp) [50], and γ-tubulin [51]. These reports and our data prompted us to examine if interactions of Ginir RNA occur with either Cep112 and Brca1 proteins independently of each other or whether the Cep112 protein interacts with Brca1 protein. To determine this, we performed protein docking analysis using ZDOCK tool and found significant docking scores suggestive of a stable interaction between the C-terminal of Brca1 protein with a domain near the C-terminal region of Cep112 protein (Fig 10E). Next, to investigate the interaction between Cep112 and Brca1 proteins, we performed coimmunoprecipitation of Cep112 with Brca1 (Fig 10F) and vice versa (Fig 10G). Here, we found a strong interaction between these two proteins in NIH/3T3 cells. In another set of experiments, we overexpressed Flag-Cep112 in NIH/3T3 cells and obtained stable clones of cells expressing Flag-Cep112 protein, as was confirmed by western blotting (Fig 10H and 10I). The interaction was further validated in Flag-Cep112-expressing cells, wherein we demonstrated coimmunoprecipitation of Brca1 with Flag tag antibody (Fig 10J) and vice versa (S10B Fig). A weaker interaction of Cep112 protein with Brca1 protein was found in NIH/3T3-Ginir(A) cells (Fig 10K). In NIHGinir-shGinir RNA knock-down cells, the interaction of Cep112 with Brca1 was significantly restored (Fig 10K). These data strongly indicate that Cep112 and Brca1 proteins interact strongly in NIH/3T3-EV or NIH/3T3-Ginir-shGinir1 knock-down cells, but the interaction is weaker in NIH/3T3-Ginir(A) cells, where Ginir RNA expression level is high.

The localisation of Brca1 to the centrosomes was distinctly evident by coexpression of Brca1 with centrosomal marker protein γ-tubulin (Fig 10L). Further, we detected interaction of Cep112 protein with Brca1 protein in centrosomes only in the NIH/3T3-EV cells (Fig 10M) but not in the NIH/3T3-Ginir(A) cells. This could mainly be attributed to the fact that each of these two proteins was mislocalised from the centrosomes in these cells. Besides normal localisation of Brca1 to the nucleus, there was a specific enrichment of Brca1 protein to the centrosomes in NIH/3T3-EV cells. In contrast, in NIH/3T3-Ginir cells, Brca1 was seen mainly in the nucleus but was entirely absent from the centrosomes (Fig 10N and S10E Fig). The bioinformatics tools like CatRAPID also showed high interaction propensity (= 37) as well as high discriminative power (= 85) of Brca1 to bind to Ginir RNA (S10F Fig). By RNA-FISH, we obtained a specific localisation of Ginir RNA to the perinuclear region of the cells that may be centrosome (Fig 10O). These data assume importance and may have significance in proliferation and oncogenesis, as this is the first evidence, to our knowledge, demonstrating interaction of two proteins Cep112 and Brca1 with each other and their dynamic localisation into centrosome, nuclear, and cytoplasmic compartments.

### Ginir RNA disrupts interaction between Brca1 and Cep112 proteins

Next, we asked (1) as to how a higher Ginir RNA level induced a lower expression level of Brca1 and Cep112 proteins and (2) whether interaction of Cep112 protein with Brca1 protein was mediated through Ginir RNA. For this, we prepared cell lysates from NIH/3T3-Ginir cells (stable cell lines NIH-Ginir A, B, and C) and divided each of the protein lysates into two parts; one was treated with RNase mix (A, H, and III) to deplete RNA from them, and the second part received RNase inhibitor (RNasin) to protect the integrity of all RNAs present. When we used a specific Brca1 antibody to pull down Brca1 from each of these two samples, we found that the Brca1–Cep112 protein complex was present in the Rnase-treated cell lysates but was nearly absent in the lysates that were pretreated with RNasin (Fig 10P and S10C and S10D Fig). This demonstrated that binding of an RNA to the Cep112–Brca1 protein complex disrupted their interaction or that this interaction did not occur in the presence of the RNA. In conclusion, we provide a strong evidence that a higher amount of Ginir RNA impairs interaction of Cep112 and Brca1 proteins, leading to their mislocalisation in the cells and thereby resulting in mitotic dysregulation (Figs 10N and 9G, S9H and S10E Figs).

### Genomic instability results from the down-regulation of Brca1 and Cep112 proteins

**Fig 11:**
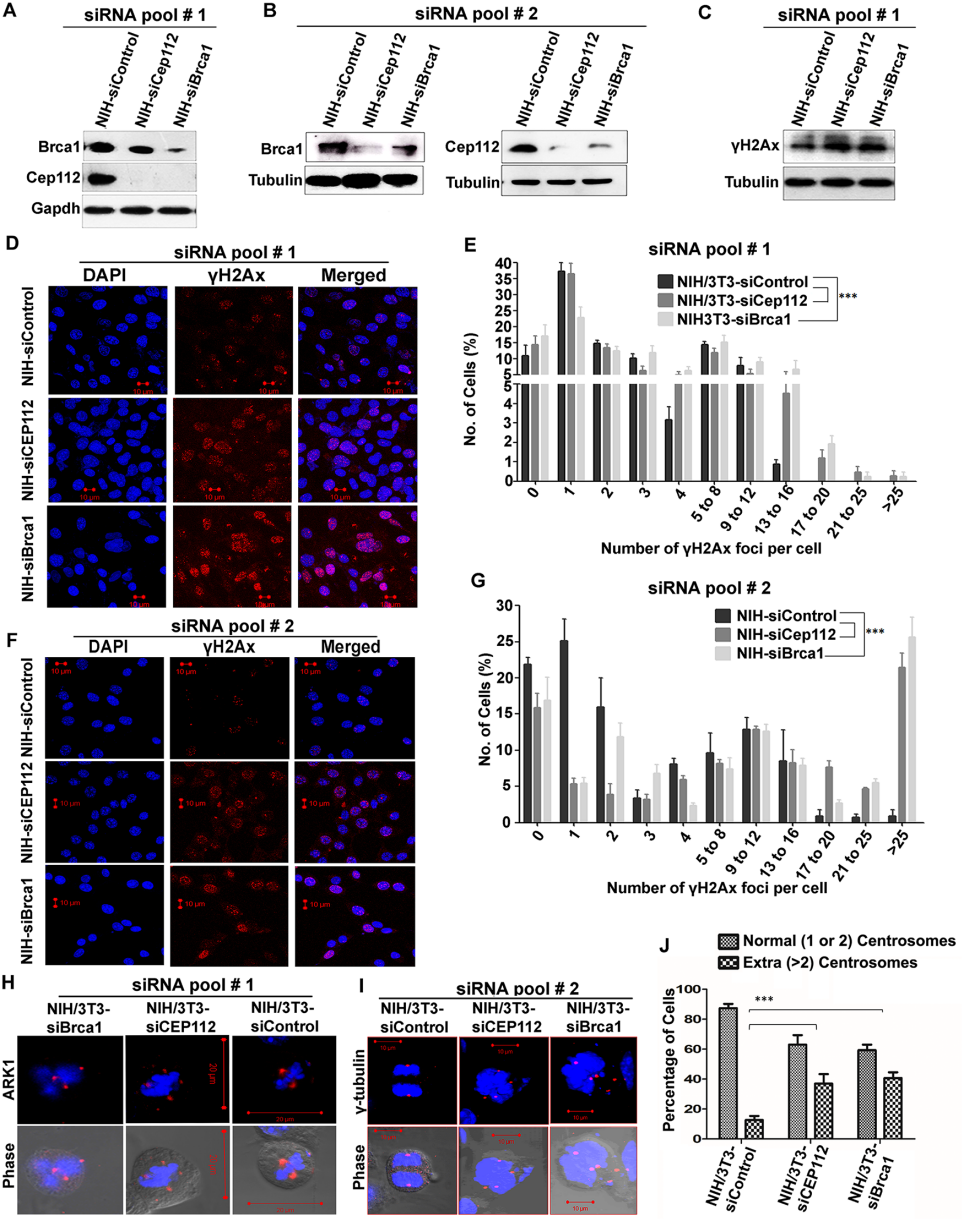
Down-regulation of Brca1 and Cep112 proteins leads to increased genomic instability in NIH/3T3 cells. (A and B) Representative immunoblots for Brca1 and Cep112 (sc-246163, Santa Cruz) proteins in NIH-siControl, NIH-siCep112, and NIH-siBrca1 cells. Cells were transfected with respective independent siRNA pools—siRNAs pool # 1 (Santa Cruz) (A) and siRNAs pool # 2 (Stealth RNAi, Thermo Fisher Scientific) (B). Fifty μg of whole-cell protein lysates of mentioned cell lines were loaded on 10% (A) and 7% (B) SDS-PAGE. Gapdh (A) and tubulin (B) proteins served as loading controls for the western blot. (C) Representative blot for γH2AX expression of NIH/3T3 cells transfected independently with Cep112 and Brca1 siRNA (pool # 1). Fifty μg of whole-cell protein lysates of each transfectant cell line was loaded on 16% SDS-PAGE. Gapdh protein served as loading control. (D-G) Confocal microscopy for detecting γH2AX foci along with nuclear staining with DAPI in siRNA pool # 1 (D) and siRNA pool # 2 (F) transfected NIH/3T3 cells. Scale bars, 10 μm. Quantification of γH2Ax foci in pool # 1 (E) and pool # 2 (G) cell lines using Image J tool. The number of foci per cell was counted in >75 cells (E) and >150 cells (G). The percentage of cells showing a given number of foci is plotted. Data shown represent mean ± SEM. ***P ≤ 0.0001, one tailed, one-way ANOVA test. (*n* = 3). (H and I) Immunofluorescence staining with Ark1 (H) and γ-tubulin (I) antibody in NIH-siControl, NIH-siCep112, and NIH-siBrca1 (pool # 1[H], pool # 2[I]) cells. Nuclei were stained with DAPI. Scale bars, 20 μm (H), 10 μm (I). (J) Histogram plot demonstrating centrosome numbers in represented cell lines. The percentage of cells with normal number of centrosomes (1 to 2) or cells with amplified (i.e., more than two centrosomes) were visually scored in both metaphase and interphase cells. The scoring was done based on γ-tubulin staining examined in at least 150 cells. A minimum of three independent experiments were done for each cell line. Data shown represent mean ± SEM. ***P ≤ 0.0001, one tailed, one-way ANOVA test. Supporting data for Figs E, G, and J can be found in S6 Data. Ark1, aurora-related kinase 1; Brca1, breast cancer type 1 susceptibility protein; Cep112, centrosomal protein 112; Gapdh, glyceride 3-phosphate dehydrogenase; siRNA, small interfering RNA.

To understand as to how Ginir RNA regulates spindle dynamics and to generate independent evidence for the hypothesis of whether a collaboration of Cep112 protein with Brca1 protein is involved in this process, we examined NIH/3T3 cells in which the Cep112 and Brca1 proteins were individually down-regulated using two independent pools of specific small interfering RNAs (siRNAs) to each of these proteins (Fig 11A and 11B). Cells that were depleted with Brca1 protein (NIH-siBrca1) showed complete absence of Cep112 protein, whereas cells depleted for Cep112 (NIH-siCep112) showed diminished levels of Brca1. These observations indicated that both these proteins were involved in their mutual stabilisation, and a knock-down of either proteins affected the cellular level of the other protein (Fig 11A and 11B). Thus, Ginir RNA, by interrupting interaction between these two proteins, destabilised them and decreased their individual levels. A feature that was common to the depletion of these two proteins was that the siBrca1- and siCep112-expressing cells had increased levels of γH2Ax (Fig 11C) and had an increased number of repair foci in their nuclei (Fig 11D–11G). Both the NIH-siCep112 (pool I) and NIH-siBrca1 (pool I) cells, when examined independently, shared a common feature, which was an overactivation of Ark1, and many cells showed abnormal spindle dynamics, a phenomenon also found in NIH/3T3-Ginir (A) cells (Fig 11H). Abnormal effects were also seen on centrosome numbers in NIH-siCep112 (pool II) and NIH-siBrca1 (pool II) cells, as was evident from ɣ-tubulin staining (Fig 11I). Cells deficient in either Cep112 or Brca1 individually showed an abnormal number of centrosomes. We quantified these data regarding abnormalities in centrosome number and found that almost 40% of the NIH-siCep112 and NIH-siBrca1 cells showed >2 centrosomes (Fig 11J).

**Fig 12:**
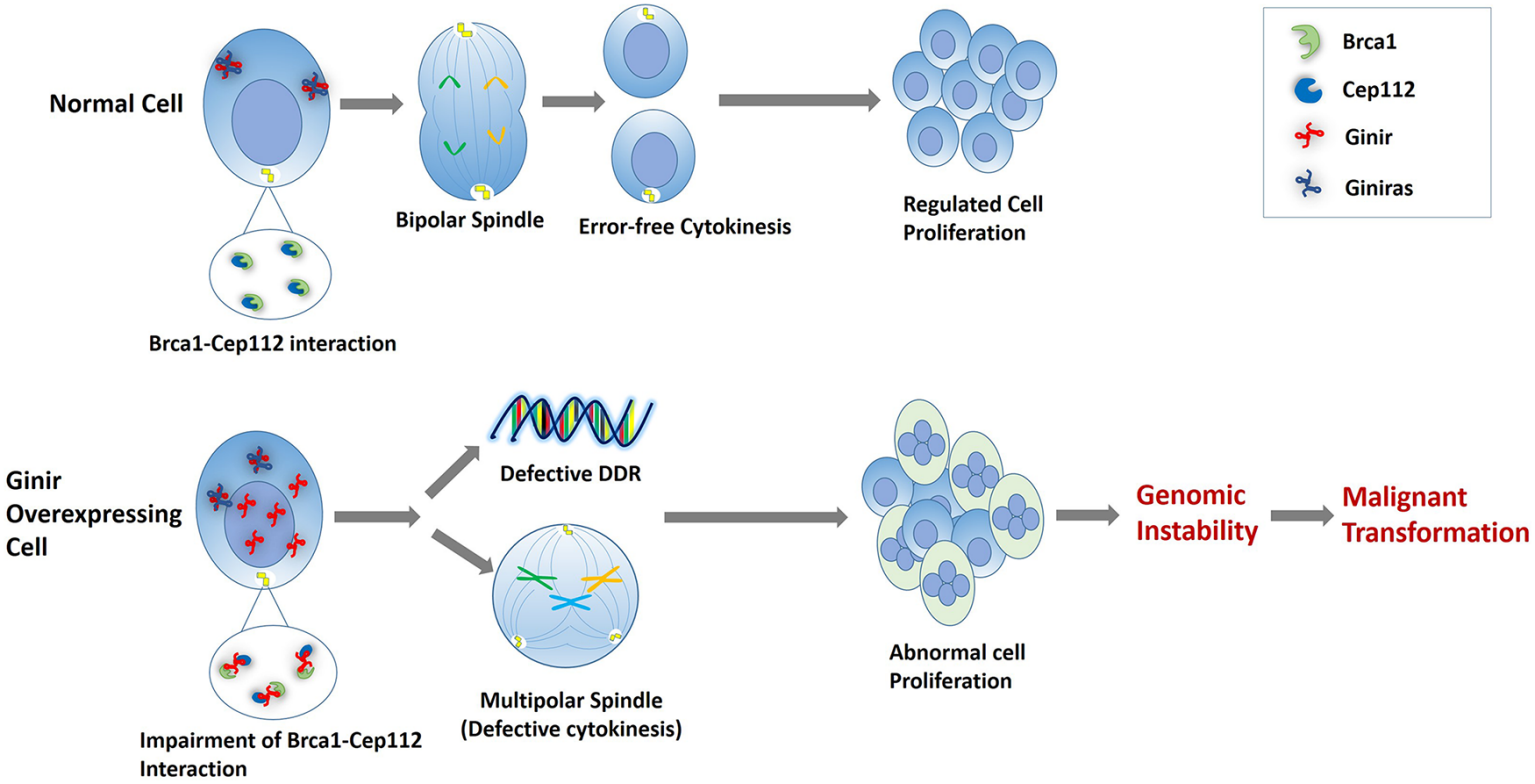
Model proposing mechanism through which Ginir mediates malignant transformation in mouse cells. Brca1, breast cancer type 1 susceptibility protein; Cep112, centrosomal protein 112; DDR, DNA damage response; Ginir, Genomic Instability Inducing RNA; Giniras, antisense RNA to Ginir.

In summary, we found that Cep112–Brca1 interaction was perturbed upon Ginir RNA overexpression, and this had significant bearing on normal mitotic regulation, a process required for progression of high-fidelity cell division, as depicted in the form of a model in Fig 12. The same is explained schematically in S11 Fig. The data reported here drive one to recognise the role of Ginir noncoding RNAs in mediating protein–protein interactions and regulating cell division with fidelity. Any dysregulation in these processes has high propensity to culminate in malignant transformation.

## Discussion

Here, we report the identification and functional characterisation of a novel lincRNA, termed Ginir because at high levels of its expression, it induces genomic instability in the target cells, leading to oncogenesis. We also provide mechanistic insight into its role as an RNA-promoting malignant transformation. This lincRNA was identified in Clone M3 mouse melanoma cells by a functional cloning approach, aimed to identify any activated, acutely transforming oncogene that might be functioning in Clone M3 melanoma cells. The expression clone inducing foci of transformed cells after transfection into the indicator NIH/3T3 recipient cells was found to contain a cDNA that was derived from a noncoding RNA named Ginir. Ginir is also expressed in cultured mouse embryonic fibroblast cells such as NIH/3T3 at low levels. However, when expressed at high levels in the same NIH/3T3 cells, it induces genomic instability and promotes cellular transformation. In this respect, it compares favourably with the known acutely transforming oncogenes that code for oncoproteins such as H-ras (Gly→Val), c-myc, et cetera. It is well known that c-myc expression remains at low levels in normal cells, but when the expression level of the c-myc protein is increased because of promotion of its transcription and translation by chromosomal translocations or retroviral promoter integration near to the gene, cellular transformation is promoted [52,53]. We found that Ginir RNA coexisted with a full-length antisense RNA (Giniras) in normal and malignant cells, suggesting that Giniras RNA may be a natural regulator of Ginir RNA functions in vivo. Ectopic expression of Giniras RNA using strong promoters did not show any special phenotype in NIH/3T3 cells per se, but when its overexpression was engineered in Ginir RNA–induced transformed NIH/3T3 cells, its oncogenic phenotype was blunted, supporting its role as a natural regulator of Ginir RNA function in vivo. We found evidence for Ginir RNA to have normal cellular functions, especially during mouse embryonic development, exhibiting a profile of expression that appeared to be developmentally regulated in a spatiotemporal manner.

Since the mammalian transcriptomes are rich in noncoding RNAs because of pervasive transcription, and since only about 2% of the noncoding RNAs are functionally annotated so far, it is very likely that many more lncRNAs will be found to act as protooncogenes. A gap exists in our knowledge about whether the majority of lncRNAs exhibit their biological function by following one or more mechanistic pathways or there are overlaps, or whether they elicit their transforming potential using mechanisms that are entirely distinct from those of protein-coding oncogenes. Hence, it is important to distinguish these RNA-mediated mechanisms from those of protein-mediated mechanisms of oncogenesis. Certainly, the mechanisms through which noncoding RNAs participate in transformation and tumour progression are varied and complex. Therefore, it is important to dissect out whether the lncRNAs regulate gene expression by functioning in *cis* or whether they act in *trans*. One of the recently reported Cancer Testis noncoding RNA, Thor, was shown to function as an oncogene by binding to an RNA-binding protein (RBP), insulin-like growth factor 2 mRNA-binding protein 1 (IGF2BP1), causing stabilisation of its target mRNAs like insulin-like growth factor 2 (IGF2) and CD44 [54]. Another noncoding RNA, hypoxia-inducible factor 1-alpha (HIF-1a) coactivating RNA (HIFCAR), was shown to exert its oncogenic role as an HIF-1a coactivator and thereby regulate the HIF-1 transcriptional network, crucial for cancer development [55]. Another noncoding RNA, noncoding RNA activated by DNA damage (NORAD), was induced after DNA damage and was shown to maintain genomic stability by sequestering PUMILIO proteins, which repressed the stability and translation of mRNAs involved in mitosis and DNA repair [56]. Most often, *trans*-acting lncRNAs function by modulating the activity or abundance of proteins or RNAs to which they directly bind. A key feature of these lncRNAs is that they often require stoichiometric interaction with their target molecules to exert measurable regulatory effects. Therefore, it is essential to carefully quantify the cellular copy number of lncRNAs and their target(s) and understand whether an lncRNA is competent to regulate many target genes or whether it is functioning by sequestering some specific abundant proteins. This necessitates the importance of maintaining a defined stoichiometry between the noncoding RNA molecules and their target RNAs or proteins [57]. Yet, it is unclear as to how noncoding RNAs interact with their targets or as to how their expression influences cellular signalling. Although many studies have attempted to predict the putative mechanism of dysfunctional lncRNAs by using the bioinformatics approach, the lack of conserved sequences and the absence of functional motifs have posed complexity in defining their functions—especially so when the secondary or tertiary structures of lncRNAs play significant roles in their biological activity [58].

Ginir belongs to a small group of lincRNAs operating as relatively short-length RNA, in this case having only 612 nucleotides. The Ginir sequence is conserved in rat, implying that the sequence serves some essential function(s). The 557-nucleotides-long sequence isolated initially was later shown to be a subset of the 612-nucleotides-long Ginir RNA sequence, demonstrating that the 557-nucleotide sequence had all the information that was necessary and enough to induce oncogenic transformation in the cells. We used a 3-fold approach—namely, nuclease protection assay, strand-specific primer-mediated reverse transcription followed by PCR, and hybridisation in situ with fluorescently labelled LNA probes to establish that Ginir was transcribed in normal cultured cells as well as during early mouse embryogenesis. Its expression during early development indicated that Ginir transcription was required for normal cellular functions. While examining the relative abundance of Ginir transcripts in normal cultured cells and in tissues during early mouse development, we detected the presence of a complete antisense transcript to Ginir RNA in all of them. Because of its full antisense nature, we designated it as Giniras, which was also a noncoding RNA. During these experiments, we discovered that Ginir and Giniras lncRNAs existed as a pair of sense–antisense overlapping transcripts and that Giniras acted as a NAT to Ginir noncoding RNA. Several NATs are reported in mammals in accordance with the computational studies, which suggest that 15%–25% of mammalian genes are overlapping [59]. Several antisense transcripts to protein-coding genes like antisense intronic noncoding RASSF1 (ANRASSF1) [60], antisense noncoding RNA in the INK4 locus (ANRIL) [61], keratin type II cytoskeleton 7 antisense RNA 1 (KRT7-AS) [62], or Sirtuin 1 antisense RNA (Sirt1-AS) [63] exist wherein they establish complex configurations as RNA–DNA duplexes and triplexes and thereby associate with regulatory proteins to affect the regulation of neighbouring regions. These regulatory mechanisms operate at pretranscriptional and transcriptional levels. A much less studied category is the class of natural double-stranded RNAs (ndsRNAs) that are expressed from interspersed genomic locations. Our data indicate that the Ginir/Giniras noncoding RNA pair is a validated example of ndsRNA. From a 500-Kb region located in chromosome 8, several hundreds of sense–antisense pairs are known to arise. For example, one pair from this region is shown to be expressed from chromosome 8q24.21, and it forms a stable ndsRNA molecule (nds-2a) that binds to regulator of chromosome condensation 1 (RCC1) and ras-related nuclear protein (RAN) and through the latter, with the mitotic RANGAP1-SUMO1-RANBP2 complex [64]. A similar phenomenon is reported in chick embryo development, wherein the stoichiometry in the expression of lncRNA male hypermethylated (MHM), and its antisense RNA is shown to be important for development of organs like gonads, limbs, heart, branchial arch, and brain [65]. With a major number of noncoding RNAs not yet identified, it is possible a vast repertoire of ndsRNA exist, many of which could also be involved in regulating embryonic development and cell growth.

We provide evidence that the Ginir/Giniras pair exhibits contrasting effects on cell growth, like the other noncoding RNA pair X-inactive specific transcript (Xist)/Tsix that has opposing effects on X chromosome inactivation [66]. This is like another lncRNA, ubiquitin carboxy-terminal hydrolase L1 (Uchl1), whose antisense Uchl1-AS expression is dependent on the embedded short interspersed nuclear element (SINEB2) (in its genomic loci) [67]. Instead, a natural antisense RNA to MALAT1 is known to exhibit a feed-forward positive regulatory loop to MALAT1 by promoting its 3′ end cleavage and transcript maturation [68]. Moreover, like the majority of lncRNAs, Ginir is also transcribed from a deserted region of the genome. Although these regions are largely considered as generating transcriptional noise, it is now known that these regions are rich in repeat elements, and they actively produce noncoding transcripts [69]. Although the factors governing evolution and the origin of noncoding RNAs are enigmatic, one possible factor driving lincRNA evolution and their biological functions is transposable element (TE) insertions [70]. A significant fraction of lincRNAs, almost to an extent of 83% in humans and about 66% in mouse, contain an inserted TE [71]. Rather, most of the mammalian genomes that include mouse and human are comprised of repetitive sequences such as TEs, tandem repeats (TRs), and local repeats (LRs) [70]. The locus for noncoding RNA FIRRE, which is required for pluripotency and adipogenesis, on the X chromosome is comprised of numerous LRs [72]. Analysis of the genomic locus of Ginir using genome browsers has revealed that the Ginir locus resides in the LINE1 region. It is recently shown that almost 50% of noncoding RNAs originate near long interspersed nuclear elements (LINE/L1) or SINE/Alu, and fewer than 15% originate in long terminal repeat (LTR)/endogenous retroviruses (ERVs) [73]. This is a minor disagreement from a report that has demonstrated significant enrichment of LTR/ERVs and depletion of LINE L1 and SINE/Alu at the start site of all lncRNAs [71]. It is considered that LINE/L1 sequences are enriched in brain-specific transcripts as compared with non-tissue-specific transcripts [73], yet a statistical evaluation is lacking, and the data mining and analyses from different data sets for low-expression transcripts show variability. Hence, a detailed study is warranted about the presence of these repeats in noncoding RNAs. The fact that Ginir and Giniras are mainly expressed during embryonic development, are found enriched in the developing brain, and are present in other adult tissues (prominently Giniras is expressed) makes any kind of generalisation about a tissue-specific expression pattern or developmental presence of LINE1-encoded lncRNAs difficult. A new class of lncRNAs, termed chromatin-enriched RNAs (cheRNAs), that are tightly associated with chromatin is identified, and their presence is strongly correlated with expression of nearby genes. One of the members of this class of cheRNA is hemin-induced cheRNA downstream of foetal haemoglobin (HIDALGO), which is involved in erythroid differentiation. Werner and colleagues showed that these cheRNAs reside within class I TEs and are cell type specific and play important roles in lineage specification and differentiation [74].

We found that during early embryonic development, Ginir transcripts were more abundant than the antisense Giniras transcripts. As the development progresses and organ development and differentiated tissues become more prominent, an up-regulation of Giniras expression is evident. Thus, it is plausible that a fine balance between the expression of Ginir and Giniras lncRNAs may be amongst the important molecular events during tissue morphogenesis in mouse embryos. LncRNAs like XIST [75], H19 [76], HOTAIR [77], and regulator of reprogramming (RoR) [78] are described as vital players during embryonic development, but most of these RNAs are shown to be crucial during initial stages of development. XIST and H19 regulate processes like X chromosome inactivation and genomic imprinting, whereas HOTAIR and ROR affect the epigenetic changes by interacting with PRC2 complex and thereby determine embryonic stem cell fates. Except for some preliminary reports about lncRNAs like UCA1 [79] and some recently reported ones like Pnky [80], FOXF1-adjacent noncoding developmental regulatory RNA (Fendrr) [81], and AB063319 [82], there are not many reports exemplifying the role of lncRNAs during tissue morphogenesis. One of the lncRNAs, termed lnc myogenic differentiation (MyoD), is encoded next to the MyoD gene and is directly activated by MyoD, thereby inhibiting proliferation and creating a permissive state for skeletal muscle differentiation [83]. Since the pace of cell division decreases and the state of differentiation increases during the later stages of embryonic development, we speculate that Ginir RNA modulates the pace of cell proliferation during development. Since Giniras is complementary to the entire length of Ginir, it can eclipse the entire sequence of Ginir by forming an RNA: RNA hybrid that thereby masks the functioning of important regulatory elements required for RNA: RNA or RNA: Protein interactions. More than 20 noncoding RNAs are linked to cancer. These include but are not restricted to lncRNAs like HOTAIR [84], Pvt1 [85], RoR [86], prostate cancer–associated transcript 1 (PCAT1) [87], ANRIL [88], H19 [89], nuclear enriched abundant transcript 1 (NEAT1) [90], UCA1 [91], lung adenocarcinoma transcript 1 (LUADT1) [92], colorectal cancer–associated lncRNA (CCAL) [93], and many more. Certain lncRNAs like HOTAIR [30], MALAT1 [94], and UCA1 are implicated in metastasis. Here, the involvement of most of them is more of an association than a cause of malignant transformation. For example, genomic loci encoding some of these lncRNAs are hotspots of epigenomic alterations, mutations, single-nucleotide polymorphisms, and somatic copy-number alterations, and thereby their expression levels are found enhanced in cancer. Expressions of some of these lncRNAs are indicators of good or bad prognostic factors, determinants of metastasis, and predictors of therapeutic responsiveness. However, Ginir RNA is distinct from all of them in that it is one of the unique lincRNAs that we unequivocally demonstrate to function singularly as an acutely transforming oncogene. Using several in vitro and in vivo assays, we have demonstrated that Ginir at high expression levels is tumourigenic and effectively potentiates migration, invasion, and metastasis and is also proangiogenic. Analyses of Ginir transcript abundance in oncogenically transformed cells show that it is more abundant in pathological cells compared to normal cells. Down-regulation of Ginir function by expressing Giniras RNA or using sequence specific shRNAs causes significant attenuation of tumourigenic potential of Ginir RNA–expressing cells.

To gain an insight into the mechanistic details of oncogenic transformation brought about by Ginir RNA, we created specific cell lines in which Ginir, Giniras, and Ginir+Giniras transcripts were expressed in abundance, using exogenously introduced transcript constructs. We also created cell lines in which a relatively high level of Ginir RNA was being expressed initially, which was subsequently brought down by coexpressing shRNAs that were specific to Ginir RNA sequences. The results from all these experiments are in accord with a model in which an excess of Ginir RNA was shown to be involved in deregulating a checkpoint that was involved in suppressing cellular proliferation or that was prolonging the cell cycle duration or both (Fig 12).

Our data indicated that when the level of Ginir expression in the tumour cells was down-regulated, or Ginir RNA was sequestered by its antisense partner Giniras, the cells apparently exited the transformed state in that they were no longer able to form credible tumours in mice. A coordinated loss of a set of related properties was indicative of a primary role that the Ginir overexpression had in orchestrating them. Because we detected the differential regulation of Ginir and Giniras transcription, our hypothesis that Ginir promoted proliferation, whereas Giniras retarded proliferation and promoted differentiation as a part of natural regulatory mechanism, becomes supported. Ginir-overexpressing cells showed a higher number of multinucleated giant cells having copious Ginir expression within nuclei. These types of giant cells are also formed during senescence, but they are not always multinucleated, and they are nonproliferative. The giant cells induced by Ginir, by contrast, were proliferative because mitotic and proliferative capacity was present in them, indicating that the nuclear divisions had been desynchronised from the cytokinesis. Thus, a specific property associated with Ginir-transformed tumour cells was that the mitotic dynamics persisted in the absence of cytokinesis, giving rise to giant multinucleated cells [95]. Such polyploid giant cells have also been cited as a repertoire of cancer stem cells and are likely responsible for their continuous generation [96].

In some cases of lncRNAs, an epigenomic alteration results from its nuclear localisation. We have demonstrated here that the multinucleated giant cells have an overabundance of Ginir RNA in their nuclei (more than the NIH/3T3-Ginir nuclei), suggesting that there may have been reprogramming of its epigenome. Thus, the giant multinucleated cells may represent cells qualified for dedifferentiation and most appropriate to acquire stem cell–like properties, which are hallmarks of high-grade tumours that were observable in the tumours induced by NIH/3T3-Ginir cells growing as xenografts in mice.

Our studies show that knocking down endogenous Ginir in normal cells diminished their proliferation. The mouse melanoma cells like B16F10, when made deficient for Ginir RNA, led to attenuation in their transformation-related properties in vitro and to tumourigenicity in vivo. These results underscore that Ginir and Giniras RNAs must be included in those control mechanisms for which proliferation needs to be modulated, and accordingly, the Ginir RNA and its negative modulator Giniras RNA expression during embryonic growth are coordinated. Mutations including deletions or genomic sequence alterations in Ginir or Giniras locus may prove to be embryonic lethal, precluding their isolation to delineate individual functions of these two RNAs using a genetic approach.

Our finding of an increased level of γH2Ax foci representing DNA double-stranded breaks being present in the cells with higher levels of Ginir RNA is the fallout of a compromised DDR pathway. A strong oncogenic stimulation apparently forces a cell to undertake a proliferation cycle without adequate preparation, thereby having increased episodes of replication fork stalling or replication fork collapse. Since single-stranded DNA and unreplicated ends accumulate beyond an accepted time delay, the surveillance machinery comprising the checkpoint kinases sense them as dsDNA breaks and activate DDR responses. To make this repair process efficient and error free, BRCA1 protein function is critically required. DNA damage after ionising radiation treatment results in accumulation of γH2Ax foci, which also activates the repair response, but an important difference is that growth arrest sets in immediately after the DNA damage. In the case of Ginir RNA overexpression, however, the γH2Ax foci accumulate in the nuclei without the onset of growth arrest. BRCA1 protein has multiple roles in cell physiology, and it interacts with a variety of proteins and noncoding RNAs in both cytoplasmic and nuclear compartments. During cell division, it performs a centriolar role in collaboration with the centrosome proteins like the Cep proteins. These proteins have been identified as components of the centrosome complex using a proteomics approach [97]. One of the members of the Cep family, Cep112, is demonstrated by us as a strong interactor of Ginir RNA. Our data demonstrate clearly that Brca1 stabilises the Cep112 protein by directly interacting with it and localising it to the centrosomal compartment. In the presence of an overabundant supply of Ginir RNA, both Brca1 and Cep112 proteins remain bound to the Ginir RNA and consequently Brca1:Cep112 protein–protein interaction is impaired. The disruption of this interaction also mislocalises the Cep112 and Brca1 proteins into different cellular compartments, thereby inhibiting their natural functions. We have provided convincing evidence in support of direct Cep112–Brca1 protein interaction, and further, we have demonstrated that interaction of Ginir RNA with Cep112 and Brca1 proteins deregulates centrosome functions and disrupts spindle assembly, thereby impairing mitotic regulation. Together, our data show that these processes promote defects in centrosome and spindle functions that include a desynchronised karyokinesis from cytokinesis, giving rise to multinucleated giant cells. All these effects together contribute to an increased genomic instability. Indeed, Brca1 is also known to interact with another lncRNA—DNA damage–sensitive RNA1 (DDSR1)—that is induced after DNA damage, wherein it promotes homologous recombination by regulating recruitment of DNA repair factors to double-stranded breaks after DNA damage [98]. Several other lncRNAs like the recently identified NORAD [56], another lncRNA-JADE, an activator of Jade1 [99], and RoR [100] are critically involved in maintenance of genomic stability. Brca1, when recruited to centrosome, interacts with a plethora of centrosomal proteins like γ-tubulin [51], Nlp [50], OLA1 [49], and KIAA0101 [101], ensuring error-free centrosomal duplication and cytokinesis. Most importantly, a recent finding demonstrates binding of a Cep family protein (Cep72) to Brca1, thus disturbing centrosomal regulation by Brca1 and consequently triggering genomic instability [102]. According to a recent report, Brca2 functions with Cep55 to regulate cytokinesis and ploidy [103]. Recently, another member of the Cep family, Cep192, is shown to be important in the interphase-organising microtubules and cytoskeleton. Defects in targeting Cep192 for proteasome-mediated degradation by a member of the Fbxo family, F-box only protein 13 (Fbx13), was shown to cause accumulation of CEP192 and ɣ-tubulin at the centrosomes with the consequence of defects in cell motility [104].

Here, we provide data on a novel interaction between Cep112 and Brca1 in NIH/3T3 cells, which is abrogated upon Ginir overexpression. Although interaction of Brca1 protein to other centrosomal proteins is known, this is a first report, to our knowledge, demonstrating interaction of Brca1 protein to a novel centrosomal protein Cep112. This interaction is important for stabilisation of the interacting proteins as revealed by our data (Fig 9I), and thus, any disruption in their interaction causes a decrease in the abundance of these proteins. Additionally, a high level of expression of Ginir RNA apparently affects the intracellular localisation of these proteins, thereby placing them away from their normal functional sites. We found that Cep112, which is a cytoplasmic protein with prominent localisation in the centrosome, was enriched in the nucleus in response to an elevated expression of Ginir RNA. The nuclear localisation of Cep112 could be an attribute of a decoying function of Ginir. In these cells, Brca1 protein was down-regulated and was nearly absent from the nucleus with a much-diminished presence in the centrosome in Ginir RNA–induced transformed cells. Thus, mislocalisation of these two proteins from their functional sites caused mitotic dysregulation and thereby served as crucial contributor to genomic instability.

Taken together, Ginir may be considered as an oncofoetal molecule like H19, as it shares a similar expression pattern with it during mouse embryonic development followed by its activation during adult tumourigenesis [105]. While our study establishes the effects of disequilibrium between Ginir/Giniras RNAs in cell growth regulation, still several questions remain unanswered, like the physiological roles of Ginir/Giniras RNAs during development. Undoubtedly, a detailed understanding of their biology would have tremendous ramifications in investigating the role of lncRNAs in fine-tuning gene expression and regulating tissue homeostasis. This could pave the way for designing novel therapeutic strategies for proliferative disorders like cancer. A detailed understanding of Ginir–Giniras equilibrium could open avenues for utilisation of NATs in disease, therapy, and molecular research.

## Materials and methods

### Ethics statement

The use of animals for this study was approved by the Institutional Animal Ethics Committee (IAEC) of National Centre for Cell Science (NCCS); Pune, India (IAEC/2016/B-263). All animal procedures followed were strictly in accordance with the animal ethics guidelines of the NCCS.

### Cell culture

All cells were obtained from American Type Culture Collection (ATCC; Manassas, VA, United States). Mouse fibroblast cells NIH/3T3 were maintained in Dulbecco’s Modified Eagle Medium (DMEM; Invitrogen; Carlsbad, CA, US) supplemented with 10% bovine calf serum (Invitrogen). Clone M3, mouse melanoma cells were maintained in Ham’s F10 medium supplemented with 15% horse serum (Invitrogen) and 2.5% foetal bovine serum (FBS; Invitrogen). B16F10, mouse melanoma cells were maintained in DMEM (Invitrogen) supplemented with 10% FBS (Invitrogen). All the cell lines were maintained in culture in the presence of antibiotics penicillin (200 U/ml) and streptomycin (200 μg/ml) (Sigma-Aldrich, St. Louis, MO, US). The cell cultures were incubated at 37 °C with 5% CO_2_ in a humidified incubator. All cell lines are tested for mycoplasma contamination regularly.

### RNA isolation, strand-specific cDNA preparation, and RT-PCR

RNA extraction from cell lines was performed using TRIzol RNA Isolation Reagent (Invitrogen) by following the manufacturer’s instructions. The RNA was treated with RQ1 Rnase-free DNase (1 U/μl, Promega, Madison, WI, US, # M6101) for 30 minutes at 37 °C. cDNA was prepared with 1 μg of RNA using oligo-dT and random primers, whereas orientation-specific cDNA was prepared with 3 μg of RNA using Ginir- (sense; G1F) or Giniras- (antisense; G1R) specific primers along with primers for internal control Gapdh at 42 °C for 90 minutes using a Reverse Transcription System kit (Promega, # A3500).

Semiquantitative PCR was performed using Taq DNA polymerase (Merck Bioscience; Darmstadt, Germany). Strand-specific real-time PCR was performed using Mesa green master mix (Eurogenetec, Seraing, Belgium) using ABI 7500 Fast real-time PCR (Applied Biosystems, Foster City, CA, US). Primers used for the experiments are listed in S3 Table.

### Transfections with plasmid DNA, siRNA, or shRNA

Transfections were done using Lipofectamine 2000 reagent (Thermo Fisher Scientific, Waltham, MA, US, # 12566014) according to the manufacturer’s instructions. NIH/3T3 cells were transfected with pTargetT expression vector (Promega) constructs of either Ginir or Giniras cDNAs, and the stable transfectant clones were selected using G418 (500 μg/ml, Thermo Fisher Scientific, # 10131035). Cells transfected with only pTargetT expression vector served as control. Additionally, stable clones coexpressing both Ginir (cloned in pTargetT and Giniras (cloned in pCEP4; Thermo Fisher Scientific) were generated in NIH/3T3 cells and selected on both G418 (500 μg/ml) and Hygromycin (100 μg/ml) (Thermo Fisher Scientific, #10687–010). The overexpression of Ginir and Giniras was determined by strand-specific PCR as described earlier. Only those clones were selected that showed Ginir and Giniras overexpression by ≥2 fold and were propagated as independent cell lines. We performed these transfections thrice, and from each transfection, multiple clones were chosen. We here report detailed characterisation of three independent transfectant clones A, B, and C, and each experiment was performed using at least two independent transfectants at least thrice.

siRNA transfections were done in NIH/3T3 cells with two independent siRNA pools of Brca1 and Cep112. Pool # 1 siRNAs were procured from Santa Cruz, Dallas, TX, US, and pool # 2 siRNAs (Stealth siRNAs) were purchased from Thermo Fisher Scientific. For pool # 1 siRNA transfections, 100 nM each of Brca1 siRNA pool (m) (Santa Cruz, #sc-29824), Cep112/Ccdc46 siRNA pool (m) (Santa Cruz, #sc-142115), and control siRNA-A (Santa Cruz, #sc-37007) was used. Cells were assayed after 48 hours of transfection. For pool # 2 transfections, 100 pmol of each of Brca1 siRNA pool (m) (Thermo Fisher Scientific, #1320001, ID- MSS202430), Cep112 siRNA pool (m) (Thermo Fisher Scientific, #1320001, ID- MSS293588), and control stealth siRNA was used in 2 ml of complete medium. NIH/3T3-Ginir cells were transfected with shRNAs for Ginir and scrambled shRNAs as control (TransOMIC technologies, Huntsville, AL, US). The transOMIC shRNAs used in this study were designed using Custom shERWOOD Design Service. The sequences for shRNAs (1 and 2) and control shRNA used are as follows.

#### shRNA#1

TGCTGTTGACAGTGAGCGCAGCCAGAATTGTTCCTGTATATAGTGAAGCCACAGATGTATATACAGGAACAATTCTGGCTTTGCCTACTGCCTCGGAshRNA

#### shRNA#2

TGCTGTTGACAGTGAGCGCAGCAAGATTCTGACCTAAAATTAGTGAAGCCACAGATGTAATTTTAGGTCAGAATCTTGCTTTGCCTACTGCCTCGGAshRNA

#### Nontargeting control

TGCTGTTGACAGTGAGCGCCCGGCTGAAGAGCCTGATCAATAGTGAAGCCACAGATGTATTGATCAGGCTCTTCAGCCGGTTGCCTACTGCCTCGGA

### Lentiviral preparation and transduction

HEK293T cells grown to 70%–80% confluency in a 60-mm dish were transfected with Lipofectamine 3000 (Invitrogen) using the manufacturer’s instructions. Briefly, in two separate tubes—one having 8 μl of Lipofectamine 3000 mixed with 250 μl of Opti-MEM and another with 5 μg of plasmid DNA constructs (i.e., packaging plasmid pCMV delta R8.2 [2 μg], envelope plasmid pCMV-VSV-G [1 μg], and transfer plasmid pZIP-shGinir [1 and 2] [2 μg]) in 250 μl of Opti-MEM and 10 μl of P3000 Reagent—were incubated at room temperature (RT) for 5 minutes. Later, the contents of both the tubes were mixed and further incubated at RT for 30 minutes to form liposome–DNA complexes. Meanwhile, cells were rinsed in plain DMEM and fed with 3 ml of DMEM. The DNA–liposome mix was added dropwise onto the cells, incubated at 37 °C for 6 hours, and fed with complete medium. After 12 hours, the culture medium was replaced with DMEM-F12 medium supplemented with sodium butyrate and incubated at 37 °C for 6 hours. Next, complete media containing high serum (20% FBS) were added to the plate, and cells were further incubated for 24 hours at 37 °C. Later, viral supernatant was collected in a 15-ml tube, and again, fresh media containing high serum (20% FBS) were added. Next day, both the 24-hour and 48-hour viral supernatants are pooled and spun at 2,000 rpm at 4 °C for 10 minutes. Pooled viral supplements were used for transduction of target cells. After 48 hours, the cells were selected on puromycin- (1 μg/ml) containing medium for 7–8 days by intermittently replacing them with fresh medium. The puromycin-resistant colonies were pooled and subcultured to develop into cell lines. These were maintained on complete medium supplemented with penicillin and streptomycin and puromycin (1 μg/ml). The stable cell lines were cryopreserved in liquid nitrogen.

### Rapid amplification of cDNA Ends (RACE)

Total mRNA was isolated using Oligotex direct mRNA isolation kit (Qiagen, Hilden, Germany) using manufacturer instructions. RACE was performed using Marathon RACE cDNA amplification kit (Clontech, Mountain View, CA, US) according to the manufacturer’s protocol. The Marathon adapters were ligated using T4 DNA ligase. These adapter-ligated RACE cDNAs were used for primary and nested PCR using Ginir-specific primers and adapter primers (S3 Table). RACE-PCR reactions were performed using Advantage 2 Polymerase Mix (Clontech). The primer sequences used were as follows:

#### Gene-specific primers (GSPs) for 3′ RACE

GSP1—GGATATCCTAACACACCTGAAAAGC. Nested GSP2—CTGCATCTGTCTTAGGTGCTGG.

#### GSPs for 5′ RACE

GSP1—GTTGTAAACCCCTTCAGCTCCTGC. Nested GSP2—GATGTCAGGGATGTGAGAGGTTC.

The PCR products were vacuum blotted onto Amersham Hybond N+ membrane (GE Healthcare Life Sciences, Pittsburgh, PA, US), and Southern hybridisation was performed with a Ginir-specific labelled DNA probe. The probe was generated by labelling during PCR for Ginir amplification using 50 μCi of γP32 dCTP. According to the signal obtained, the corresponding band was gel eluted, cloned into pGEM-T vector (Promega), and sequenced. The sequences were analysed using various bioinformatics tools like BLAST.

### Ribonuclease protection assay

Ribonuclease protection assay was performed using HybSpeed RPA kit (Ambion, # 1412) as per the manufacturer’s protocol. Total RNA was isolated from cells and treated with DNase I to remove the traces of DNA contamination. Fifty μg of DNased total RNA and 5 × 10^6^ CPM of Ginir-specific (sense/antisense) probes (1 × 10^5^ CPM/μg of RNA) were mixed and coprecipitated with ammonium acetate and ethanol. The pellet was dissolved in 20 μl of HybSpeed hybridisation buffer. The reaction mix was denatured at 95 °C for 5 minutes, hybridised for 3 hours at 68 °C with sense/antisense riboprobes for Ginir, and digested using RNaseA/T1 mix for 30 minutes at 37 °C. The RNA was dissolved in 10 μl RNA gel loading buffer, denatured at 90 °C for 5 minutes, and separated on 10% PAGE with 8 M Urea. The gel was wrapped in Saran wrap and exposed to phosphor screen overnight and scanned using phosphor imager FX (Biorad, Hercules, CA, US).

### Embryo isolation and expression analysis

Pregnant female Swiss Webster (CFW) females (4–6 weeks old) were used for embryo isolation. Embryos ranging from 5.5 dpc to 13.5 dpc were isolated by dissecting impregnated mice using dissection microscope and instruments. These embryos were very carefully washed with 1X phosphate-buffered saline (PBS). Half the embryos were fixed in 4% paraformaldehyde (PFA) for in situ hybridisation, and the remaining embryos were stored in TRIzol at −80 °C until use. Morphological criteria were used to identify precise embryo staging using ‘Theiler Staging Criteria for Mouse Embryonic Development’.

RNA was isolated from embryos of all the stages using TRIzol reagent (Invitrogen) following the manufacturer’s protocol. Strand-specific cDNA for Ginir/Giniras and Gapdh was prepared using Quantitect Reverse Transcription Kit (Qiagen, # 205311).

### RNA-FISH

RNA-FISH was performed on cells and embryo sections using custom-designed Ginir- and Giniras-specific fluorescent LNA probes (Exiquon, Vedbaek, Denmark). Two independent LNA probes targeting different regions of Ginir sequence were used to ensure specificity of the fluorescence. The sequences of LNA probes used were the following:

Ginir-Probe 1 (Green)—/56-FAM/GGTGGCCTTTCCTTCAGTCTCT
Ginir-Probe 1 (Red)—/5TEX615/GGTGGCCTTTCCTTCAGTCTCT
Ginir-Probe 2 (Red)—/5TEX615/ACATCTGAGACTTTCTTGT
Giniras-Probe (Red)—/5TEX615/CATCCAGTAGATAGTCACAGCC

In brief, cells and embryos at various dpc stages, as well as whole-mount embryos, were fixed in 4% PFA followed by permeabilisation in 0.5% Triton-X 100 containing RNasin (50 U/ul, Thermo Fisher Scientific, #AM2694). Prehybridisation was done at 37 °C for 30 minutes in hybridisation buffer without probe (10% dextran sulphate, 0.5% BSA, 2X SSC in 50% formamide, RNasin 50 U/μl). Simultaneously, cells used for RNase control were treated with RNase A (1 mg/ml, Sigma, # R4642) in 1X PBS at 37 °C followed by prehybridisation at 37 °C for 30 minutes. Denaturation of LNA probes (25 nM/ sample) was done at 80 °C for 75 seconds in hybridisation buffer containing 500 μg/ml of tRNA and 50 U/μl of RNasin. Hybridisation was performed by inverting the cover slips on paraffin-coated slides and incubating them at 46 °C in a moist hybridisation chamber for 4 hours. Coverslips were then transferred to a 24-well plate for washes—first wash (50% formamide in 2X SSC + RNasin 50 U/μl) for 20 minutes at 37 °C, second wash (2X SSC + RNasin—50 U/μl) for 20 minutes at 37 °C, third wash (1X SSC + RNasin—50 U/μl) for 20 minutes at RT, and final wash (4X SSC) for 2 minutes at RT. Nuclei were stained with DAPI and mounted using mounting medium supplemented with DABCO. Confocal images were collected using Zeiss LSM510 META confocal microscope with an Axiovert 100M imaging system (Carl Zeiss, Oberkochen, Germany). A few of the images were acquired using Leica SP5 II system (Leica Microsystems, Wetzlar, Germany) and with Olympus FluoView FV1000Confocal Microscope (Olympus, Shinjuku, Tokyo, Japan).

### Cell proliferation assay

Proliferation potential of cells was determined by using an MTT assay [106,107]. Briefly, cells were seeded into 96-well plates (BD Biosciences, San Jose, CA, US) at a cell density of 1 × 10^3^ cells/well in growth medium. Cell growth was assayed by addition of 20 μl of MTT (5 mg/ml; Sigma-Aldrich) to each well, and the plate was incubated at 37 °C for 4 hours. The proliferation assay was performed for 3–7 days, and cell growth was assayed at every 24-hour interval. Later, the reaction was stopped by addition of 200 μl dimethyl sulfoxide (Sigma-Aldrich). Optical density was measured at 570 nm with a microplate reader (Bio-Rad, Hercules, CA, US).

### Soft agar assay

The clonogenic potential of cells was assessed by soft agar assay by a method described by Anzaono and colleagues [108,109]. The assay plates were incubated for 7–10 days at 37 °C to score for colony formation. Each set was plated in triplicates, and the assay was performed at least three times. The colonies were counted under Olympus IX70 inverted microscope using 10× objectives in 10 different fields to acquire the average number of colonies per cell line.

### Wound healing assay

Migration of cells in vitro was determined by wound-closure migration assay [110]. A single wound was created in the centre of cell monolayers and later visualised after 6–20 hours (less than doubling time of the cells) to detect migration of cells to the created gap. Three independent experiments were performed, and data represent the average ± SEM of the wound gap that remained after 6–20 hours.

### Comet assay

Alkaline Comet assay was performed as described by Olive and colleagues (95). Comet slides were stained with PI at a concentration of 20 μg/ml for 10 minutes. Images were acquired on Leica SP5 II system (Leica Microsystems).

### CAM assay

The CAM assay for assessing angiogenesis was performed on a patch of chorioallantois exposed by the shell window of fertilised white chicken eggs as per the method described [111,112].

### In vivo tumour formation

For in vivo tumourigenicity assays, female NOD-SCID mice were used. Briefly, 1 × 10^6^ of test and control cells were injected subcutaneously into NOD-SCID mice, and mice were periodically observed for tumour development. Tumour volumes were determined using the formula 1/2(Length × Width^2^). Tumours were formalin fixed and analysed by HE staining. Also, different tissues like lungs were sectioned and stained with HE to analyse the extent of invasion of injected cells.

### In vivo tail vein metastasis assay

Control NIH/3T3-EV, NIH/3T3-Giniras, and NIH/3T3-Ginir transfectant clones A and B were injected into NOD/SCID mice via tail vein with 1 × 10^6^ cells. The mice were killed at 6 weeks and 8 weeks post injection. Later, the lungs were sectioned and stained with HE for histopathological analysis.

### Nuclear and cytoplasmic RNA isolation

Cytoplasmic and nuclear fractions from cells were collected using Nuclear Extraction Kit (Chemicon International, Billerica, MA, US, # 2900) according to the manufacturer’s instructions. Briefly, cells were harvested and resuspended in 5 cell pellet volumes of ice-cold 1X Cytoplasmic Lysis Buffer containing RNasin (100 U/ml, Thermo Fisher Scientific) followed by incubation on ice for 15 minutes. Cells were then pelleted and resuspended in two volumes of ice-cold 1X Cytoplasmic Lysis Buffer (with RNasin). Cells were lysed in the buffer by continuous drawing and ejecting through a 27-gauge needle. Cell suspension was centrifuged, and a supernatant containing a cytosolic portion of the cell lysate was collected. The remaining pellet containing the nuclear portion of the cell lysate was resuspended in two-thirds of the original cell pellet volume of ice-cold Nuclear Extraction Buffer (with 100 U/ml RNasin). The nuclear portion was similarly disrupted using a 27-gauge needle followed by agitation at 4 °C for 30–60 minutes on a rotor at low speed. The suspension obtained was then centrifuged at 16,000*g* for 5 minutes at 4 °C, and the supernatant containing the nuclear fraction was collected. RNA from each fraction was isolated using Trizol Reagent (Ambion).

### Cell cycle analysis by PI staining

For cell cycle analysis, cells were fixed in chilled methanol at −20 °C for 10 minutes, rehydrated in PBS for 30 minutes, and treated with RNase A (100 μg/ml, Sigma-Aldrich) at 37 °C for 30 minutes. Nuclei were stained with PI (Invitrogen, # P1304MP) for 30 minutes. A total of 10,000 nuclei were examined by flow cytometry using FACS Calibur (BD Biosciences, San Jose, CA, US), and DNA histograms were analysed using CellQuest Pro FACS analysis software 5.2.1 (BD Biosciences).

### Western blotting

Cells were lysed in 1X M-PER Mammalian Protein Extraction Reagent (Pierce, Rockford, IL, US) containing 1X protease inhibitor cocktail (PIC) (Sigma-Aldrich). The lysates were separated on SDS-PAGE and transferred to Hybond-P PVDF Membrane (GE Healthcare) followed by immunoblotting and detection with Super Signaling West Femto Kit (Pierce). Primary antibodies used for immunoblotting were Brca1 (1:2,000, # sc-7867, # sc-646), Cep112 (1:1,500, # sc-246162, # sc-246163), Gapdh (1:2,000, # sc-32233), β-actin (1:2,000, # sc-81178), PCNA (1:1,000, sc-7907), pp21 (Ser146) (1:1,000, # sc-12902), and pRb (1:1,000, # sc-12901R) from Santa Cruz Biotechnology; α-tubulin (1:10,000, # T8203) and RPA32 (1:1,000, # R1280) from Sigma Aldrich; Phospho-Histone H2A.X (Ser139)/γH2Ax (1:4,000, # 2577S), pp53 (Ser15) (1:1,000, 9284S), and Flag (1:1,000, # 2638) from Cell Signalling (Danvers, MA, US); and Cep112 (1:2,000, # 24928-1-AP) and Brca1(1:2,000, # 20649-1-AP) from Proteintech (Rosemont, IL, US). The HRP-conjugated secondary antibodies were Goat anti-Mouse IgG HRP (1:2,000, # 31430), Goat anti-Rabbit IgG HRP (1:10,000, # 31463), Rabbit anti-Goat IgG (1:2,000, # 31433) from Pierce, and Donkey anti-Goat HRP (1:6,000, Santa Cruz, # sc-2020). Quantification of western blots was done using Image J tool, version 1.41.

### Immunofluorescence microscopy

Cells were grown onto coverslips for 24–48 hours. Later, they were fixed in 4% PFA (Sigma-Aldrich) for 10 minutes at RT. For immunostaining of centrosomal proteins, cells were fixed with chilled methanol for 20 minutes at −20 °C. Later, cells were washed with 1X PBS, permeabilised using 0.01% Triton X-100 (Sigma-Aldrich) for 10 minutes, and blocked with 5% BSA (MP Biomedicals, Santa Ana, CA, US) for 30 minutes at RT. This was followed by incubation with primary antibodies Ki67 (1:100, # sc-23900), Cep112 (1:50, # sc-246163), Brca1 (1:100, # sc-646), Ark1 (1:50, # sc-14321), and PCNA (1:100, # sc-7907) from Santa Cruz Biotechnology; Cep112 (1:100, # 24928-1-AP) and γ-tubulin (1:500, # 15176-1-AP) from Proteintech; γ-tubulin (1:500, # T5326) and α-tubulin (1:1,500, # T8203) from Sigma-Aldrich; 53BP1 (1:100, # 4937), Mre11 (1:100, # 4895), RAD-52 (1:100, # 3425), ATMATR-S (1:100, 2851S), pATM (Ser1981) (1:100, # 4526S), p53 (1:100, # 2524S), and γH2Ax (1:500, # 2577S) from Cell Signalling; and p21 (1:100, Abcam, Cambridge, UK, # 18209). All antibodies were diluted in 1X PBS and incubated for 1 hour at RT. The cells were then incubated with appropriate species-specific Alexa Fluor–conjugated secondary antibodies (Molecular Probes, Invitrogen) diluted in 1X PBS (1:100 dilutions) for 1 hour at RT in dark. Later, cells were incubated with 4′,6-diamidino-2-phenylindole (DAPI) (Sigma-Aldrich) for 10 minutes and mounted in medium containing 1% 1,4-diazabicyclo (2.2.2) octane (DABCO) (Sigma-Aldrich). Confocal images were acquired using Zeiss LSM510 META confocal microscope with an Axiovert 100M imaging system. A few of the images were also acquired using Leica SP5 II system and Olympus Fluoview FV1000 Confocal Microscope.

### Quantification of centrosomes

The centrosome content was determined using the method described by Starita and colleagues [113]. In brief, the centrosome number was determined for cells in both interphase and metaphase by staining them using ɣ-tubulin-specific antibody followed by Alexa Fluor–conjugated secondary antibody. The cells were visually scored for centrosome content using Leica SP5 II confocal system. At least 150 cells were counted, and cells with normal (1 to 2) and abnormal (>2) centrosomes were individually scored, and a histogram was plotted as the percentage of cells with normal and abnormal centrosome numbers. The experiment was performed at least 3 times for each cell type.

### Biotin RNA pull-down assay

Biotinylated pull-down assay with Ginir/Giniras probes was performed using the method described by McHugh and colleagues [114]. Whole-cell lysates were harvested from 100-mm dishes. Ginir/Giniras specific riboprobes were synthesised using Megascript T7 Kit (Invitrogen) as per the manufacturer’s protocol. For preparing Ginir hot probe, 0.2 mM of biotinylated CTP (Biotin-14-CTP) (Invitrogen, #19519–016) was added to the reaction mix along with the PCR product to be used as the template. In another independent reaction, cold probe was synthesised using one-tenth of Bio-CTP as used in hot probe synthesis. Control unbiotinylated probe was synthesised with no biotin in the reaction mix. Using similar reaction mix, antisense (Giniras) and unrelated (Hotair, XEF) biotin probes were also synthesised using their respective PCR products as a template. All the tubes were incubated at 37 °C for 4 hours. After RNA synthesis, turbo DNase was added to digest template DNA. RNA was precipitated using the ammonium acetate precipitation method. All the probes along with controls were incubated with cell lysates and passed through streptavidin columns (μMACS Streptavidin Kit, Miltenyi Biotec, Bergisch Gladbach, Germany). Hot-bound, cold-bound, and no-biotin-probe-bound elutes were collected and resolved in SDS-PAGE along with the input fraction. The gel was stained with Coomassie Brilliant Blue. The specific protein bands were then purified by in-gel digestion (In gel digestion kit, Thermo Scientific) and analysed by MALDI-TOF. For validation of pull-down, immunoblotting of elutes with specific antibodies for Brca1 (1:2,000, Santa Cruz, # sc-7867) and Cep112 (1:2,000, Proteintech, # 24928-1-AP) was done.

### RIP

Native RIP was performed as described [115]. Briefly, cells were cross-linked using 1% formaldehyde for 30 minutes at RT and then pelleted. Later, cells were lysed using SDS lysis buffer (1% SDS, 10 mM EDTA, 50 mM Tris HCl [pH 8.1]) and RNasin (50 U/ml). Lysates were sonicated at high power for 4 minutes (30 seconds on-and-off cycle). After sonication, the insoluble elements were cleared by centrifugation at maximum speed for 10 minutes at 4 °C. The supernatant was then diluted 10-fold with IP buffer (0.01% SDS, 1.1% Triton X-100, 1.2 mM EDTA, 16.7mM Tris [pH 8.1], 167 mM NaCl, 1X PIC, RNasin [50 U/ml]). Then, 10% of the aliquot was preserved as an input sample and frozen at 80 °C until the reverse cross-linking step. The rest of the lysate was precleared with protein A/G beads and respective Isotype IgG overnight at 4 °C. One-ml aliquots were made of the diluted supernatant, and 0.5 μg of respective antibodies (Brca1, Cep112, and Gapdh) were added to each of the tubes. Respective Isotype IgG was used as negative control. Immune complexes were formed by slow mixing on a rotating platform at 4 °C overnight. Immune complexes were collected by adding 50 μl of protein A/G Agarose beads (Santa Cruz) followed by mixing by rotation at 4 °C for 2 hours. The beads were preblocked with yeast tRNA (Invitrogen, # AM7179) (50 μl of beads with 2 mg tRNA) by rotation mixing for 90 minutes at 4 °C. Immune complexes were then washed for 5 minutes each with low-salt buffer (0.1% SDS, 1% Triton X-100, 2 mM EDTA, 20 mM Tris HCl [pH 8.1], 150 mM NaCl), high-salt buffer (0.1% SDS, 1% Triton X-100, 2 mM EDTA, 20 mM Tris HCl [pH 8.1], 500 mM NaCl), and LiCl buffer (0.25 M LiCl, 1% NP40, 1% deoxycholate, 1 mM EDTA, 10 mM Tris-HCl [pH 8.1]). This was followed by two washes with TE buffer (Tris-EDTA [pH 8.0]). Immune complexes were then eluted in elution buffer (1% SDS, 0.1 M NaHCO_3_, RNasin—50 U/μl). Reverse cross-linking was done with 200 mM of NaCl at 65 °C for 2 hours. RNA isolation was done from elutes and input samples using TRIzol reagent (Invitrogen). Strand-specific cDNAs were prepared after DNase treatment followed by PCR amplification with gene-specific primers.

### Protein coimmunoprecipitation

Cells were lysed in IP lysis buffer (Pierce) containing 1X PIC (Sigma-Aldrich). To evaluate RNA–protein interactions, 500 μg of lysate was treated with RNasin (200 U/ml), whereas the other 500 μg was subjected to treatment with RNase A (200 μg/ml, Sigma, # R4642), RNase III (0.05 U/μl, Invitrogen, #AM2290), and RNase H (5 U/μl, Invitrogen, # 18021–014) at 37 °C for 1 hour. The lysates were further used for immunoprecipitation using Dynabeads protein-A immunoprecipitation kit (Invitrogen, # 10006D), and the manufacturer’s protocol was followed. Two μg of antibodies to Brca1 (Santa Cruz, # sc-646), Cep112 (Santa Cruz, # sc-246163), Flag (Cell Signalling, # 2638), Rabbit IgG (Santa Cruz, # sc-2027), and Goat IgG (Santa Cruz, # sc-2028) were added to 50 μl of protein-A beads in 200 μl of antibody binding and washing buffer and incubated with rotation mixing at 4 °C for 6 hours. Antibodies were cross-linked to protein-A beads by incubating with dimethyl pimelimidate dihydrochloride (DMP) (Sigma, # D8388) in cross-linking buffer (0.2 M Triethanolamine [pH 8.2]) for 1 hour and 30 minutes at RT. Beads cross-linked with antibodies were then incubated with test lysates along with 1X PIC at 4 °C for 2 hours. Three brief washes in washing buffer were followed by elution in low-pH elution buffer (0.1 M glycine [pH 2.5]). The elutes were then run in SDS-PAGE along with input fractions and immunoblotted with respective antibodies.

### Transcriptome analyses by RNA-seq

Total RNA was isolated from NIH/3T3, NIH/3T3-Ginir, NIH/3T3-Giniras, and Clone M3 cells using TRizol reagent according to the manufacturer’s protocol. RNA-seq libraries were generated using the Illumina TruSeq kit (version 2) following the manufacturer’s instructions. The libraries were sequenced on a HiSeq 2000 system (Illumina), and the RNA-seq reads (read length 100 bp) were analysed with Basespace’s RNA Express pipeline (RNA Express Legacy version: 1.0.0), which encompasses alignment using HISAT2 [116]. Quantification and differential analyses were done using String Tie-Ballgown protocol. Identification of GO terms enriched in the genes up-regulated in NIH/3T3-Ginir was performed using online tool DAVID (https://david.ncifcrf.gov/summary.jsp).

### Computational analysis

Noncoding potential of Ginir was validated using prediction tools like test code, CPC program, and phylo CSF. ORF finder was used to predict the probable ORFs in Ginir sequence. Chromosomal localisation and other gene-based predictions in relation to Ginir sequence were carried out using the UCSC genome browser. Computational prediction of Ginir interaction to probable proteins was done with tools like catRAPID and RPIseq. We used Ensembl and Uniport databases to validate Cep112 isoforms. Protein docking analysis for Brca1 and Cep112 was done using ZDOCK tool.

### Statistical analysis

All statistical analyses were performed using Graphpad Prism software. Results are presented as mean ± SEM. We used one-way or two-way ANOVA for comparison between two experimental groups in different experiments, as described in the figure legends. Additional statistical information has also been provided in the figure legends. *P* ≤ 0.05 was considered as statistically significant for all the experiments, and values were assigned accordingly (*P ≤ 0.05, **P ≤ *0*.*001*, ***P ≤ *0*.*0001)*.

## Supporting information

S1 Fig Analysis of coding potential of Ginir lincRNA. (A) Representative phase contrast micrographs of cell transformation assay performed in NIH/3T3 cells during oncogene screen from cDNA library of Clone M3 cells. NIH/3T3 cells transfected with empty vector served as control in all experiments. (10× magnification). (B) A 557-base cDNA sequence exhibited transformed phenotype during the oncogene screen. Coloured parts (green and blue) represent two putative ORFs (ORF 2 and 3) in the sequence, and the red underlined part represents the overlapping ORF (ORF1) predicted using ORF finder (www.ncbi.nlm.nih.gov/orffinder). (C) The ORF analysis of the 557-base transcript determined using ORF Finder (www.ncbi.nlm.nih.gov/orffinder). The three putative ORFs—ORF1, ORF2, and ORF3—are represented as blue blocks, and the sequence denoting each of the ORFs is underlined in S1B Fig. (D) Western blot analysis of EGFP fusion proteins generated by cloning three ORFs (ORF1, ORF2, and ORF3) of Ginir independently in EGFP-N1 vector. Sixty μg of cell lysates was loaded onto the gel, and the blot was probed with antibody to GFP. β-actin served as a loading control. (E) Histogram showing mean volume of tumours obtained by in vivo tumourigenicity assay in NOD/SCID mice (*n* = 3) with the indicated transfectants. The tumours were harvested, and volume was measured after a period of 45 days post injection. Tumourigenicity assays were replicated twice with two independent transfectants. Supporting data for F and G can be found in S7 Data. Assessment of coding potential of the identified 557-base transcript using CPAT (F) and CPC2 tools (G). Tables show potential scores and coding probabilities. CPAT, Coding Potential Assessment Tool; CPC2, Coding Potential Calculator; EGFP, enhanced GFP; Ginir, Genomic Instability Inducing RNA; GFP, green fluorescent protein; lincRNA, long intergenic noncoding RNA.
(TIF)

S2 Fig Ginir/Giniras pair is expressed differentially during growth and transformation. (A) Schematic representation of transcripts (mRNAs and noncoding RNAs) bearing sequence homology to Ginir acquired using BLASTN with Refseq-RNA database of NCBblast (ncbi.nlm.nih.gov). (B) Genomic location of Ginir and Giniras transcripts on the mouse X chromosome obtained using UCSC genome browser (http://genome.ucsc.edu). (C) Strand-specific PCR for determination of Ginir/Giniras transcripts in NIH/3T3cells using G1F-G1R primers. Actin served as loading control. (D) Schematic representation of RNA contigs obtained on RNA-seq analyses of NIH/3T3 cells mapped to Ginir locus using blast.ncbi.nlm.nih.gov. Ginir, Genomic Instability Inducing RNA; Giniras, antisense RNA to Ginir; PCR, polymerase chain reaction; RNA-seq, RNA sequencing; UCSC, University of California, Santa Cruz.
(TIF)

S3 Fig Ginir overexpression causes rapid cycling of cells and induces invasive phenotype. (A) Quantification of Ginir and Giniras expression levels in NIH/3T3-EV, NIH/3T3-Ginir, and NIH/3T3-Giniras cells using G5F-G5R primers by strand-specific qRT-PCR. Values are mean ± SEM, ***P ≤ 0.0001 by Student’s *t* test (*n* = 3). (B) Representative RPA in NIH/3T3-Ginir cells with PCR-generated sense or antisense probes specific to Ginir sequence. Yeast total RNA served as control for RNase A/T1 activity. (C) Quantification of Ki67 immunostaining of mentioned cell lines, shown as percentage of Ki67 positively stained cells as compared to total number of cells per field assayed over 10 random fields. Data represent mean ± SEM. ***P ≤ 0.0001 by one-way ANOVA test (*n* = 3). (D and E) Representative cell cycle profiles of PI-stained cells determined using flow cytometry (D) Quantitative representation of cells in various cell cycle phases (E). Values are means + SEM; *P < 0.05, two tailed, by Fisher’s exact test (*n* = 3). (F) Representative blots for expression of Cdk4, Cyclin D1, Cdk2, Cyclin E, and pRb expression in the mentioned transfectants’ cell lines. Actin served as loading control. (G) Representative images of colonies visualised by soft agar assay for assessing clonogenicity of NIH/3T3-Ginir and NIH/3T3-Giniras cell lines. NIH/3T3-EV cell line served as control. (H) Representative images of Matrigel invasion assay performed with the indicated cell lines. Infiltrated cells were stained with crystal violet after 18 hours of incubation. (I) Analysis of cell migration in NIH/3T3-EV, NIH/3T3-Ginir, and NIH/3T3-Giniras cells measured by wound healing assay. The gap was measured after 20 hours using ImageJ software, version 1.41. (J) Quantitative analysis of relative wound recovery in each of the NIH/3T3-Ginir/Giniras cells as compared to control NIH/3T3-EV cells. Values represent mean ± SEM (*n* = 3). (K) Representative images of angiogenesis induction in CAM assay by the indicated cells. (L) Kaplan Meier survival curve showing survival period of mice injected with NIH/3T3-Ginir/Giniras and NIH/3T3-EV cell lines. Only mice injected with NIH/3T3-Ginir cells formed xenografts and showed mortality. Log-rank *P* value = 0.0351, chi-squared = 6.7 (*n* = 6) (M). Representative images showing metastatic foci in lungs of mice subcutaneously injected with Ginir transfectant cell lines (A and B). Lungs were harvested after a period of 11 weeks post injection. (N) HE staining of lungs of mice injected subcutaneously with NIH/3T3-Ginir cells. Supporting data for A, C, E, J, and L can be found in S8 Data. CAM, chicken chorioallantoic membrane; Cdk2, cyclin-dependent kinase 2; Cdk4, cyclin-dependent kinase 4; Ginir, Genomic Instability Inducing RNA; Giniras, antisense RNA to Ginir; HE, haematoxylin–eosin; PCR, polymerase chain reaction; PI, propidium iodide; pRb, phosphorylated retinoblastoma protein; qRT-PCR, quantitative reverse transcription PCR; RPA, ribonuclease protection assay.
(TIF)

S4 Fig RNA-seq analyses of Ginir-expressing cells. (A) Graph showing number of high-quality reads and aligned reads out of total number of raw reads generated from RNA-seq data of NIH-EV, NIH-GinirA, NIH-Giniras, and Clone M3 cells. Supporting data can be found in S9 Data. (B) Table showing percentage of aligned reads for NIH-EV, NIH-Ginir-A, NIH-Giniras, and Clone M3 to the mouse transcriptome. (C) Heatmap representing differentially expressed genes through whole-transcriptome sequencing of RNA derived from cells—NIH-EV versus NIH-GinirA, NIH-EV versus NIH-Giniras, NIH-Ginir versus NIH-Giniras, NIH-EV versus Clone M3, NIH-GinirA versus Clone M3, and NIH-Giniras versus Clone M3. (D) Heatmap representing differentially expressed genes through whole-transcriptome sequencing in NIH-GinirA as compared to NIH-EV. (E) Total number of up- and down-regulated genes in NIH-Ginir cells in comparison to NIH-EV control cells using *P* value of less than 0.05 using Cuffdiff analysis. (F) Enrichment in GO terms for the genes up-regulated in NIH-Ginir compared to NIH-EV for ‘Biological Process’. (G) Heatmap representing the relative expression of these genes (F) showing enrichment of these GO terms. (H) Enrichment in GO terms for the genes up-regulated in NIH-Ginir compared to NIH-EV for ‘Cellular Component’. (I) Heatmap representing the relative expression of these genes (H) showing enrichment of these GO terms. Supporting data for A can be found in S9 Data. Ginir, Genomic Instability Inducing RNA; Giniras, antisense RNA to Ginir; GO, Gene Ontology; RNA-seq, RNA sequencing.
(TIF)

S5 Fig Ginir knock-down restores normal fibroblast phenotype. (A) Representative phase contrast microscopy images of NIH/3T3-Ginir (Clone B) cells stably transfected with shGinir (1 and 2) and shControl (scrambled shRNA). Magnification—10×. (B) Immunofluorescence staining for Ki67 antigen expression in NIH/3T3-Ginir cells knocked down with Ginir shRNA1 and 2 and control shRNA. Nuclei were stained with DAPI. Scale bars—20 μm. (C) Quantitative analyses of cell cycle profiles of NIH-Ginir stable knock-down cells (NIHGinir-shGinir 1 and 2) and control cells (NIHGinir-shControl) by PI staining using flow cytometry. The S+G2/M population is plotted for each cell type. Values: means ± SEM; *P < 0.05, ***P < 0.0001; two tailed; by two-way ANOVA test (*n* = 3). Supporting data can be found in S10 Data. (D) Representative tumour pictures demonstrating the effect of Ginir-shRNA on tumour induction potential of NIH/3T3-Ginir (Clone B) cells assessed using NOD/SCID mice xenograft assay (*n* = 3). Tumour volumes were measured at a regular interval of 5 days, and the tumours were dissected after 30 days post injection. Both control and knock-down cells were subcutaneously injected on both sides (left and right) of the same mouse for better comparison. L1 and L2 indicate the left site of injection, whereas R1 and R2 indicate the right site of injection. Supporting data for C can be found in S10 Data. Ginir, Genomic Instability Inducing RNA; PI, propidium iodide; shRNA, short hairpin RNA.
(TIF)

S6 Fig Ginir knock-down affects proliferation and tumourigenicity of cells endogenously expressing Ginir. (A) Representative immunofluorescence images of Ki67 expression in cells stably transfected with Ginir shRNAs (1 and 2) and control shRNA. Nuclei were stained with DAPI. Scale bars—10 μm. (B) Confocal microscopy images for Ki67 staining in B16F10-shControl and B16F10-shGinir (1 and 2) cells. Nuclei were stained with DAPI. Scale bars—50 μm. (C) Representative tumour pictures demonstrating effect of Ginir-shRNA on tumour induction potential of B16F10 cells assessed using NOD/SCID mice xenograft assay (*n* = 3). Tumour volumes were measured at regular intervals, and the tumours were dissected after 10 days post injection. (D) Cell pellet pictures of equal number of B16F10 control and Ginir knock-down cells (shRNA 1 and 2). Ginir, Genomic Instability Inducing RNA; shRNA, short hairpin RNA.
(TIF)

S7 Fig Ginir is distributed in the cytoplasm of NIH-Giniras cells. (A and B) Fixed cells treated with RNase served as negative control for fluorescence in RNA-FISH of the respective cells (shown in Fig 6E–6H) using Ginir-specific LNA probes probe-1 (A) and probe-2 (B). Nuclei were stained with DAPI. Scale bars—10 μm (C). RNA-FISH confocal microscopy using LNA probes (Exiqon) for Giniras (TexRed labelled, Red) in NIH/3T3-Giniras cells. Fixed cells treated with RNase served as negative control for fluorescence. Nuclei were stained with DAPI. Scale bars—10 μm. Ginir, Genomic Instability Inducing RNA; Giniras, antisense RNA to Ginir; LNA, locked nucleic acid; RNA-FISH, RNA fluorescence in situ hybridisation.
(TIF)

S8 Fig Ginir cells exhibit preponderance of multinucleated cells. (A and B) Confocal images of different cell lines stained with DAPI. Scale bars—10 μm (A), 20 μm (A), and single DAPI-stained NIH/3T3-Ginir cell (B). (C) RNA-FISH of multinucleated NIH-Ginir cells using Ginir-specific LNA probe (green). Blue colour indicates nuclear staining with DAPI. Scale bars—10 μm. Ginir, Genomic Instability Inducing RNA; LNA, locked nucleic acid; RNA-FISH, RNA fluorescence in situ hybridisation.
(TIF)

S9 Fig Ginir physically interacts with Cep112 in mouse cells. (A) RNA pull-down assay with biotinylated Ginir probe (Hot and Cold probe) in NIH/3T3 cells followed by Coomassie staining for visualisation. (B) CatRAPID-based prediction of interaction between Ginir and Cep112. (C) Table listing the predicted isoforms of Cep112 obtained using Ensembl Genome browser (http://www.ensembl.org/Mus_musculus). The isoforms mainly expressed in NIH/3T3 cells are highlighted using coloured outlines. (D) Western blot of Ginir-interacting proteins obtained by RNA pull-down with biotinylated Ginir and Giniras probes in NIH/3T3 cells. Elutes were loaded on to 7% SDS-PAGE. Blot was probed with Cep112 antibody (24928-1-AP, Proteintech). Both hot (biotin probe) and cold (one-tenth of biotin CTP to that used in hot probe) probes for Ginir were used in pull-down assay. Unbiotinylated probe served as control. Blot was stripped and probed for tubulin as control for nonspecific binding. (E) Table representing the probability scores for interaction of different isoforms of Cep112 with Ginir transcript using catRAPID and RIPseq tools. (F) Western blot analysis of Cep112 expression in NIH/3T3-EV, NIH-Ginir, and NIH-Giniras cells. Blot was probed with Cep112 antibody (24928-1-AP, Proteintech). Tubulin served as loading control. (G) RT-PCR analyses for expression of Cep112 transcript. Gapdh served as internal control. (H) Confocal microscopy images of mentioned interphase cells stained with Cep112 antibody. Scale bars—10 μm. Cep112, centrosomal protein 112; Gapdh, glyceride 3-phosphate dehydrogenase; Ginir, Genomic Instability Inducing RNA; Giniras, antisense RNA to Ginir; RT-PCR, reverse transcription polymerase chain reaction.
(TIF)

S10 Fig Ginir affects the interaction between Cep112 and DNA damage repair protein Brca1. (A) Western blot of Ginir-associated proteins obtained by RNA pull-down with biotinylated Ginir and Giniras probes in NIH/3T3 cells. Elutes were loaded on to 7% SDS-PAGE. Blot was probed with Brca1 antibody. Biotinylated antisense (Giniras) probe served as negative control. Unrelated biotin probes like XEF RNA and Hotair were also used as nonspecific controls. (B) Coimmunoprecipitation of Flag-Cep112 and Brca1 was carried out with lysates prepared from NIH-FlagCep112 cells. Whole-cell proteins were immunoprecipitated with Brca1 antibody (sc-646, Santa Cruz) and IB using Flag antibody. IgG immunoprecipitate served as negative control. (C and D) Coimmunoprecipitation of Brca1 and Cep112 in NIH/3T3-GinirA (C) and NIH/3T3-GinirB (D) cells wherein immunoprecipitates were treated independently with RNase and RNasin. Both Rnase- and RNasin-treated lysates were immunoprecipitated with Brca1 antibody (sc-646, Santa Cruz) and blotted with Cep112 antibody (sc-246163, Santa Cruz). Immunoprecipitation with anti-IgG served as control. The data for NIH/3T3-Ginir(A) transfectant are shown at three different exposures (short time—exposure I, II) and exposure at longer time, whereas data for NIH/3T3-Ginir(B) transfectant are shown at only one exposure. Ub obtained post immunoprecipitation was also loaded along with eluted fractions. (E) Confocal microscopy images of cells stained with Brca1 antibody. Scale bars, 20 μm. Arrowheads point towards centrosomal localisation of Brca1. Nuclei were stained with DAPI. (F) CatRAPID-based prediction of interaction between Brca1 and Ginir represented as a map. Brca1, breast cancer type 1 susceptibility protein; Cep112, centrosomal protein 112; Ginir, Genomic Instability Inducing RNA; Giniras, antisense RNA to Ginir; IB, immunoblotted; Hotair, HOX transcript antisense RNA; IgG, immunoglobulin G; RNasin, RNase inhibitor; Ub, unbound fraction; XEF, Xenopus elongation factor.
(TIF)

S11 Fig Schematic diagram of mechanism of action of Ginir/Ginras transcript pair. Excess levels of Ginir RNA cause imbalance in cellular homeostasis and propel cells towards malignant transformation. Ginir, Genomic Instability Inducing RNA; Giniras, antisense RNA to Ginir.
(TIF)

S1 Text MALDI-TOF and CatRAPID analysis for Ginir RNA. Interacting protein partners of Ginir RNA identified by RNA affinity pull-down assay. Ginir, Genomic Instability Inducing RNA; MALDI-TOF, matrix-assisted laser desorption ionisation time-of-flight mass spectrometry.
(DOCX)

S1 Table Raw reads in the RNA-seq data of NIH/3T3 cell line. The raw reads (approximately 100 bases) obtained in the RNA-seq data of NIH/3T3 cells align with the region between 61971212–61993884 of X chromosome of mouse corresponding to the Ginir sequence. Ginir, Genomic Instability Inducing RNA; RNA-seq, RNA sequencing.
(XLSX)

S2 Table Interacting protein partners of Ginir. Proteins identified by mass spectrometry from biotinylated-Ginir RNA pull-down assays. Ginir, Genomic Instability Inducing RNA.
(XLSX)

S3 Table Primer sequences. Sequences of primers used in RACE, RT-PCR, and qRT-PCR assays. qRT-PCR, quantitative RT-PCR; RACE, rapid amplification of cDNA ends; RT-PCR, reverse transcription polymerase chain reaction.
(XLSX)

S1 Data Excel file containing the underlying numerical data for Fig 3B, 3C, 3E and 3G. (XLSX)

S2 Data Excel file containing the underlying numerical data for Fig 4C, 4D, 4F and 4H. (XLS)

S3 Data Excel file containing the underlying numerical data for Fig 5B, 5C, 5D, 5G, 5H, 5J and 5L. (XLSX)

S4 Data Excel file containing the underlying numerical data for Fig 7C and 7F. (XLS)

S5 Data Excel file containing the underlying numerical data for Fig 8B and 8J. (XLS)

S6 Data Excel file containing the underlying numerical data for Fig 11E, 11G and 11J. (XLS)

S7 Data Excel file containing the underlying numerical data for S1E Fig. (XLSX)

S8 Data Excel file containing the underlying numerical data for S3A, S3C, S3E, S3J and S3L Fig. (XLSX)

S9 Data Excel file containing the underlying numerical data for S4A Fig. (XLSX)

S10 Data Excel file containing the underlying numerical data for S5C Fig. Data are given in indicated separate sheets.
(XLSX)

## Abbreviations

53BP1: p53 binding protein 1
aa: amino acids
ADC: automatic directional cloning
ANRASSF1: antisense intronic noncoding RASSF1
ANRIL: antisense noncoding RNA in the INK4 locus
AP1: adapter primer 1
AP2: adapter primer 2
ATM: ataxia–telangiectasia-mutated
ATR: ataxia–telangiectasia and Rad3-related kinase
Brca1: breast cancer type 1 susceptibility protein
CAM: chicken chorioallantoic membrane
CCAL: colorectal cancer–associated lncRNA
Cep112: centrosomal protein 112
ceRNA: competing endogenous RNA
cheRNA: chromatin-enriched RNA
CMV: cytomegalovirus
CNS: central nervous system
CPAT: Coding Potential Assessment Tool
CPC2: Coding Potential Calculator
CREB: cAMP response element binding protein
DANCR: differentiation antagonizing non-protein-coding RNA
DDR: DNA damage response
DDSR1: DNA damage–sensitive RNA1
dpc: days post coitum
dsDNA: double-stranded DNA
ERV: endogenous retrovirus
EST: expressed sequence tag
FACS: fluorescence-activated cell sorting
FAM: fluorescein amidite
Fbx13: F-box only protein 13
Fendrr: FOXF1 adjacent noncoding developmental regulatory RNA
FISH: fluorescence in situ hybridisation
GFP: green fluorescent protein
Ginir: Genomic Instability Inducing RNA
Giniras: antisense RNA to Ginir
GO: Gene Ontology
HE: haematoxylin–eosin
HIDALGO: hemin-induced cheRNA downstream of foetal haemoglobin
HIF-1a: hypoxia-inducible factor 1-alpha
HIFCAR: HIF-1a coactivating RNA
Hotair: HOX transcript antisense RNA
HULC: hepatocellular carcinoma up-regulated long noncoding RNA
IGF2: insulin-like growth factor 2
IGF2BP1: insulin-like growth factor 2 mRNA-binding protein 1
Kif20b: kinesin family member 20b
KRT7-AS: keratin type II cytoskeleton 7 antisense RNA 1
LDOC1: Leucine Zipper down-regulated in cancer-1
lincRNA: long intergenic noncoding RNA
LINE/L1: long interspersed nuclear element
LNA: locked nucleic acid
lncRNA: long noncoding RNA
LR: local repeat
LTR: long terminal repeat
LUADT1: lung adenocarcinoma transcript 1
MAGE: the melanoma antigen gene
MALAT1: metastasis associated lung adenocarcinoma transcript 1
MALDI-TOF: matrix-assisted laser desorption ionisation time-of-flight mass spectrometry
MHM: male hypermethylated
Mre11: meiotic recombination 11
MTT: 3-(4, 5-dimethylthiazol-2-yl)-2, 5-diphenyltetrazolium bromide
MyoD: myogenic differentiation
NAT: natural antisense transcript
NCBI: National Center for Biotechnology Information
ndsRNA: natural double-stranded RNA
NEAT1: nuclear enriched abundant transcript 1
Nlp: ninein-like protein
NORAD: noncoding RNA activated by DNA damage
OLA1: Obg-like ATPase 1
PANDA: P21-associated noncoding RNA DNA damage-activated
PCAT1: prostate cancer–associated transcript 1
PCR: polymerase chain reaction
PI: propidium iodide
pRb: phosphorylated retinoblastoma protein
PRC1/2: polycomb repressive complex 1/2
PTGS: posttranscriptional gene silencing
PVT1: plasmacytoma variant translocation 1
RAN: ras-related nuclear protein
RBP: RNA-binding protein
RCC1: regulator of chromosome condensation 1
RIP: RNA-immunoprecipitation
RNA-seq: RNA sequencing
RNasin: RNase inhibitor
RoR: regulator of reprogramming
RPA: RNase protection assay
RT-PCR: reverse transcription PCR
shRNA: short hairpin RNA
SINEB2: short interspersed nuclear element B2
Sirt1-AS: Sirtuin 1 antisense RNA
snRNA: small nuclear RNA
TE: transposable element
TR: tandem repeat
TUG1: taurine up-regulated gene 1
UCA1: urothelial cancer associated 1
Uchl1: ubiquitin carboxy-terminal hydrolase L1
XEF: Xenopus elongation factor
Xist: X-inactive specific transcript
