# Supplementary figures and images for: Noncoding RNA Ginir functions as an oncogene by associating with centrosomal proteins
Source: PLoS Biol. 2018 Oct 8;16(10):e2004204. doi: 10.1371/journal.pbio.2004204 (PMC6193740; doi:10.1371/journal.pbio.2004204)

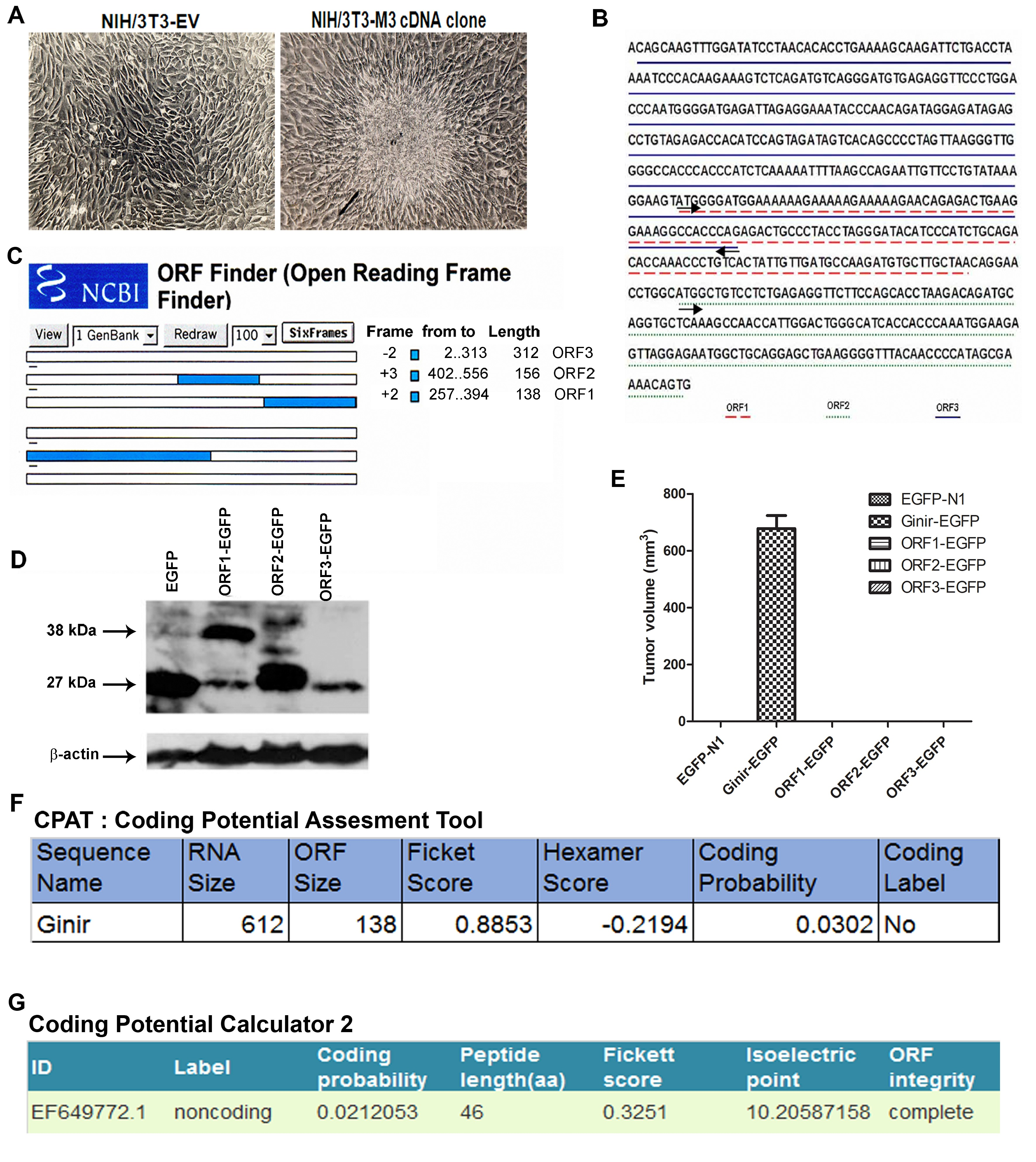

Supplement: S1 Fig — (A) Representative phase contrast micrographs of cell transformation assay performed in NIH/3T3 cells during oncogene screen from cDNA library of Clone M3 cells. NIH/3T3 cells transfected with empty vector served as control in all experiments. (10× magnification). (B) A 557-base cDNA sequence exhibited transformed phenotype during the oncogene screen. Coloured parts (green and blue) represent two putative ORFs (ORF 2 and 3) in the sequence, and the red underlined part represents the overlapping ORF (ORF1) predicted using ORF finder (www.ncbi.nlm.nih.gov/orffinder). (C) The ORF analysis of the 557-base transcript determined using ORF Finder (www.ncbi.nlm.nih.gov/orffinder). The three putative ORFs—ORF1, ORF2, and ORF3—are represented as blue blocks, and the sequence denoting each of the ORFs is underlined in S1B Fig. (D) Western blot analysis of EGFP fusion proteins generated by cloning three ORFs (ORF1, ORF2, and ORF3) of Ginir independently in EGFP-N1 vector. Sixty μg of cell lysates was loaded onto the gel, and the blot was probed with antibody to GFP. β-actin served as a loading control. (E) Histogram showing mean volume of tumours obtained by in vivo tumourigenicity assay in NOD/SCID mice (n = 3) with the indicated transfectants. The tumours were harvested, and volume was measured after a period of 45 days post injection. Tumourigenicity assays were replicated twice with two independent transfectants. Supporting data for F and G can be found in S7 Data. Assessment of coding potential of the identified 557-base transcript using CPAT (F) and CPC2 tools (G). Tables show potential scores and coding probabilities. CPAT, Coding Potential Assessment Tool; CPC2, Coding Potential Calculator; EGFP, enhanced GFP; Ginir, Genomic Instability Inducing RNA; GFP, green fluorescent protein; lincRNA, long intergenic noncoding RNA. (TIF) [file pbio.2004204.s001.tif]

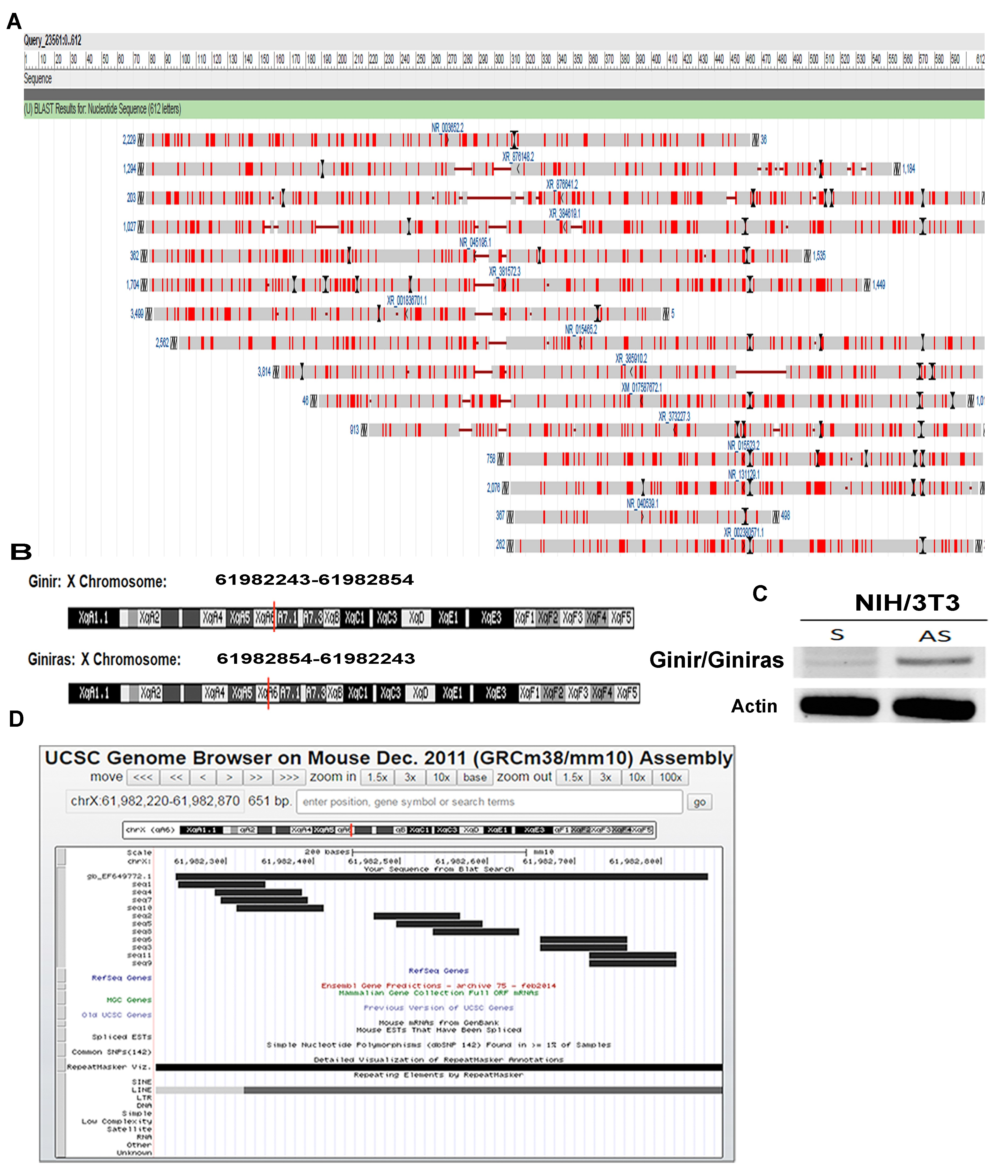

Supplement: S2 Fig — (A) Schematic representation of transcripts (mRNAs and noncoding RNAs) bearing sequence homology to Ginir acquired using BLASTN with Refseq-RNA database of NCBblast (ncbi.nlm.nih.gov). (B) Genomic location of Ginir and Giniras transcripts on the mouse X chromosome obtained using UCSC genome browser (http://genome.ucsc.edu). (C) Strand-specific PCR for determination of Ginir/Giniras transcripts in NIH/3T3cells using G1F-G1R primers. Actin served as loading control. (D) Schematic representation of RNA contigs obtained on RNA-seq analyses of NIH/3T3 cells mapped to Ginir locus using blast.ncbi.nlm.nih.gov. Ginir, Genomic Instability Inducing RNA; Giniras, antisense RNA to Ginir; PCR, polymerase chain reaction; RNA-seq, RNA sequencing; UCSC, University of California, Santa Cruz. (TIF) [file pbio.2004204.s002.tif]

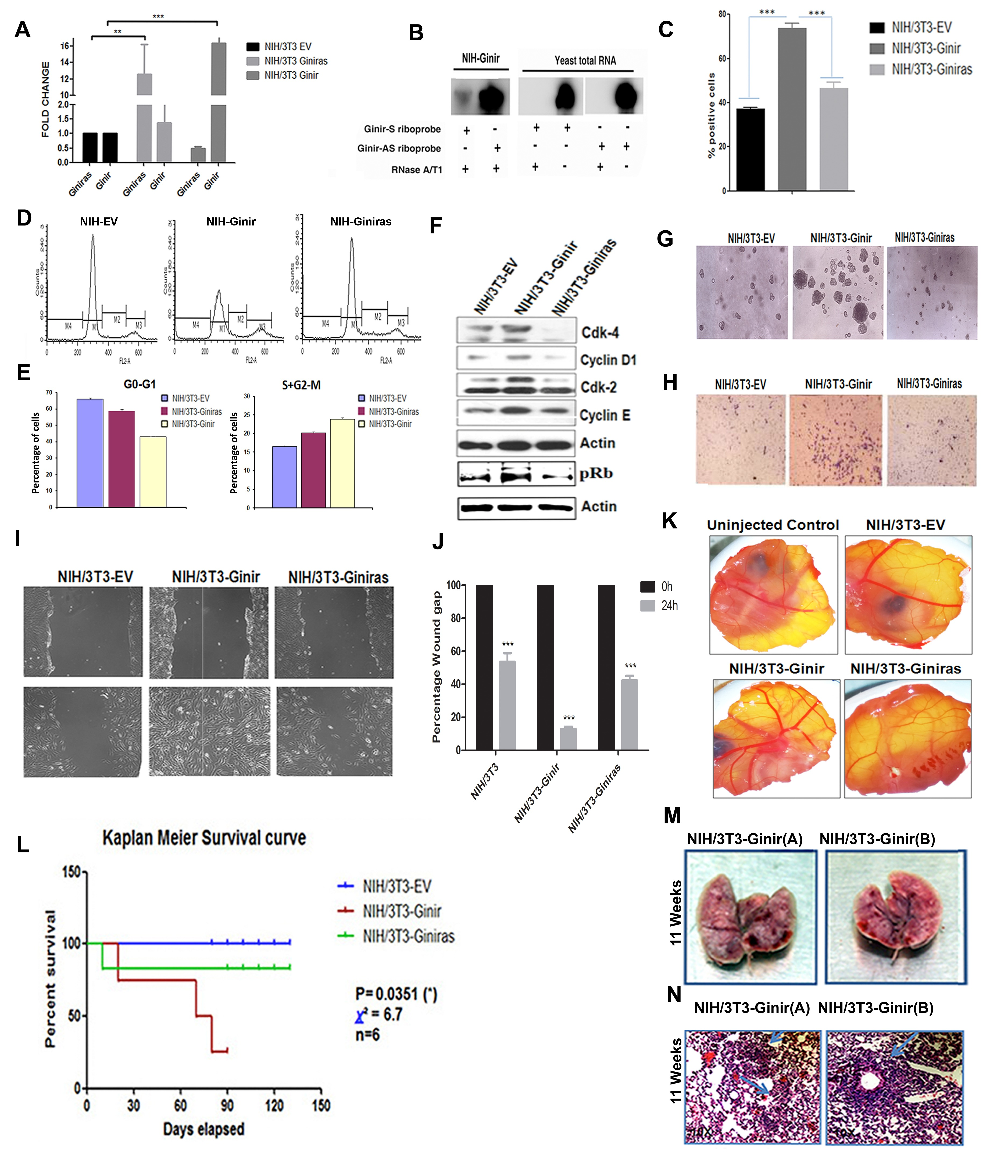

Supplement: S3 Fig — (A) Quantification of Ginir and Giniras expression levels in NIH/3T3-EV, NIH/3T3-Ginir, and NIH/3T3-Giniras cells using G5F-G5R primers by strand-specific qRT-PCR. Values are mean ± SEM, ***P ≤ 0.0001 by Student’s t test (n = 3). (B) Representative RPA in NIH/3T3-Ginir cells with PCR-generated sense or antisense probes specific to Ginir sequence. Yeast total RNA served as control for RNase A/T1 activity. (C) Quantification of Ki67 immunostaining of mentioned cell lines, shown as percentage of Ki67 positively stained cells as compared to total number of cells per field assayed over 10 random fields. Data represent mean ± SEM. ***P ≤ 0.0001 by one-way ANOVA test (n = 3). (D and E) Representative cell cycle profiles of PI-stained cells determined using flow cytometry (D) Quantitative representation of cells in various cell cycle phases (E). Values are means + SEM; *P < 0.05, two tailed, by Fisher’s exact test (n = 3). (F) Representative blots for expression of Cdk4, Cyclin D1, Cdk2, Cyclin E, and pRb expression in the mentioned transfectants’ cell lines. Actin served as loading control. (G) Representative images of colonies visualised by soft agar assay for assessing clonogenicity of NIH/3T3-Ginir and NIH/3T3-Giniras cell lines. NIH/3T3-EV cell line served as control. (H) Representative images of Matrigel invasion assay performed with the indicated cell lines. Infiltrated cells were stained with crystal violet after 18 hours of incubation. (I) Analysis of cell migration in NIH/3T3-EV, NIH/3T3-Ginir, and NIH/3T3-Giniras cells measured by wound healing assay. The gap was measured after 20 hours using ImageJ software, version 1.41. (J) Quantitative analysis of relative wound recovery in each of the NIH/3T3-Ginir/Giniras cells as compared to control NIH/3T3-EV cells. Values represent mean ± SEM (n = 3). (K) Representative images of angiogenesis induction in CAM assay by the indicated cells. (L) Kaplan Meier survival curve showing survival period of mice injected with NIH/3 [file pbio.2004204.s003.tif]

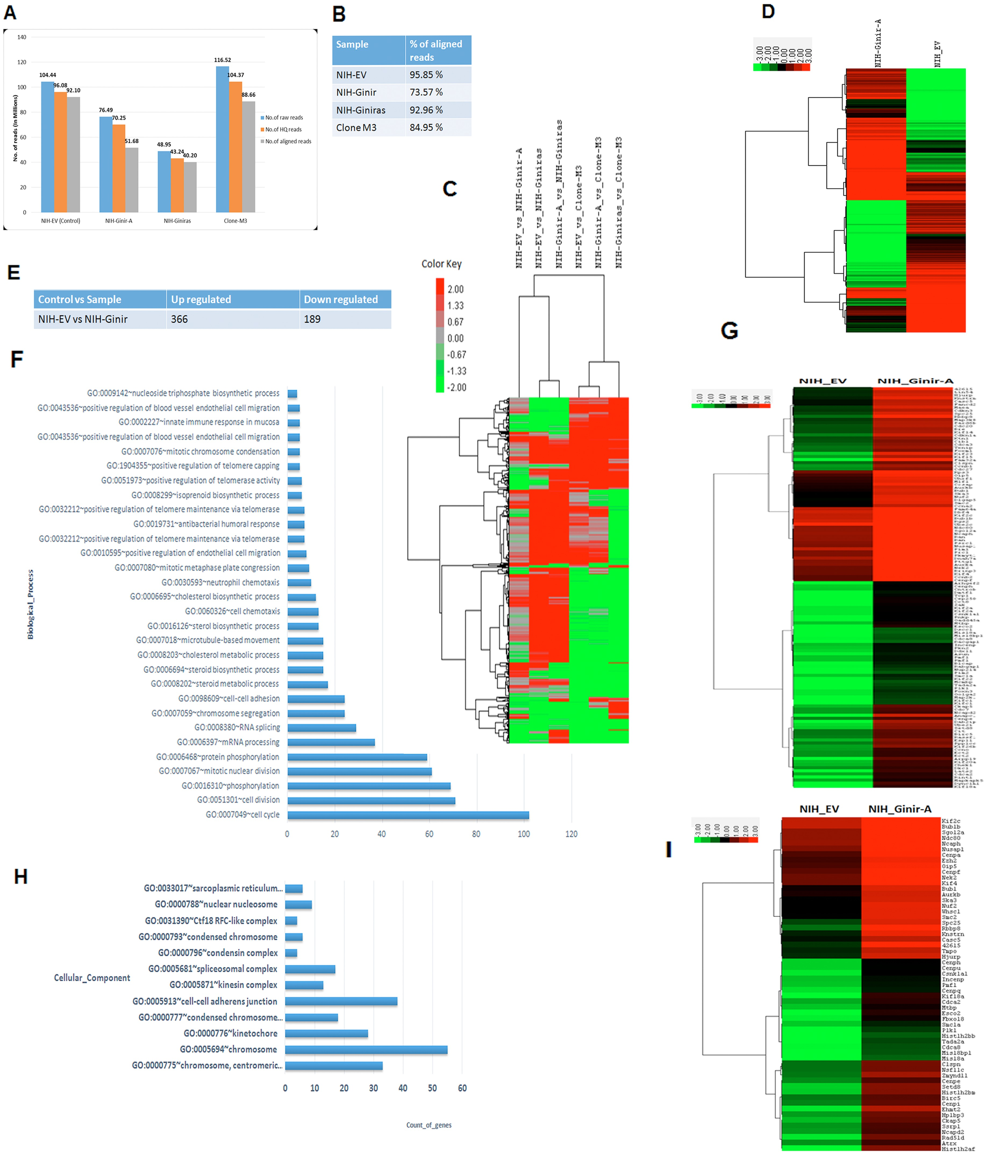

Supplement: S4 Fig — (A) Graph showing number of high-quality reads and aligned reads out of total number of raw reads generated from RNA-seq data of NIH-EV, NIH-GinirA, NIH-Giniras, and Clone M3 cells. Supporting data can be found in S9 Data. (B) Table showing percentage of aligned reads for NIH-EV, NIH-Ginir-A, NIH-Giniras, and Clone M3 to the mouse transcriptome. (C) Heatmap representing differentially expressed genes through whole-transcriptome sequencing of RNA derived from cells—NIH-EV versus NIH-GinirA, NIH-EV versus NIH-Giniras, NIH-Ginir versus NIH-Giniras, NIH-EV versus Clone M3, NIH-GinirA versus Clone M3, and NIH-Giniras versus Clone M3. (D) Heatmap representing differentially expressed genes through whole-transcriptome sequencing in NIH-GinirA as compared to NIH-EV. (E) Total number of up- and down-regulated genes in NIH-Ginir cells in comparison to NIH-EV control cells using P value of less than 0.05 using Cuffdiff analysis. (F) Enrichment in GO terms for the genes up-regulated in NIH-Ginir compared to NIH-EV for ‘Biological Process’. (G) Heatmap representing the relative expression of these genes (F) showing enrichment of these GO terms. (H) Enrichment in GO terms for the genes up-regulated in NIH-Ginir compared to NIH-EV for ‘Cellular Component’. (I) Heatmap representing the relative expression of these genes (H) showing enrichment of these GO terms. Supporting data for A can be found in S9 Data. Ginir, Genomic Instability Inducing RNA; Giniras, antisense RNA to Ginir; GO, Gene Ontology; RNA-seq, RNA sequencing. (TIF) [file pbio.2004204.s004.tif]

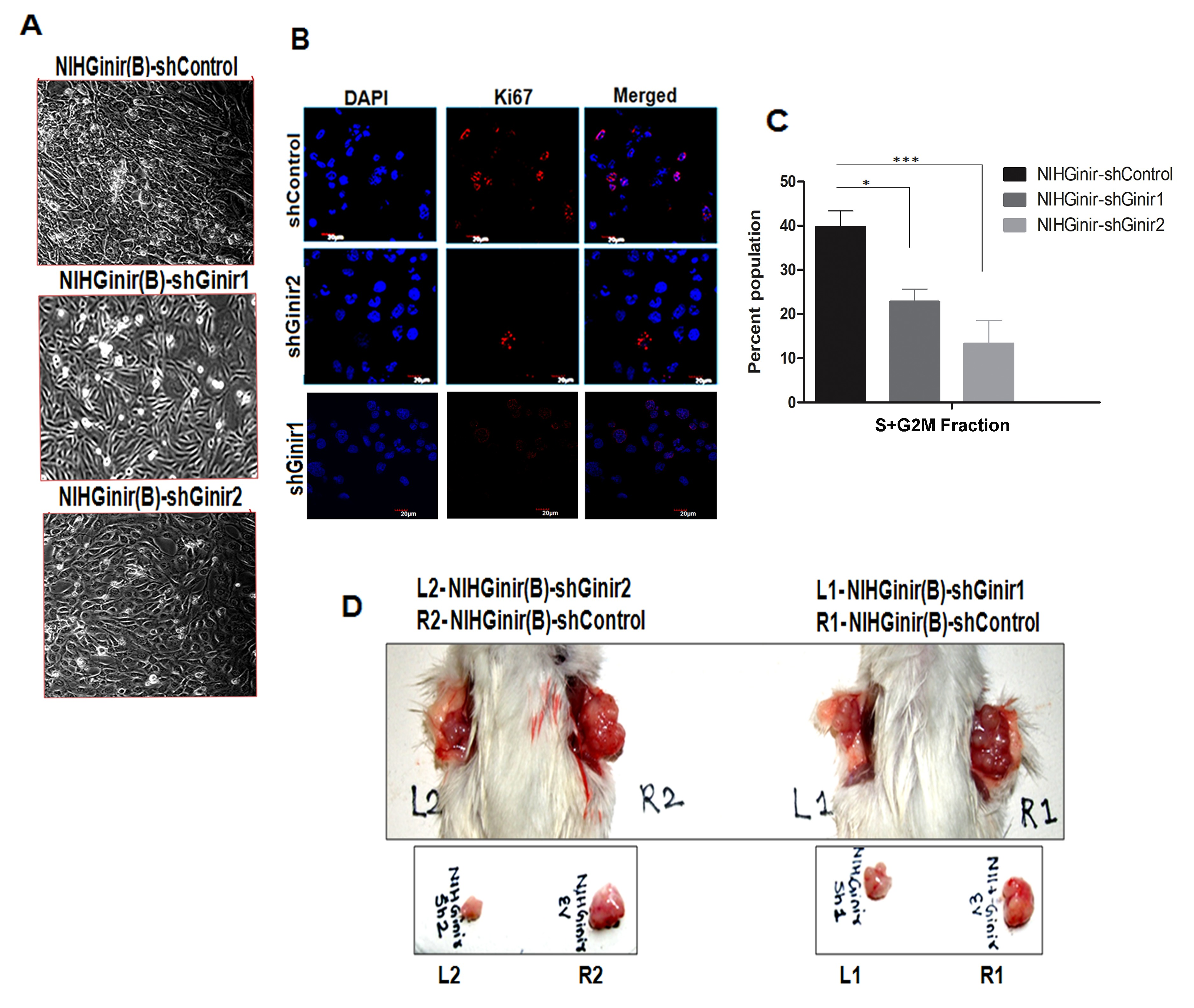

Supplement: S5 Fig — (A) Representative phase contrast microscopy images of NIH/3T3-Ginir (Clone B) cells stably transfected with shGinir (1 and 2) and shControl (scrambled shRNA). Magnification—10×. (B) Immunofluorescence staining for Ki67 antigen expression in NIH/3T3-Ginir cells knocked down with Ginir shRNA1 and 2 and control shRNA. Nuclei were stained with DAPI. Scale bars—20 μm. (C) Quantitative analyses of cell cycle profiles of NIH-Ginir stable knock-down cells (NIHGinir-shGinir 1 and 2) and control cells (NIHGinir-shControl) by PI staining using flow cytometry. The S+G2/M population is plotted for each cell type. Values: means ± SEM; *P < 0.05, ***P < 0.0001; two tailed; by two-way ANOVA test (n = 3). Supporting data can be found in S10 Data. (D) Representative tumour pictures demonstrating the effect of Ginir-shRNA on tumour induction potential of NIH/3T3-Ginir (Clone B) cells assessed using NOD/SCID mice xenograft assay (n = 3). Tumour volumes were measured at a regular interval of 5 days, and the tumours were dissected after 30 days post injection. Both control and knock-down cells were subcutaneously injected on both sides (left and right) of the same mouse for better comparison. L1 and L2 indicate the left site of injection, whereas R1 and R2 indicate the right site of injection. Supporting data for C can be found in S10 Data. Ginir, Genomic Instability Inducing RNA; PI, propidium iodide; shRNA, short hairpin RNA. (TIF) [file pbio.2004204.s005.tif]

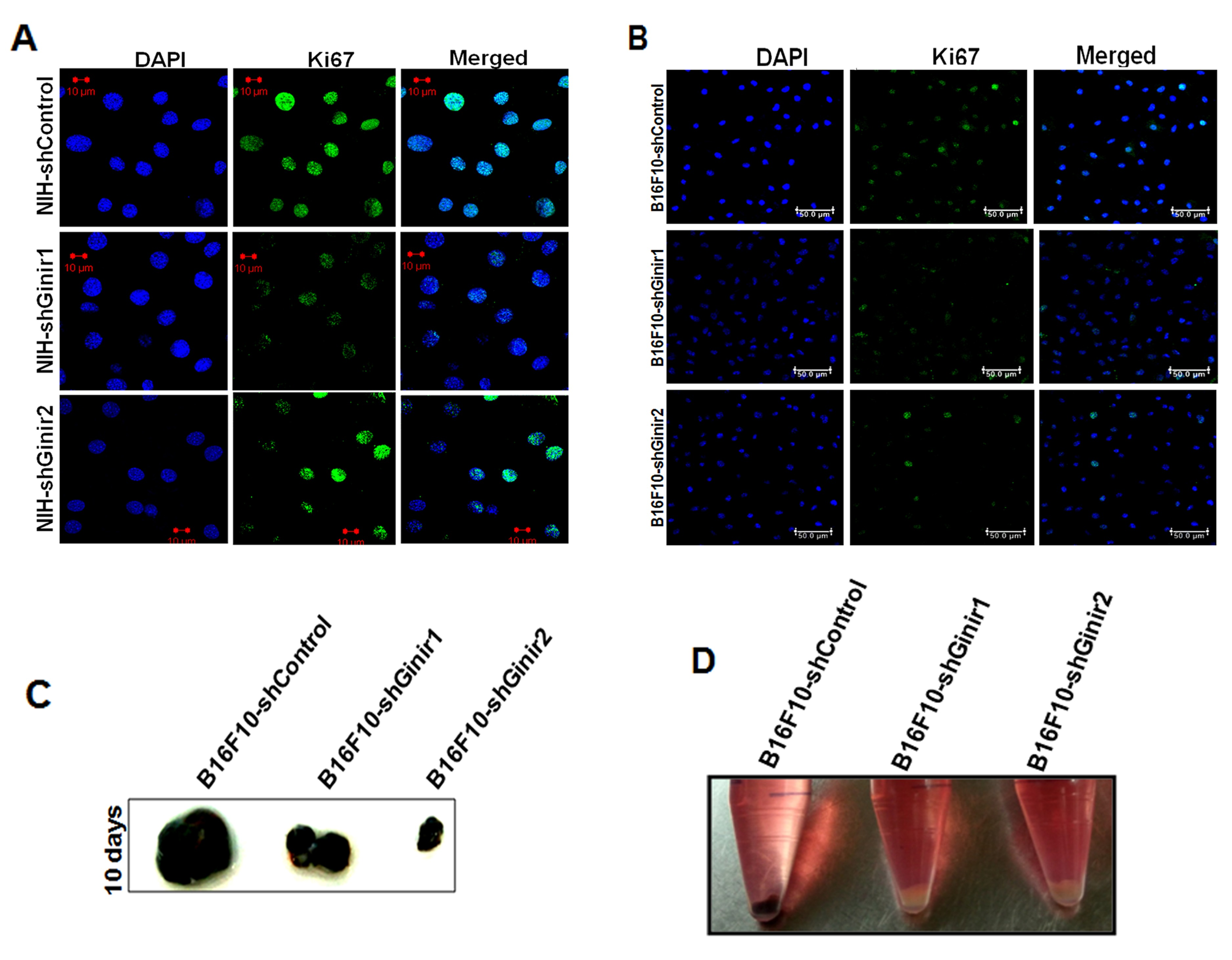

Supplement: S6 Fig — (A) Representative immunofluorescence images of Ki67 expression in cells stably transfected with Ginir shRNAs (1 and 2) and control shRNA. Nuclei were stained with DAPI. Scale bars—10 μm. (B) Confocal microscopy images for Ki67 staining in B16F10-shControl and B16F10-shGinir (1 and 2) cells. Nuclei were stained with DAPI. Scale bars—50 μm. (C) Representative tumour pictures demonstrating effect of Ginir-shRNA on tumour induction potential of B16F10 cells assessed using NOD/SCID mice xenograft assay (n = 3). Tumour volumes were measured at regular intervals, and the tumours were dissected after 10 days post injection. (D) Cell pellet pictures of equal number of B16F10 control and Ginir knock-down cells (shRNA 1 and 2). Ginir, Genomic Instability Inducing RNA; shRNA, short hairpin RNA. (TIF) [file pbio.2004204.s006.tif]

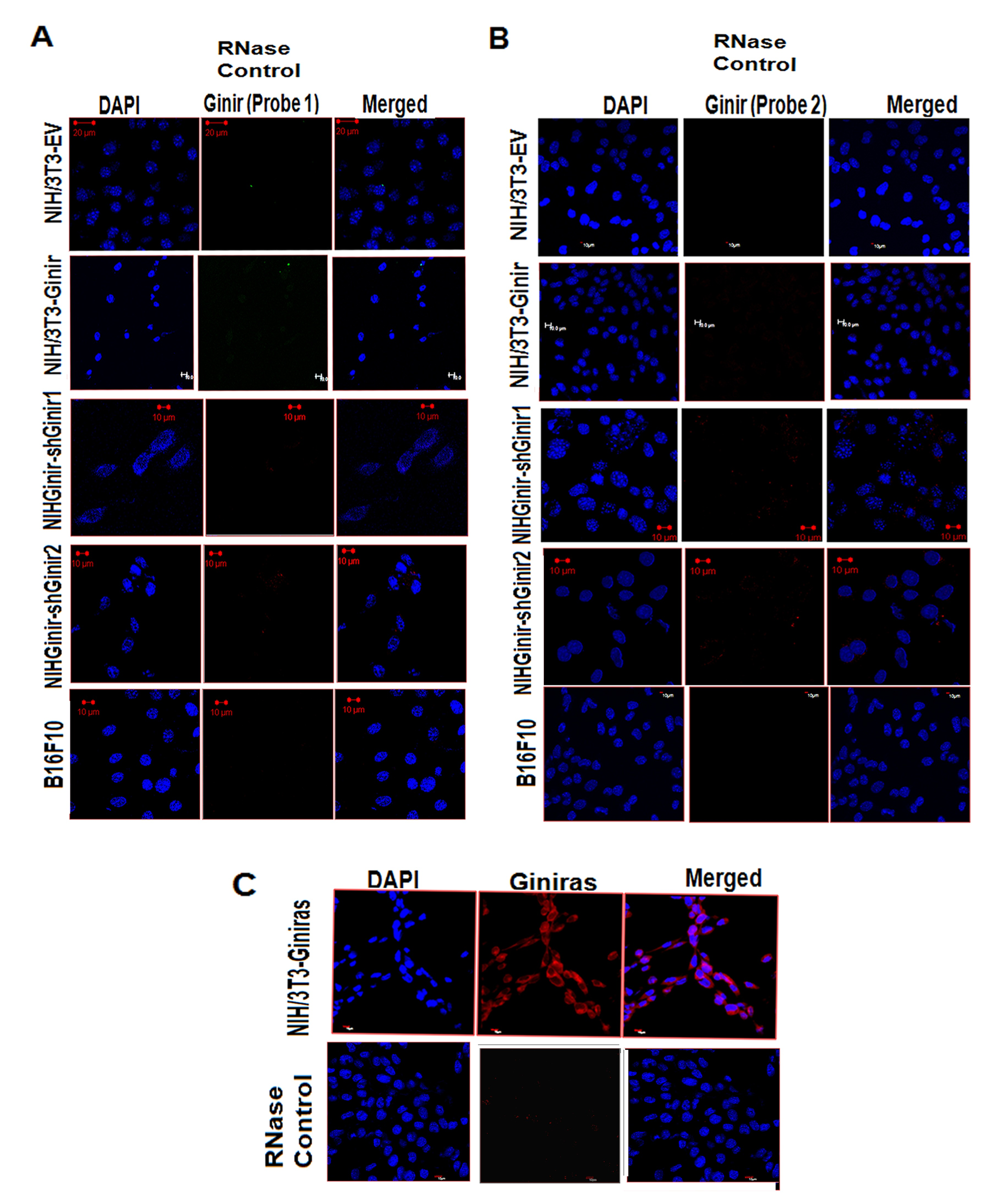

Supplement: S7 Fig — (A and B) Fixed cells treated with RNase served as negative control for fluorescence in RNA-FISH of the respective cells (shown in Fig 6E–6H) using Ginir-specific LNA probes probe-1 (A) and probe-2 (B). Nuclei were stained with DAPI. Scale bars—10 μm (C). RNA-FISH confocal microscopy using LNA probes (Exiqon) for Giniras (TexRed labelled, Red) in NIH/3T3-Giniras cells. Fixed cells treated with RNase served as negative control for fluorescence. Nuclei were stained with DAPI. Scale bars—10 μm. Ginir, Genomic Instability Inducing RNA; Giniras, antisense RNA to Ginir; LNA, locked nucleic acid; RNA-FISH, RNA fluorescence in situ hybridisation. (TIF) [file pbio.2004204.s007.tif]

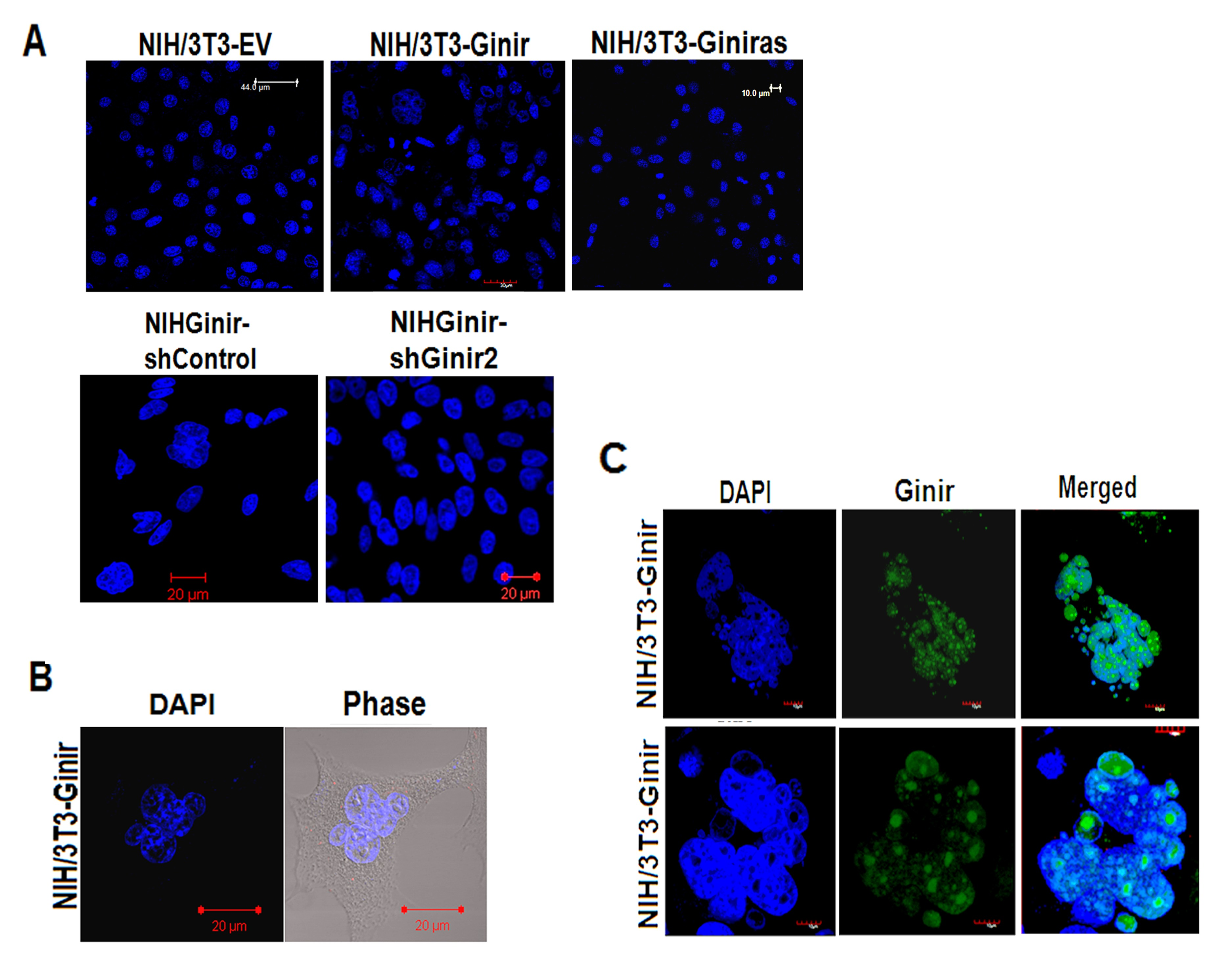

Supplement: S8 Fig — (A and B) Confocal images of different cell lines stained with DAPI. Scale bars—10 μm (A), 20 μm (A), and single DAPI-stained NIH/3T3-Ginir cell (B). (C) RNA-FISH of multinucleated NIH-Ginir cells using Ginir-specific LNA probe (green). Blue colour indicates nuclear staining with DAPI. Scale bars—10 μm. Ginir, Genomic Instability Inducing RNA; LNA, locked nucleic acid; RNA-FISH, RNA fluorescence in situ hybridisation. (TIF) [file pbio.2004204.s008.tif]

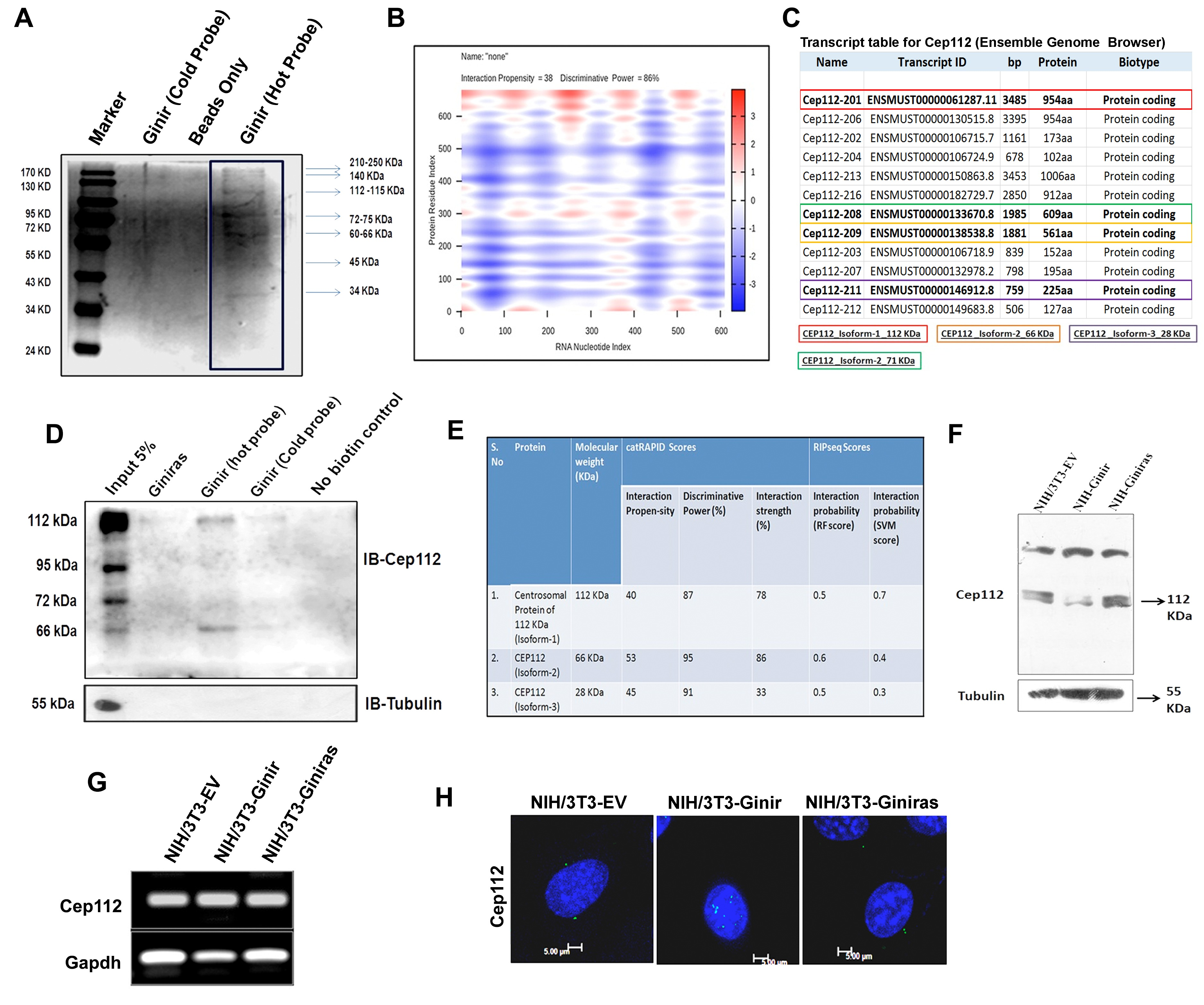

Supplement: S9 Fig — (A) RNA pull-down assay with biotinylated Ginir probe (Hot and Cold probe) in NIH/3T3 cells followed by Coomassie staining for visualisation. (B) CatRAPID-based prediction of interaction between Ginir and Cep112. (C) Table listing the predicted isoforms of Cep112 obtained using Ensembl Genome browser (http://www.ensembl.org/Mus_musculus). The isoforms mainly expressed in NIH/3T3 cells are highlighted using coloured outlines. (D) Western blot of Ginir-interacting proteins obtained by RNA pull-down with biotinylated Ginir and Giniras probes in NIH/3T3 cells. Elutes were loaded on to 7% SDS-PAGE. Blot was probed with Cep112 antibody (24928-1-AP, Proteintech). Both hot (biotin probe) and cold (one-tenth of biotin CTP to that used in hot probe) probes for Ginir were used in pull-down assay. Unbiotinylated probe served as control. Blot was stripped and probed for tubulin as control for nonspecific binding. (E) Table representing the probability scores for interaction of different isoforms of Cep112 with Ginir transcript using catRAPID and RIPseq tools. (F) Western blot analysis of Cep112 expression in NIH/3T3-EV, NIH-Ginir, and NIH-Giniras cells. Blot was probed with Cep112 antibody (24928-1-AP, Proteintech). Tubulin served as loading control. (G) RT-PCR analyses for expression of Cep112 transcript. Gapdh served as internal control. (H) Confocal microscopy images of mentioned interphase cells stained with Cep112 antibody. Scale bars—10 μm. Cep112, centrosomal protein 112; Gapdh, glyceride 3-phosphate dehydrogenase; Ginir, Genomic Instability Inducing RNA; Giniras, antisense RNA to Ginir; RT-PCR, reverse transcription polymerase chain reaction. (TIF) [file pbio.2004204.s009.tif]

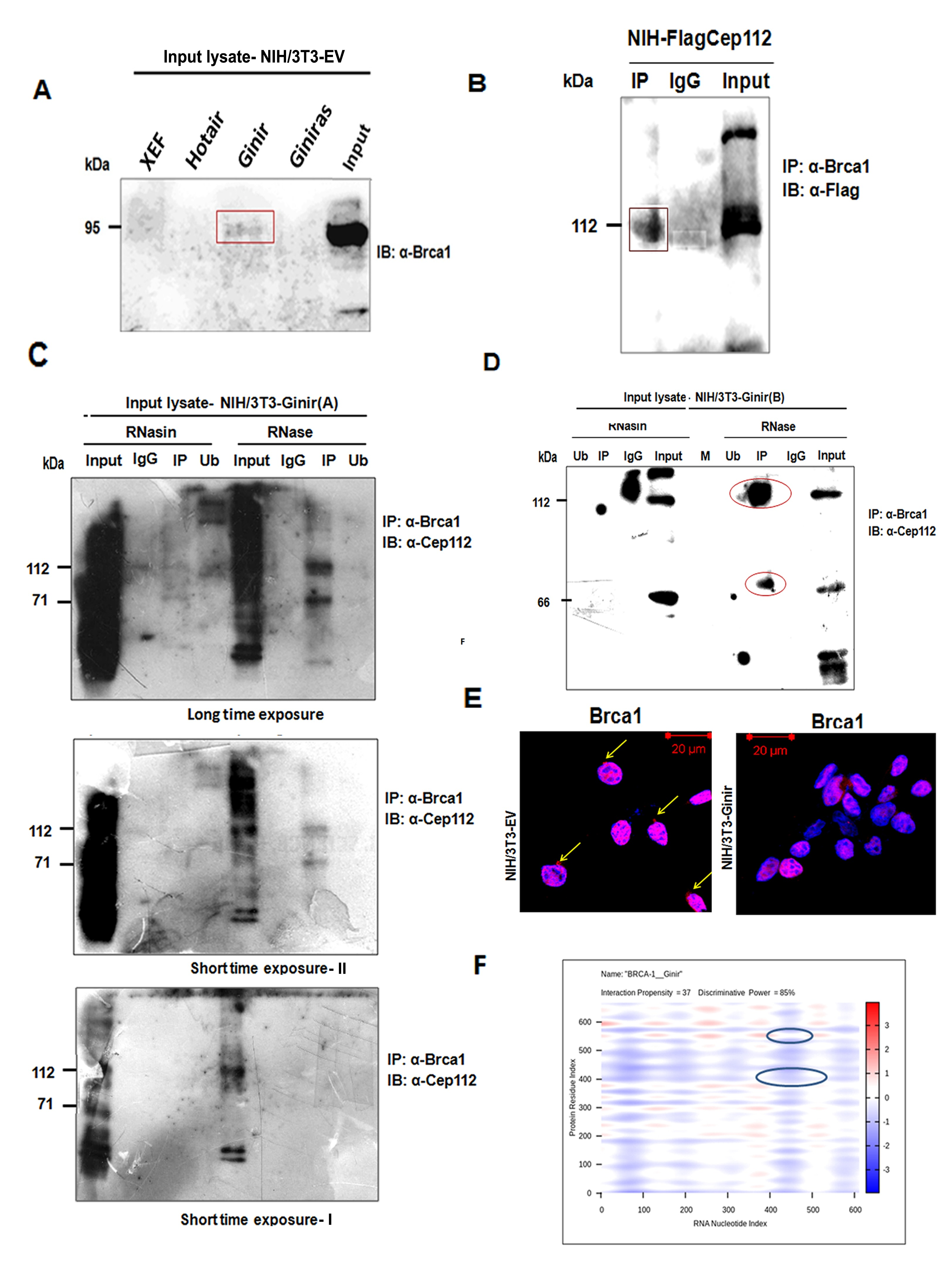

Supplement: S10 Fig — (A) Western blot of Ginir-associated proteins obtained by RNA pull-down with biotinylated Ginir and Giniras probes in NIH/3T3 cells. Elutes were loaded on to 7% SDS-PAGE. Blot was probed with Brca1 antibody. Biotinylated antisense (Giniras) probe served as negative control. Unrelated biotin probes like XEF RNA and Hotair were also used as nonspecific controls. (B) Coimmunoprecipitation of Flag-Cep112 and Brca1 was carried out with lysates prepared from NIH-FlagCep112 cells. Whole-cell proteins were immunoprecipitated with Brca1 antibody (sc-646, Santa Cruz) and IB using Flag antibody. IgG immunoprecipitate served as negative control. (C and D) Coimmunoprecipitation of Brca1 and Cep112 in NIH/3T3-GinirA (C) and NIH/3T3-GinirB (D) cells wherein immunoprecipitates were treated independently with RNase and RNasin. Both Rnase- and RNasin-treated lysates were immunoprecipitated with Brca1 antibody (sc-646, Santa Cruz) and blotted with Cep112 antibody (sc-246163, Santa Cruz). Immunoprecipitation with anti-IgG served as control. The data for NIH/3T3-Ginir(A) transfectant are shown at three different exposures (short time—exposure I, II) and exposure at longer time, whereas data for NIH/3T3-Ginir(B) transfectant are shown at only one exposure. Ub obtained post immunoprecipitation was also loaded along with eluted fractions. (E) Confocal microscopy images of cells stained with Brca1 antibody. Scale bars, 20 μm. Arrowheads point towards centrosomal localisation of Brca1. Nuclei were stained with DAPI. (F) CatRAPID-based prediction of interaction between Brca1 and Ginir represented as a map. Brca1, breast cancer type 1 susceptibility protein; Cep112, centrosomal protein 112; Ginir, Genomic Instability Inducing RNA; Giniras, antisense RNA to Ginir; IB, immunoblotted; Hotair, HOX transcript antisense RNA; IgG, immunoglobulin G; RNasin, RNase inhibitor; Ub, unbound fraction; XEF, Xenopus elongation factor. (TIF) [file pbio.2004204.s010.tif]

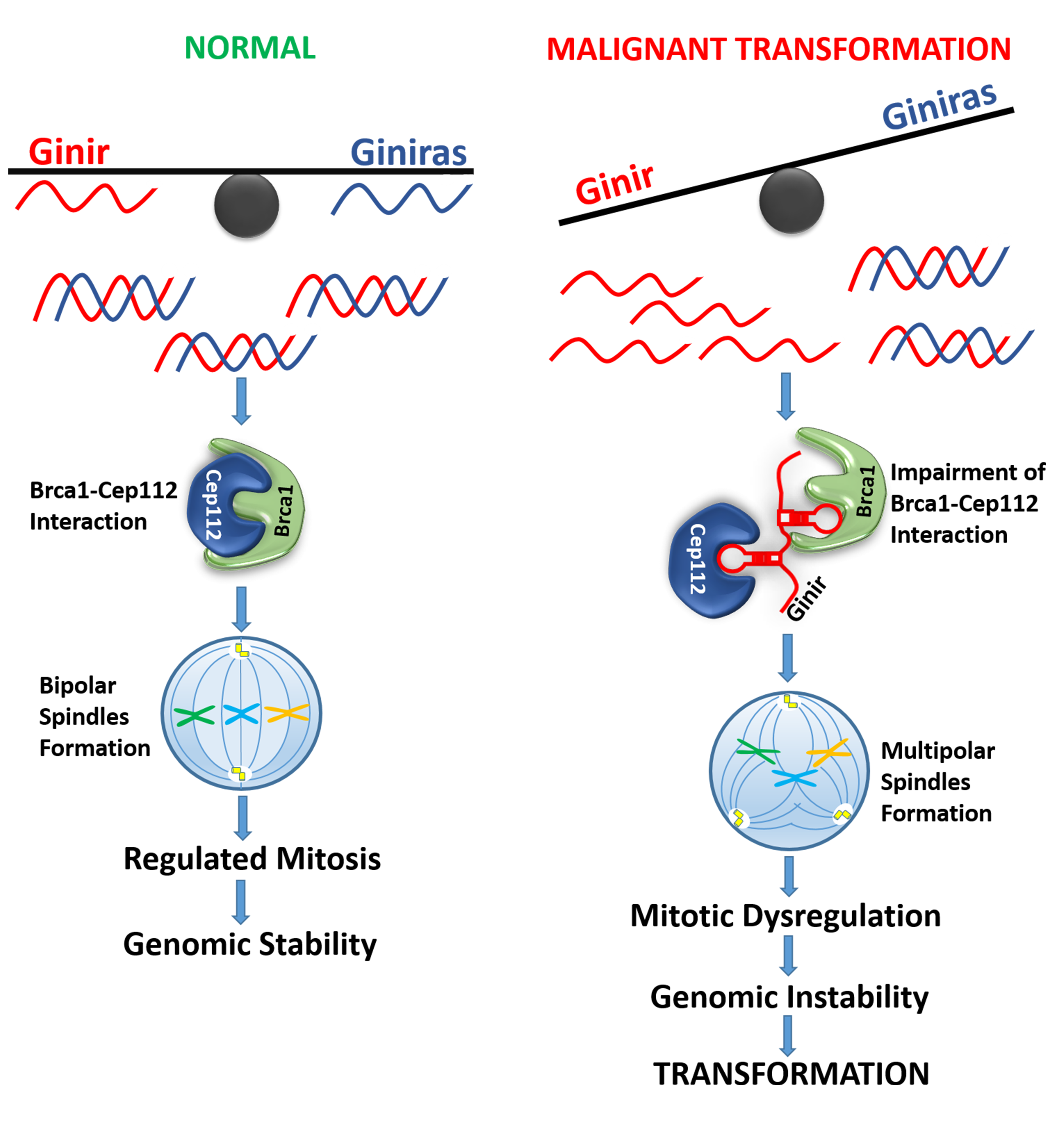

Supplement: S11 Fig — Excess levels of Ginir RNA cause imbalance in cellular homeostasis and propel cells towards malignant transformation. Ginir, Genomic Instability Inducing RNA; Giniras, antisense RNA to Ginir. (TIF) [file pbio.2004204.s011.tif]
