# Supplementary material for: Noncoding RNA Ginir functions as an oncogene by associating with centrosomal proteins
Source: PLoS Biol. 2018 Oct 8;16(10):e2004204. doi: 10.1371/journal.pbio.2004204 (PMC6193740; doi:10.1371/journal.pbio.2004204)
Supplement: S1 Text — Interacting protein partners of Ginir RNA identified by RNA affinity pull-down assay. Ginir, Genomic Instability Inducing RNA; MALDI-TOF, matrix-assisted laser desorption ionisation time-of-flight mass spectrometry. (DOCX) [file pbio.2004204.s012.docx]

**S1 Text:**


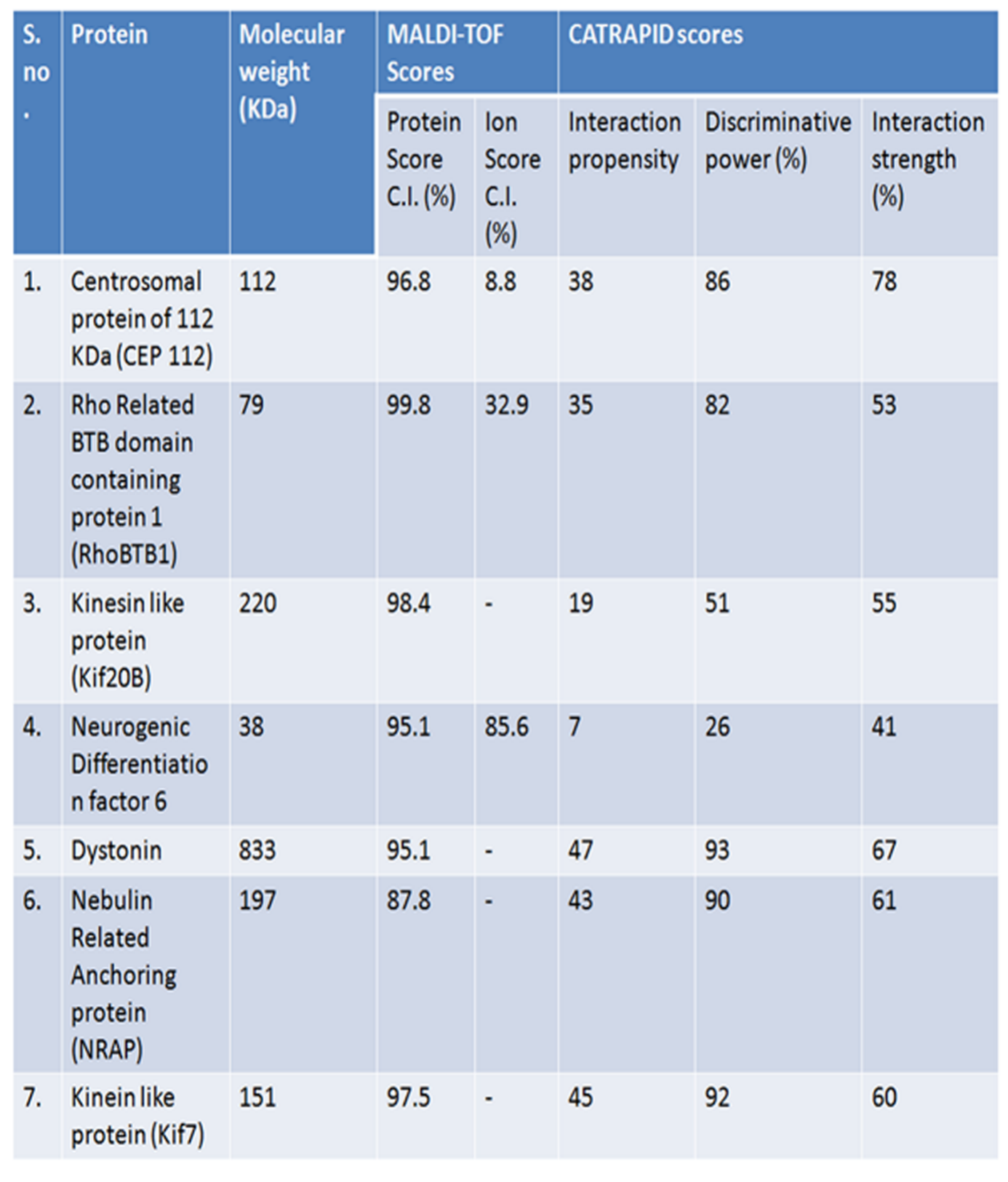


**S1 Text. MALDI-TOF & CatRAPID analysis for Ginir RNA- interacting proteins identified by RNA affinity pull-down assay.**
